# Comparative efficacy of exercise modalities added to energy restriction on inflammatory markers in obesity: a systematic review and network meta-analysis

**DOI:** 10.3389/fnut.2026.1879890

**Published:** 2026-08-05

**Authors:** Zhuang Ren, Tingfu Xia, Zhuce Shao, Shenqi Zhang

**Affiliations:** 1 The Engineering and Technical College of Chengdu University of Technology, Leshan, China; 2 School of Physical Education, Sichuan University, Chengdu, China; 3 Zaozhuang Municipal Hospital Affiliated to Jining Medical University, Zaozhuang, China

**Keywords:** energy restriction, exercise, inflammation, network meta-analysis, obesity

## Abstract

**Background:**

Chronic low-grade inflammation is a prominent feature of obesity, a major driver of cardiometabolic risk. While energy restriction (ER) is central to obesity treatment, the comparative effects of different exercise modalities added to ER on inflammatory biomarkers and insulin resistance remain unclear.

**Methods:**

We systematically searched for randomized controlled trials evaluating energy restriction (ER) alone, ER combined with aerobic exercise (ER+AE), high-intensity interval training (ER+HIIT), or combined exercise (ER+COE) in participants with overweight or obesity. The primary comparisons were ER+exercise interventions vs. ER alone. Treatment effects were quantified as standardized mean differences (SMDs) with 95% confidence intervals (CIs). Treatment hierarchy was explored using the Surface Under the Cumulative Ranking Curve (SUCRA) and interpreted as supportive rather than primary evidence.

**Results:**

Twenty-four randomized controlled trials involving 1,694 participants were included. Compared with ER alone, ER+AE yielded relatively steady incremental improvements across inflammatory endpoints, with favorable effects on CRP (SMD −0.52, 95% CI −0.97 to −0.07), hs-CRP (SMD −0.32, 95% CI −0.52 to −0.12), TNF-α (SMD −0.48, 95% CI −0.85 to −0.11), and IL-6 (SMD −0.35, 95% CI −0.64 to −0.06). For secondary outcomes, ER+COE was associated with a greater increase in adiponectin than ER alone (SMD 0.56, 95% CI 0.07 to 1.05), although this finding should be interpreted cautiously given the smaller evidence network. By contrast, none of the exercise-combined ER interventions showed statistically significant additional improvements in HOMA-IR compared with ER alone. No statistical evidence of global or local inconsistency was detected; however, the power to detect inconsistency was limited because several outcome networks were small or sparsely connected.

**Conclusions:**

When ER alone was used as the primary comparator, adding exercise produced biomarker-specific incremental benefits. ER+AE showed noticeable additional anti-inflammatory effects, particularly for hs-CRP, CRP, TNF-α, and IL-6. ER+COE was associated with increased adiponectin, whereas no exercise modality demonstrated a statistically significant additional benefit for HOMA-IR beyond ER alone. Overall, adjunctive exercise may provide targeted immunometabolic benefits during ER-based obesity treatment, but the current evidence does not support a single universally superior exercise modality.

**Systematic review registration:**

https://www.crd.york.ac.uk/PROSPERO/view/CRD420251175681, identifier CRD420251175681.

## 1 Introduction

Global obesity prevalence has climbed steeply across the past three decades and is forecast to maintain an upward trend over the next several decades (1, 2). Findings reported by the NCD Risk Factor Collaboration (NCD-RisC) show that the global number of people with obesity exceeded one billion in 2022, with 160 million being children and adolescents between 5 and 19 years of age (1). This obesity epidemic also imposes a tremendous health and economic toll; between 1990 and 2021, the global burden of mortality plus disability-adjusted life-years attributable to elevated body mass index rose over 2.5 times in both men and women (3), and overweight and obesity accounted for an estimated 2.19% of global GDP in 2019, with projections rising to 3.29% by 2060 under current trends (4). In line with this advancing body of knowledge, the 2025 commission report from Lancet Diabetes & Endocrinology introduced the framework of “clinical obesity”, which refers to a chronic adiposity-mediated disease featured by overt or impending organ damage linked to obesity, instead of excess body weight in isolation (5). Via a network of interrelated pathological mechanisms, excess adiposity promotes cardiometabolic disorders, including adipose tissue impairment, sustained low-grade inflammation, ectopic lipid storage, and reduced insulin responsiveness (6–8). A central mechanism is meta-inflammation, a persistent subclinical inflammatory state triggered by adipose expansion, hypoxia, and immune-cell remodeling, with prominent macrophage infiltration in visceral fat depots (6–12). Such an inflammatory milieu manifests with decreased anti-inflammatory adipose-derived cytokines (e.g., adiponectin) as well as increased pro-inflammatory factors encompassing interleukin-6 (IL-6) and tumor necrosis factor-α (TNF-α), ultimately worsening insulin resistance and cardiometabolic harm (8, 9, 13). Circulating indicators of metabolic status, among them high-sensitivity C-reactive protein (hs-CRP), tumor necrosis factor-α (TNF-α), IL-6, and adiponectin, accordingly offer clinically relevant readouts of obesity-driven cardiometabolic risk and act as vital targets for treatment (13–15).

Energy restriction (ER) stands as a core approach in managing obesity and reliably reduces body weight and ectopic fat (16). Evidence from randomized controlled trials and systematic reviews demonstrates that weight loss via dietary intervention reduces circulating hs-CRP; however, the changes in IL-6 and TNF-α levels exhibit greater heterogeneity, with fluctuations dependent on study cohort, dietary makeup, alongside participant compliance (17–22). However, ER alone may not fully restore immune homeostasis within adipose tissue, particularly in pathways related to TNF-α signaling and adipokine imbalance (8, 9, 17, 23). These limitations support combined strategies in which ER provides a common dietary background while exercise contributes additional anti-inflammatory and metabolic adaptations (23–26). Exercise triggers intricate cytokine responses: as an illustration, IL-6 plays a pro-inflammatory role in chronic disease conditions yet acts as a contraction-triggered myokine during physical activity, fostering anti-inflammatory signaling and substrate mobilization (27–29). Distinct modes of physical exercise—such as aerobic activity (AE), resistance exercise (RE), high-intensity interval training (HIIT), and combined exercise featuring both aerobic and resistance elements (COE)—generate specific physiological adjustments and may thus impact inflammatory biomarkers through differing pathways (30–33). Exercise-responsive exerkines and adaptive remodeling of adipose tissue, including processes linked to adipocyte browning, may further amplify these modality-specific effects (34–37).

Nevertheless, intervention trials combining ER with exercise have reported heterogeneous and sometimes conflicting findings for inflammatory biomarkers (23, 26). A key contributing factor is that the magnitude of attained energy deficit frequently differs across trials, thereby hindering the ability to disentangle the specific influence of exercise from the direct effects of dietary intake (16–22, 38–40). Recent network meta-analyses have compared exercise paradigms in people with overweight or obesity, but many included studies did not adequately control for dietary background, an important determinant of inflammatory status (40, 41).

For this reason, we limited the review to trials with a prespecified energy restriction (ER) protocol. Using a shared dietary background was intended to reduce confounding related to energy deficit and dietary composition and to improve the interpretability of indirect comparisons across exercise modalities. Even so, residual variation in ER implementation across studies could not be fully excluded (16, 40, 41).

In an effort to address these research gaps, we performed a systematic review and network meta-analysis of randomized controlled trials in which exercise modalities were delivered within an ER-based dietary background. We did not assume that ER prescriptions were identical across studies. Information on energy targets, dietary composition, monitoring procedures, and adherence verification was extracted where reported and used descriptively to contextualize transitivity. A hierarchical classification of dietary intervention platforms enabled sensitivity tests for the robustness of primary results (42–47). Our aims were to (i) quantify the incremental effects of AE, HIIT, and COE when added to ER on key inflammatory biomarkers, including hs-CRP, CRP, IL-6, and TNF-α, as well as adiponectin and HOMA-IR when data were available; (ii) explore the treatment hierarchy as a secondary, hypothesis-generating analysis; and (iii) examine whether participant age and intervention duration modified these effects (16, 17, 25, 40, 41). Comparisons with usual care or control groups were only applied to contextualize the overall magnitude of ER-based interventions.

## 2 Methods

### 2.1 Study protocol and reporting

We structured and reported the present work in compliance with the PRISMA 2020 framework, the PRISMA extension specific to network meta-analysis (PRISMA-NMA), and the PRISMA-S statement for formalizing search strategy reporting (44, 45, 48). The protocol was also aligned with PRISMA-P guidance (46) and was prospectively registered in PROSPERO before study screening began (CRD420251175681).

### 2.2 Inclusion and exclusion rules

#### 2.2.1 Eligibility criteria

(1) Randomized controlled trials (RCTs) represented the sole eligible study type.
(2) Participants exhibited overweight or obesity, or presented with metabolic syndrome. Excess body mass was defined based on body mass index (BMI)-based criteria specified by the trial authors, and metabolic syndrome was accepted when defined using established criteria such as NCEP ATP III or IDF-related consensus definitions (49, 50). Because age, metabolic syndrome, and other factors could act as effect modifiers, these characteristics were recorded for sensitivity analyses and exploratory effect-modification analyses.
(3) Studies had to compare ER combined with a structured exercise program of at least 2 weeks' duration, classifiable *a priori* as ER+AE, ER+COE, or ER+HIIT according to prespecified operational rules (Supplementary Table 2).
(4) All eligible studies compared ER plus exercise vs. ER alone or other eligible comparator regimens. Eligible comparator arms included usual care, habitual activity, and wait-list control groups. ER alone was specified *a priori* as the primary clinical reference for estimating incremental exercise effects. Therefore, the primary inferential contrasts were ER+AE, ER+HIIT, and ER+COE vs. ER alone, whereas comparisons vs. usual care/control were considered contextual. All eligible arms were retained in multi-arm trials comprising CON, ER alone, and ER+exercise cohorts. This strategy preserves the original randomized trial structure and precludes selective exclusion of trial arms.
(5) Eligible outcomes included inflammatory markers (CRP, TNF-α, IL-6) and additional inflammation-associated factors including adiponectin and HOMA-IR.

#### 2.2.2 Exclusion criteria

(1) We excluded any investigations that utilized non-randomized designs, including uncontrolled pre-post trials, protocols lacking empirical data, conference abstracts without full-text availability, or animal/*in vitro* research.
(2) Studies were excluded when they enrolled populations with conditions prespecified as likely to materially confound systemic inflammatory outcomes (e.g., diagnosed type 1/2 diabetes, autoimmune disease, active/recent cancer, severe renal/hepatic impairment, pregnancy/lactation, acute infection, or major surgery within 3 months).
(3) Studies were excluded when they evaluated pharmacological weight-loss agents, bariatric surgery, supplements, or multi-component interventions in which the incremental effect of adding exercise to ER could not be isolated.

### 2.3 Interventions and node definitions

Energy restriction (ER) was defined as structured dietary interventions for weight loss, classified into three strict subgroups: (A) explicit energy deficit, (B) fixed daily energy intake target, and (C) quantified hypoenergy diet protocols. We additionally flagged borderline/non-strict weight-management interventions that may lack quantifiable energy targets. These borderline interventions were included only in the broad primary ER network and excluded from strict-ER sensitivity analyses. Exercise regimens were classified *a priori* as AE, RT, HIIT, and COE in accordance with established training guidance and standardized HIIT principles (30, 51). To maximize intervention transparency and replicability, we extracted detailed descriptions of dietary and exercise protocols by utilizing TIDieR alongside the Consensus on Exercise Reporting Template (CERT) (52, 53). Table 1 displays the core attributes of the intervention nodes incorporated in the present network meta-analysis. Detailed operational rules for node allocation, including ER targets and exercise-intensity thresholds, are provided in Supplementary Table 2.

**Table 1:** Characteristics of the intervention nodes used in the network meta-analysis.

Node	Operational definition (*a priori*)
Control/usual care (CON)	No structured energy restriction or supervised training; usual advice, habitual activity, or wait-list control. Minimal-contact controls were allowed
Energy restriction (ER)	Prescribed hypoenergy diet or structured dietary program intended to reduce energy intake, with or without behavioral support
ER + aerobic exercise (ER+AE)	ER plus structured aerobic or endurance exercise prescribed by frequency, duration, and intensity, typically at moderate-to-vigorous intensity
ER + high-intensity interval training (ER+HIIT)	ER combined with interval exercise regimens consisting of cycles of intense effort separated by rest intervals
ER + combined exercise (ER+COE)	ER combined with a regimen integrating cardio and strength exercise, administered during a single workout or conducted as distinct scheduled training blocks

Detailed node-mapping rules, including supervision, intensity thresholds, and the handling of multi-component interventions, are provided in Supplementary Table 2; full search strategies are provided in Supplementary Table 3.

To standardize intervention categorization and strengthen transparency in ER implementation, hierarchical ER definitions were formulated per predefined criteria. Interventions with defined energy deficits, fixed daily energy intake targets or quantified hypoenergy protocols were classified as strict ER. This category formed the basis of targeted strict-sensitivity analyses.

Weight-management interventions that may lack quantifiable energy benchmarks were labeled borderline/non-strict ER and considered lower in intensity relative to strict ER. Trial arms without active dietary weight-loss interventions or only general dietary advice were coded as usual care/control (CON). The CON node was retained within the network to preserve the original randomized trial structure and sustain network connectivity. This comparator also provided contextual data quantifying the overall efficacy of structured ER interventions relative to usual care or sustained habitual activity. Accordingly, CON-referenced estimates were interpreted only as contextual evidence and not as incremental exercise effects beyond ER alone.

For each study arm, transitivity-related implementation data were extracted, covering energy targets, dietary composition, monitoring and adherence assessment metrics, as well as exercise frequency, intensity, duration, modality, supervision, intensity validation and exercise adherence indicators (42, 52, 53). Strict and borderline ER arms were both included in the main broad ER network. This was because both represented active dietary weight-loss interventions tested in the original randomized trials. All borderline ER entries were documented separately in Supplementary Table 2. These borderline interventions were excluded from strict-ER sensitivity analyses to partially mitigate heterogeneity bias caused by inconsistent dietary implementation. AE, RT, HIIT and COE were pre-specified as the exercise modalities for analysis. Owing to limited trials examining ER plus isolated RT, the final network only contained ER monotherapy and ER combined with AE, HIIT or COE, with resistance training incorporated solely within the combined-exercise node instead of functioning as an independent intervention arm.

### 2.4 Information sources and search protocols

We performed a comprehensive literature search across PubMed/MEDLINE, Embase, Web of Science, SPORTDiscus, and the Cochrane Library. This search covered the timespan from January 1, 2000, to January 10, 2026, and was carried out with no constraints on publication language. Our literature search employed a blend of controlled vocabulary items and free-text keywords related to energy restriction or dietary weight loss, exercise modalities, and inflammatory markers. Supplementary Table 3 details the complete search strategies utilized.

Ren and Shao collaborated to formulate the search strategy. Additionally, we manually checked the bibliographies of related review papers to uncover further qualifying trials that the database search failed to detect.

### 2.5 Trial screening and data abstraction

Following the removal of duplicate records, two researchers (Xia and Ren) conducted independent assessments of titles, abstracts, and full-text articles. Any discrepancies were settled via mutual agreement or upon deliberation with an additional reviewer (Shao) (42). Pairs of reviewers separately gathered key study attributes (including study design, participant count, age profile, gender breakdown, and concurrent conditions), specifics of the study interventions (such as dietary guidelines; exercise frequency, intensity, session length, modality, progression, and oversight), as well as endpoint measurements (52, 53). In cases where several post-intervention assessment time points were documented, we extracted measurements obtained at the conclusion of the active intervention period to reduce variability linked to long-term upkeep stages (42). Regarding crossover study designs, we exclusively utilized data derived from the initial randomization phase, following the recommendations set forth by the Cochrane guidelines (42). The predefined outcome measures encompassed CRP/hs-CRP, IL-6, TNF-α, adiponectin, and HOMA-IR. In instances where datasets were absent or inadequately documented, we made efforts to correspond with the original study investigators.

Because inflammatory markers were measured using different laboratory methods, reported units, and follow-up schedules, we extracted data from the final time point of the active intervention phase. We preserved the original definitions of biomarkers as documented by each trial's investigators and employed standardized mean differences to adjust for disparities across diverse assessment metrics. This approach was intended to improve comparability across studies, not to imply that the underlying assays were equivalent (54, 55).

For outcome extraction, post-intervention endpoint values were used as the primary data source because these were the most consistently reported across the eligible trials. When both endpoint and change-score data were reported, endpoint values were retained for the primary analysis to maintain a consistent data structure across the network. For studies reporting multiple follow-up assessments, we extracted the measurement obtained at the end of the active intervention phase. For crossover trials, only data from the first randomized period were used. For each biomarker, we extracted the reported assay information, measurement unit, fasting status, and blood-sampling timing when available (Supplementary Table 4). hs-CRP and CRP were treated as related but separate outcomes. When multiple identical biomarker measurements were reported at one timepoint, only the value matching the predefined outcome definition was selected to avoid duplicate effect estimates per study within each outcome analysis.

### 2.6 Evaluation of risk of bias and evidence quality rating

Two researchers (Zhang and Shao) conducted independent appraisals of methodological rigor by employing the Cochrane RoB 2 instrument. Any inconsistencies were settled via joint deliberation alongside an additional reviewer (47). Confidence in each network estimate was evaluated using a GRADE-consistent approach operationalized through the CINeMA framework and web application (56–59).

We used CINeMA to appraise the certainty of network effect estimates, but not as a criterion for excluding studies. Assessment was conducted with reference to internal study bias, indirectness, statistical imprecision, between-study heterogeneity, and inconsistency. Assessment also incorporated the impact of direct and indirect comparative evidence throughout the entire network structure (56–60). These assessments were used to interpret the strength of statistically significant findings.

### 2.7 Data integration and statistical evaluations

#### 2.7.1 Outcome data and effect measures

Post-intervention endpoint means collected right after the intervention phase served as primary data for quantitative synthesis. This metric was chosen due to its consistent availability across all included trials. Baseline values were extracted where available and considered when assessing risk of bias, transitivity, and the clinical interpretation of pooled estimates. When important baseline imbalance was apparent, this was considered in the risk-of-bias and certainty assessments, and the robustness of the corresponding estimates was examined in sensitivity analyses when connected data permitted. Given that inflammatory and metabolic biomarkers were measured using different assay platforms and reported in different units or scales, Hedges' g standardized mean difference (SMD), with corresponding 95% confidence intervals (CIs), was used as the primary effect measure (42, 54, 55).

When standard deviations were not directly reported, they were derived, where possible, from standard errors, confidence intervals, *p* values, t statistics, interquartile ranges, ranges, or other available summary statistics according to Cochrane Handbook guidance (42). If SDs could not be derived reliably, the corresponding study-outcome data were excluded from the quantitative synthesis for that outcome. The original reported data type and the corresponding conversion or SD-derivation method were documented in Supplementary Table 4.

CRP, hs-CRP, IL-6 and TNF-α typically exhibit right-skewed distributions, hence log transformation is routinely applied for pooled analyses. However, most eligible trials reported arithmetic means and standard deviations, whereas geometric means, log-transformed means, log-scale standard deviations, or sufficient information to reconstruct log-scale estimates were not consistently available. Therefore, SMDs based on reported endpoint values were retained as the primary effect measure. Residual measurement heterogeneity related to assay method, fasting status, and blood-sampling timing was considered when interpreting the results. Corresponding descriptive statistics are summarized in Supplementary Table 4.

#### 2.7.2 Pairwise and network meta-analysis

For trials reporting multiple follow-up assessments, only the time point corresponding to the end of the active intervention period was included. Each study contributed only one dataset per biomarker to each analysis, and hs-CRP and CRP were treated as related but separate outcomes. For multi-arm trials, all eligible intervention arms were retained within the same network model. Comparisons sharing a common study arm were treated as correlated rather than independent comparisons, and shared control or comparator arms were entered only once at the arm-data level to avoid double counting. For multi-arm trials, all eligible study arms were retained within the same network model. Comparisons sharing a common study arm were treated as correlated rather than independent comparisons, and shared control or comparator arms were entered only once at the arm-data level to avoid double counting.

Pairwise meta-analyses were first conducted to summarize direct evidence, with heterogeneity assessed using Cochran's Q test and the I^2^ statistic (61, 62). These pairwise analyses were used to provide a descriptive summary of direct evidence, whereas the primary inferential analysis was based on the random-effects network meta-analysis. Pairwise analyses were performed using Review Manager software version 5.4 (69). Random-effects network meta-analyses were then conducted using the netmeta package in R statistical software, version 4.5.2. The network models assumed a common between-study heterogeneity variance (τ^2^) across comparisons and accounted for correlations arising from multi-arm trials (63). Network structure and the contribution of direct comparisons were described using network plots and evidence-flow metrics (60, 63). The CON node was included in the network model and could contribute indirect evidence to ER-referenced estimates depending on the outcome-specific network geometry. To avoid overinterpreting effect estimates shaped by indirect pathways through CON, we synthesized multiple lines of evidence for ER-referenced effect sizes. These included direct pairwise comparisons against ER, the partitioning of direct vs. indirect evidence, and certainty evaluations. Conclusions regarding incremental exercise effects were based on contrasts between ER+exercise interventions and ER alone. Comparisons vs. usual care or control served merely as supplementary contextual evidence.

#### 2.7.3 Transitivity, inconsistency, and treatment hierarchy

Transitivity was evaluated by comparing the distribution of prespecified potential effect modifiers across treatment comparisons, including age category, baseline BMI, metabolic phenotype, intervention duration, ER prescription type, exercise intensity, and supervision status (42, 64). Global and local inconsistency were assessed where feasible using design-by-treatment interaction and side-splitting approaches (64–66). When inconsistency tests did not provide statistical evidence against the consistency assumption, consistency models were used for the main analyses. however, non-significant inconsistency tests were interpreted cautiously because several outcome networks were small or sparsely connected.

Treatment hierarchy was assessed using the Surface Under the Cumulative Ranking Curve (SUCRA). Treatment-ranking probabilities were generated using Stata/MP 15.0 and exported to R for SUCRA calculation and ranking-figure generation. The direction of benefit was specified *a priori*: lower values were considered favorable for hs-CRP, CRP, IL-6, TNF-α, and HOMA-IR, whereas higher values were considered favorable for adiponectin. SUCRA values were interpreted as exploratory hierarchy indicators and were considered alongside effect sizes, 95% CIs, prediction intervals, direct and indirect evidence contribution, network connectivity, and certainty of evidence rather than as stand-alone evidence of treatment superiority (58, 64, 67).

Potential small-study effects were examined using comparison-adjusted funnel plots and network-appropriate regression methods when a sufficient number of studies was available (68).

#### 2.7.4 Population heterogeneity, sensitivity analyses, and robustness evaluation

To address population heterogeneity and generalizability, included trials were classified at the study level according to age category, metabolic phenotype, and intervention duration. Age category was coded as minors, adults, or older adults according to the trial population and reported mean age. Metabolic phenotype was classified as uncomplicated overweight/obesity or metabolic syndrome, or elevated cardiometabolic risk according to the original eligibility criteria. Intervention duration was classified as short-term or longer-term according to the prespecified study-level classification. Detailed coding rules and descriptive information on population and intervention characteristics are presented in Supplementary Table 4.

Prespecified exploratory effect-modification analyses were conducted using sensitivity analyses and network meta-regression where data permitted, focusing on participant age and intervention duration (42, 64). Sensitivity analyses were conducted where adequate connected data existed. Sensitivity analyses excluded borderline/non-strict ER trials, minor participants, short-duration interventions, low-baseline BMI cohorts, populations with metabolic syndrome or elevated cardiometabolic risk, and studies with high overall bias risk. Fixed-effect network models were also examined as alternative analytical models (42, 64). Because these restrictions were applied at the study level and several networks became sparse or disconnected after filtering, sensitivity and effect-modification analyses were interpreted as exploratory and hypothesis-generating rather than confirmatory subgroup analyses. Unless otherwise specified, all statistical tests were two-sided, with an alpha threshold of 0.05.

## 3 Results

### 3.1 Literature screening and network characteristics

**Figure 1:**
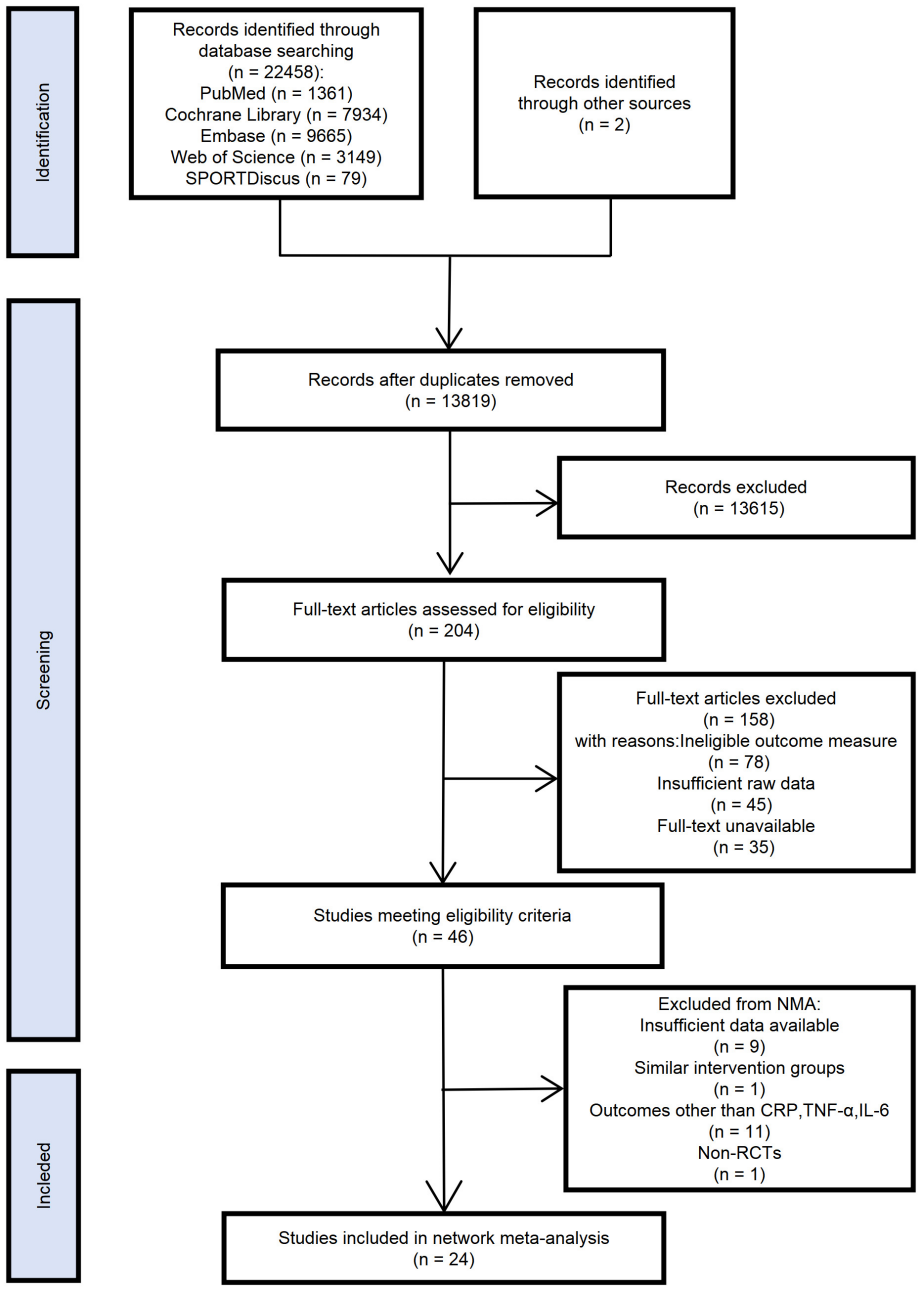
PRISMA flow diagram.

A total of 22,460 relevant citations were retrieved in this work. Among them, 22,458 were obtained from electronic databases, and another 2 came from manual literature browsing. Following the deletion of redundant entries, 13,819 non-duplicate citations were kept for preliminary screening. During the title and abstract evaluation phase, 13,615 irrelevant citations were ruled out. Subsequently, 204 full-text publications were further reviewed against the inclusion and exclusion standards. Of these full-text papers, 158 were eliminated for the following reasons: inappropriate outcome indicators (*n* = 78), lack of available original data (*n* = 45), and inaccessibility of complete manuscript content (*n* = 35). This left 46 investigations satisfying the preset inclusion standards. From the qualified literature, 22 were additionally excluded prior to network meta-analysis. The reasons included inadequate data for quantitative pooling (*n* = 9), overlapping intervention arms (*n* = 1), failure to report CRP, TNF-α, or IL-6 endpoints (*n* = 11), as well as non-randomized controlled trial design (*n* = 1). Ultimately, 24 eligible studies were included in the network meta-analysis. The overall literature screening procedure is presented in the PRISMA flow chart (Figure 1). Twenty-four eligible RCTs (*n* = 1,694; sample size per trial 12–335) were included (18–22, 33, 38, 39, 70–85). Ten of the eligible trials contained a control arm receiving ER alone (diet-only intervention). Five three-arm trials additionally incorporated three groups: usual care/maintenance of routine activity, ER alone, and ER plus exercise. These trials yielded direct ER-referenced evidence while preserving their original randomized control arm. Nine trials compared ER+exercise with usual care or habitual-activity. The control node was retained to sustain network connectivity and generate contextual supporting evidence. All primary inferential analyses, however, were confined to ER-centered comparative contrasts. CRP/hs-CRP was the most frequently reported biomarker (18 studies), followed by IL-6 (14 studies) and TNF-α (9 studies). Among secondary outcomes, HOMA-IR was reported in 11 trials and adiponectin in 7. Other prespecified biomarkers, such as IL-8, IL-10, and leptin, were too sparsely reported for quantitative synthesis.

The included studies were clinically heterogeneous. These trials recruited young individuals including children and teenagers, adults across young to middle age groups, elderly populations, and those diagnosed with metabolic syndrome. The length of the intervention periods spanned between 2 weeks and 18 months (Supplementary Table 4). Such variations were accounted for when evaluating transitivity and performing the predefined exploratory analyses of effect modification.

### 3.2 Transitivity

The transitivity assumption was evaluated via review of inclusion criteria for all eligible trials.

Distributions of pre-specified effect modifiers across intervention nodes were additionally compared, including participant age, baseline BMI, metabolic syndrome status, intervention duration, ER type, exercise frequency, intensity and supervision (42, 64). The distribution of these factors is summarized in Supplementary Table 5. Overall, the transitivity assumption was considered plausible because the included trials enrolled participants with overweight or obesity and the exercise-combined interventions were delivered within an ER-based dietary framework. However, residual clinical heterogeneity remained, particularly in age category, metabolic syndrome status, intervention duration, degree of ER quantification, dietary monitoring, and exercise supervision. Therefore, transitivity was considered reasonable but not definitive, and indirect comparisons were interpreted cautiously. These potential effect modifiers were further examined through predefined sensitivity analyses and exploratory meta-regression where data permitted.

### 3.3 Risk of bias assessment

Using the RoB 2 assessment tool, 10 trials were classified as having a low risk of bias, 10 studies presented some concerns, and 4 showed a high risk of bias with regard to the primary outcome measures (47). The most frequent issues stemmed from nonadherence to the planned intervention protocols and missing outcome measurements. Confidence in the NMA findings was further appraised using CINeMA 2.0 (Supplementary Figure 1). We also conducted sensitivity analyses that omitted high-risk trials to test the robustness of our findings.

CINeMA showed a clear pattern in confidence ratings. Comparisons involving the well-sampled control group and the ER+AE node generally had higher confidence, whereas estimates for HIIT and COE were often less certain because they were based on fewer studies and less direct evidence (Supplementary Figure 1).

### 3.4 Network geometry

**Figure 2:**
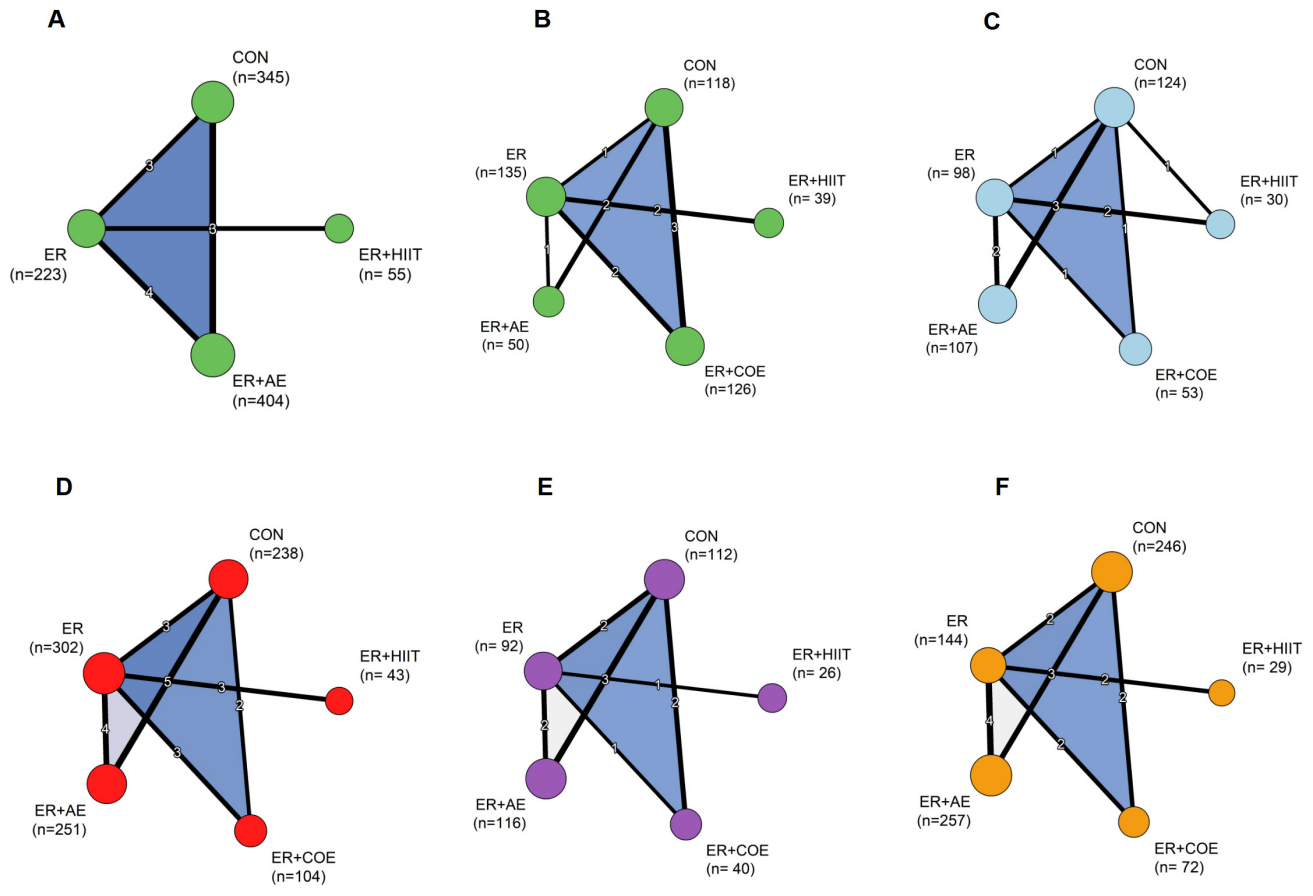
Network of eligible comparisons for different inflammation outcomes. **(A)** hs-CRP (*n* = 10 studies), **(B)** CRP (*n* = 9 studies), **(C)** TNF-α (*n* = 9 studies), **(D)** IL-6 (*n* = 14 studies), **(E)** adiponectin (*n* = 7 studies), and **(F)** HOMA-IR (*n* = 11 studies). Line width is proportional to the number of trials informing each comparison, node size is proportional to the number of participants, and blue shading indicates the presence of three-arm studies. CON, control; ER, energy restriction; ER+AE, energy restriction with aerobic training; ER+HIIT, energy restriction with high-intensity interval training; ER+COE, energy restriction with aerobic and resistance exercise.

The networks were primarily anchored by the ER and ER+AE, which provided the largest volume of direct evidence (as illustrated in Figure 2). ER+HIIT and ER+COE were represented by fewer direct comparisons. Outcome-specific network geometry, including the number of studies and participants, network nodes and edges, available direct comparisons, and direct/indirect evidence support for ER-referenced comparisons, is summarized in Table 2. Across the 24 RCTs, ER+AE was the most frequently studied intervention (13/24), followed by ER+COE (6/24) and ER+HIIT (5/24). The length of the interventions varied between 2 weeks and 18 months, and the median duration stood at roughly 12 to 13 weeks. To address heterogeneity in the dietary background, we re-examined ER implementation at the study level and summarized the details in Supplementary Table 2. For each study, we extracted the reported energy deficit or intake target, dietary composition, monitoring or adherence procedures, and whether dietary delivery was actively supervised or mainly prescribed. Most studies used a quantified ER prescription, including explicit daily energy deficits, fixed kcal targets, percentage energy restriction, very-low-energy diets, meal replacements, or structured hypoenergy menus. A smaller group of studies provided structured dietary interventions but did not clearly report a quantified energy deficit or fixed kcal target; these were labeled as borderline/non-strict ER and were excluded in the strict-ER sensitivity analysis. Therefore, ER implementation was not assumed to be identical across studies, and residual differences in dietary intensity, composition, monitoring, and adherence were considered when evaluating transitivity and interpreting exercise-specific effects. ER was typically implemented using prescribed hypoenergy diets or explicit energy targets/deficits, and exercise prescriptions usually involved 2–5 sessions per week. Where reported, HIIT protocols generally targeted ~80–95% of peak or maximum heart rate, and COE protocols combined aerobic and resistance components. Supervision was variably reported, with 10 studies explicitly describing supervised sessions.

**Table 2:** Outcome-specific network geometry and evidence contribution for ER-referenced comparisons.

Outcome	Studies/ *N*	Nodes/ edges	Direct comparisons (k)	ER+AE vs. ER	ER+HIIT vs. ER	ER+COE vs. ER	Model diagnostics
hs-CRP	10/1,027	4/4	CON–ER(k = 3); CON–ER+AE(k = 6); ER–ER+AE(k = 4); ER–ER+HIIT(k = 3)	k = 4; *n* = 338 D/I = 64.4%/35.6%	k = 3; *n* = 108 D/I = 100.0%/0.0%	Absent	τ^2^ = 0.0000 I^2^ = 12.5%; Qp = 0.325
CRP	9/468	5/6	CON–ER(k = 1); CON–ER+AE(k = 2); CON–ER+COE(k = 3); ER–ER+AE(k = 1); ER–ER+HIIT(k = 2); ER–ER+COE(k = 2)	k = 1; *n* = 36 D/I = 49.1%/50.9%	k = 2; *n* = 77 D/I = 100.0%/0.0%	k = 2; *n* = 161 D/I = 67.4%/32.6%	τ^2^ = 0.0000 I^2^ = 0.0%; Qp = 0.951
TNF-α	9/412	5/7	CON–ER(k = 1); CON–ER+AE(k = 3); CON–ER+HIIT(k = 1) CON–ER+COE(k = 1); ER–ER+AE(k = 2); ER–ER+HIIT(k = 2); ER–ER+COE(k = 1)	k = 2; *n* = 46 D/I = 42.4%/57.6%	k = 2; *n* = 44 D/I = 80.0%/20.0%	k = 1; *n* = 106 D/I = 59.0%/41.0%	τ^2^ = 0.0000 I^2^ = 0.0%; Qp = 0.441
IL-6	14/938	5/6	CON–ER(k = 3); CON–ER+AE(k = 5); CON–ER+COE(k = 2); ER–ER+AE(k = 4); ER–ER+HIIT(k = 3); ER–ER+COE(k = 3)	k = 4; *n* = 320 D/I = 62.3%/37.7%	k = 3; *n* = 83 D/I = 100.0%/0.0%	k = 3; *n* = 205 D/I = 68.3%/31.7%	τ^2^ = 0.0429 I^2^ = 27.6%; Qp = 0.160
Adiponectin	7/386	5/6	CON–ER(k = 2); CON–ER+AE(k = 3); CON–ER+COE(k = 2); ER–ER+AE(k = 2); ER–ER+HIIT(k = 1); ER–ER+COE(k = 1)	k = 2; *n* = 94 D/I = 58.9%/41.1%	k = 1; *n* = 52 D/I = 100.0%/0.0%	k = 1; *n* = 44 D/I = 47.0%/53.0%	τ^2^ = 0.0000 I^2^ = 0.0%; Qp = 0.679
HOMA-IR	11/748	5/6	CON–ER(k = 2); CON–ER+AE(k = 3); CON–ER+COE(k = 2); ER–ER+AE(k = 4); ER–ER+HIIT(k = 2); ER–ER+COE(k = 2)	k = 4; *n* = 140 D/I = 68.4%/31.6%	k = 2; *n* = 56 D/I = 100.0%/0.0%	k = 2; *n* = 99 D/I = 64.6%/35.4%	τ^2^ = 0.0455 I^2^ = 13.4%; Qp = 0.320

Values are presented as number of studies / number of participants. Nodes/edges indicate the number of treatment nodes and direct comparison edges in each outcome-specific network. k indicates the number of studies informing a direct comparison, and n indicates the summed arm-level sample size for the corresponding direct comparison. D/I indicates the percentage contribution of direct and indirect evidence to the corresponding ER-referenced network estimate. τ^2^ denotes the between-study variance, I^2^ denotes the proportion of variability attributable to heterogeneity, and Qp denotes the *p* value for the heterogeneity Q statistic. These contribution percentages describe the evidence structure and should not be interpreted as certainty of evidence or magnitude of treatment effect. CON, control/usual care; ER, energy restriction; ER+AE, ER plus aerobic exercise; ER+HIIT, ER plus high-intensity interval training; ER+COE, ER plus combined exercise.

ER+AE occupied the central position in the network and contributed the largest volume of direct evidence across outcomes. By contrast, ER+HIIT and ER+COE were supported by fewer direct comparisons and, for some outcomes, relied more heavily on indirect evidence (Supplementary Figure 2). As summarized in Table 3, the certainty of evidence varied across outcomes and comparisons. Because the primary clinical question focused on the incremental effects of adding exercise to ER, ER-referenced comparisons were prioritized. Comparisons against the control are provided as contextual supporting evidence (Supplementary Table 6). Overall, evidence was more informative for ER+AE than for ER+HIIT or ER+COE. Several ER+HIIT and ER+COE comparisons were downgraded because of imprecision, sparse evidence, within-study bias, or heterogeneity. Therefore, the certainty of evidence for several key comparisons was low or very low, indicating that these estimates should be interpreted cautiously.

**Table 3:** Summary of findings and certainty of evidence for key ER-referenced network comparisons.

Outcome	Comparison	No. of direct studies	SMD (95% CI)	Certainty	Main reasons for downgrading	Interpretation
hs-CRP	ER+AE vs. ER	4	−0.32 (–0.52, –0.12)	Moderate	Heterogeneity	Favors ER+AE
hs–CRP	ER+HIIT vs. ER	3	–0.11 (–0.48, 0.26)	**Very low**	Within*-*study bias; Imprecision	No clear difference; very uncertain
CRP	ER+AE vs. ER	1	–0.52 (–0.97, –0.07)	Low	Within*-*study bias	Favors ER+AE
CRP	ER+HIIT vs. ER	2	–0.31 (–0.75, 0.13)	**Very low**	Within*-*study bias; Imprecision; Heterogeneity	No clear difference; very uncertain
CRP	ER+COE vs. ER	2	0.07 (–0.22, 0.36)	Low	Imprecision	No clear difference
TNF–α	ER+AE vs. ER	2	–0.48 (–0.85, –0.11)	High	No major downgrading concerns	Favors ER+AE
TNF–α	ER+HIIT vs. ER	2	0.20 (–0.32, 0.72)	**Very low**	Imprecision; Incoherence	No clear difference; very uncertain
TNF-α	ER+COE vs. ER	1	–0.02 (–0.39, 0.34)	Low	Imprecision	No clear difference
IL*-*6	ER+AE vs. ER	4	–0.35 (–0.64, –0.06)	Moderate	Heterogeneity	Favors ER+AE
IL-6	ER+HIIT vs. ER	3	–0.27 (–0.77, 0.23)	**Very low**	Within–study bias; Imprecision	No clear difference; very uncertain
IL-6	ER+COE vs. ER	3	0.09 (–0.26, 0.43)	Low	Imprecision	No clear difference
Adiponectin	ER+AE vs. ER	2	–0.02 (–0.36, 0.32)	Low	Imprecision	No clear difference
Adiponectin	ER+HIIT vs. ER	1	–0.31 (–0.85, 0.22)	**Very low**	Within–study bias; Imprecision	No clear difference; very uncertain
Adiponectin	ER+COE vs. ER	1	0.56 (0.07, 1.05)	Moderate	Heterogeneity	Favors ER+COE
HOMA*-*IR	ER+AE vs. ER	4	–0.22 (–0.57, 0.12)	Moderate	Imprecision	No clear difference
HOMA*-*IR	ER+HIIT vs. ER	2	–0.35 (–0.96, 0.26)	**Very low**	Within*-*study bias; Imprecision; Heterogeneity	No clear difference; very uncertain
HOMA*-*IR	ER+COE vs. ER	2	–0.28 (–0.70, 0.15)	Low	Within-study bias; Imprecision; Heterogeneity	No clear difference

The evidence summary and certainty assessments presented in this table were produced via the CINeMA platform for network meta-analysis. Because the primary clinical question concerned the incremental effect of adding exercise to ER, this table prioritizes ER-referenced comparisons. Contextual comparisons vs. CON, including prediction intervals, are provided in Supplementary Table 6.

### 3.5 Direct pairwise meta-analyses

Direct pairwise meta-analyses suggested modality-specific patterns for inflammatory outcomes.

#### 3.5.1 Hs-CRP/CRP

**Figure 3:**
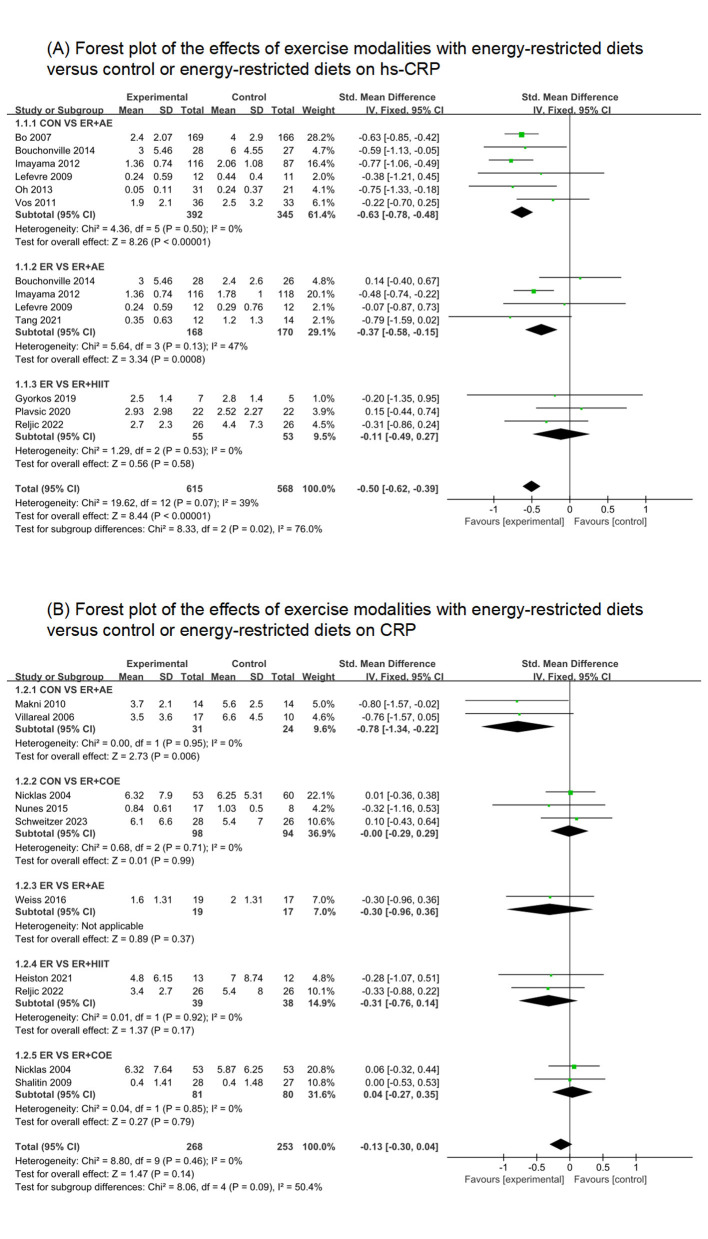
Forest plots of direct comparisons for exercise modalities combined with energy-restricted diets on hs-CRP and CRP. **(A)** hs-CRP. **(B)** CRP.

Pairwise meta-analysis results for hs-CRP/CRP are displayed in Figure 3. For hs-CRP, ER+AE significantly reduced concentrations vs. both ER alone (SMD −0.37, 95% CI −0.58 to −0.15, *p* = 0.0008, I^2^ = 47%) and control (SMD −0.63, 95% CI −0.78 to −0.48, *p* < 0.0001, I^2^ = 0%). By contrast, ER+HIIT exerted no statistically significant lowering effect on hs-CRP levels vs. ER alone (SMD −0.11, 95% CI −0.49 to 0.27, *p* = 0.58, I^2^ = 0%). For CRP, ER+AE also reduced values vs. control (SMD −0.78, 95% CI −1.34 to −0.22, *p* = 0.006, I^2^ = 0%), although only one head-to-head trial compared ER+AE with ER alone. Statistically significant direct effects were not observed for other types of exercise interventions.

#### 3.5.2 Cytokines

**Figure 4:**
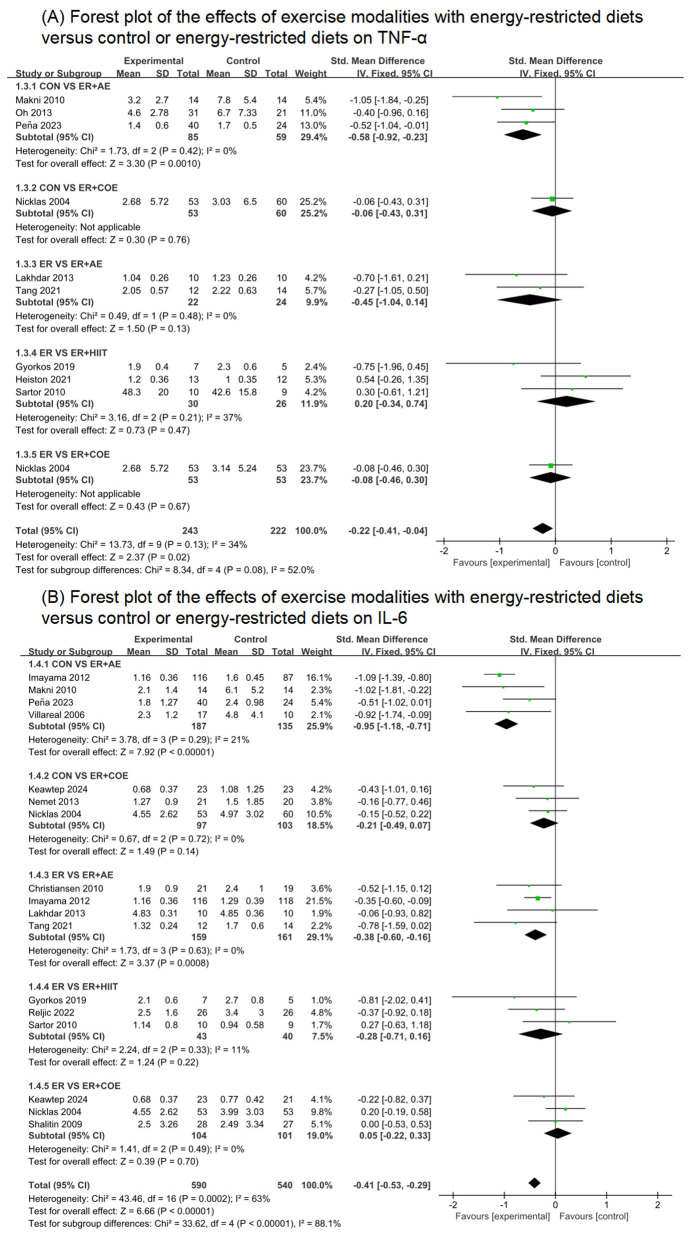
Forest plots of direct comparisons for exercise modalities combined with energy-restricted diets on cytokines. **(A)** TNF-α. **(B)** IL-6.

Pairwise meta-analysis results for cytokines are displayed in Figure 4. For TNF-α, ER+AE yielded lower concentrations relative to ER alone; however, this intergroup difference was not statistically significant. Compared with control, ER+AE was associated with lower TNF-α concentrations (SMD −0.58, 95% CI −0.92 to −0.23, *p* = 0.001, I^2^ = 0%). For IL-6, ER+AE elicited significant reductions in IL-6 concentrations relative to ER alone (SMD −0.38, 95% CI −0.60 to −0.16, *p* = 0.0008, I^2^ = 0%). Compared with the control group, ER+AE also produced the most substantial absolute reduction in IL-6 (SMD −0.95, 95% CI −1.18 to −0.71, *p* < 0.0001, I^2^ = 21%). Direct contrasts between other exercise approaches failed to demonstrate any significant statistical differences.

#### 3.5.3 Secondary outcomes

**Figure 5:**
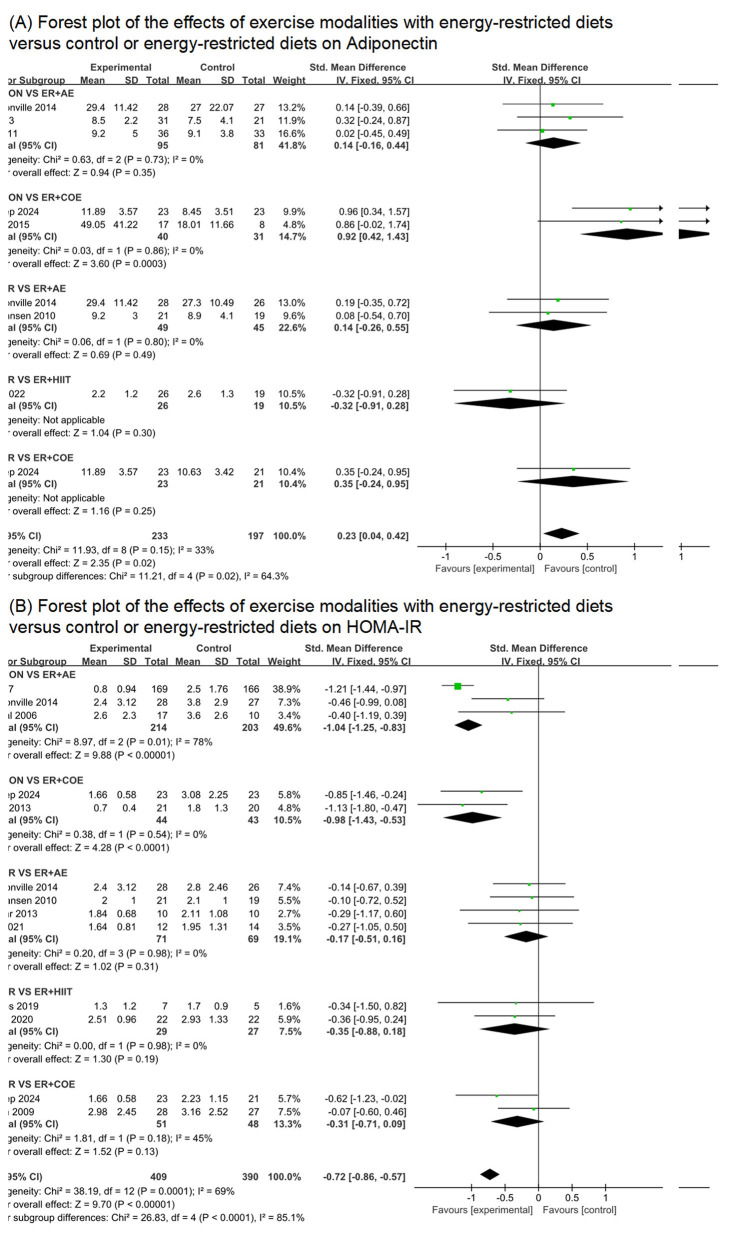
Forest plots of direct comparisons for exercise modalities combined with energy-restricted diets on secondary outcomes. **(A)** adiponectin. **(B)** HOMA-IR.

Findings from pairwise meta-analyses on secondary endpoints are presented in Figure 5. For adiponectin, ER+COE significantly increased concentrations vs. control (SMD 0.92, 95% CI 0.42 to 1.43, *p* = 0.0003, I^2^ = 0%), whereas other direct comparisons were not statistically significant; only one direct trial compared an exercise-combined ER intervention with ER alone. For HOMA-IR, none of the three exercise-combined ER interventions produced statistically significant additional reductions compared with ER alone. Nevertheless, both ER+COE (SMD −0.98, 95% CI −1.43 to −0.53, *p* < 0.0001, I^2^ = 0%) and ER+AE (SMD −1.04, 95% CI −1.25 to −0.83, *p* < 0.0001, I^2^ = 78%) reduced HOMA-IR compared with control. The heterogeneity detected in several direct comparisons supported the use of network meta-analysis and further exploration of potential effect modifiers.

### 3.6 Network meta-analysis

Findings from the random-effects network meta-analysis are outlined below; the league table presenting pooled SMDs for exercise interventions paired with energy-restricted diets is displayed in Table 4. The relative weightings of direct vs. indirect evidence, alongside the count of trials included in every pairwise contrast, are presented in Supplementary Figure S2. Visual forest representations detailing both primary and secondary endpoint results, which incorporate both 95% CIs and corresponding 95% prediction ranges, are exhibited in Supplementary Figure S3.

**Table 4:** League table of pooled standardized mean differences (SMDs) for exercise modalities with energy-restricted diets (outcomes: hs-CRP, CRP, TNF-α, IL-6, adiponectin, HOMA-IR).

hs-CRP				
**ER+HIIT**		−0.11 (−0.49,0.27)		
0.21 (−0.21, 0.63)	**ER+AE**	**−0.37 (−0.58,−0.15)**	**−0.63 (−0.78,−0.48)**	
−0.11 (−0.48, 0.26)	**−0.32 (−0.52**, **−0.12)**	**ER**		
−0.42 (−0.85, 0.00)	**−0.63 (−0.78**, **−0.48)**	**−0.31 (−0.52**, **−0.11)**	**CON**	
**CRP**				
**ER+COE**			0.04 (−0.27,0.35)	−0.00 (−0.29,0.29)
0.39 (−0.14, 0.91)	**ER+HIIT**		−0.31 (−0.76,0.14)	
**0.59 (0.12, 1.07)**	0.21 (−0.42, 0.84)	**ER+AE**	−0.30 (−0.96,0.36)	**−0.78 (−1.34,−0.22)**
0.07 (−0.22, 0.36)	−0.31 (−0.75, 0.13)	**−0.52 (−0.97**, **−0.07)**	**ER**	
−0.03 (−0.30, 0.24)	−0.42 (−0.95, 0.11)	**−0.63 (−1.06**, **−0.19)**	−0.11 (−0.41, 0.20)	**CON**
**TNF–α**				
**ER+COE**			−0.08 (−0.46,0.30)	−0.06 (−0.43,0.31)
−0.22 (−0.84, 0.39)	**ER+HIIT**		0.20 (−0.34,0.74)	
**0.46 (0.02, 0.90)**	**0.68 (0.07, 1.29)**	**ER+AE**	−0.45 (−1.04,0.14)	**−0.58 (−0.92,−0.23)**
−0.02 (−0.39, 0.34)	0.20 (−0.32, 0.72)	**−0.48 (−0.85**, **−0.11)**	**ER**	
−0.11 (−0.47, 0.25)	0.11 (−0.45, 0.68)	**−0.57 (−0.87**, **−0.27)**	−0.09 (−0.40, 0.22)	**CON**
**IL−6**				
**ER+COE**			0.05 (−0.22,0.33)	−0.21 (−0.49,0.07)
0.36 (−0.25, 0.97)	**ER+HIIT**		−0.28 (−0.71,0.16)	
**0.44 (0.02, 0.85)**	0.08 (−0.50, 0.66)	**ER+AE**	**−0.38 (−0.60,−0.16)**	**−0.95 (−1.18,−0.71)**
0.09 (−0.26, 0.43)	−0.27 (−0.77, 0.23)	**−0.35 (−0.64**, **−0.06)**	**ER**	
−0.34 (−0.71, 0.03)	**−0.70 (−1.27**, **−0.12)**	**−0.77 (−1.05**, **−0.50)**	**−0.43 (−0.71**, **−0.14)**	**CON**
**Adiponectin**				
**ER+COE**			0.35 (−0.24,0.95)	**0.92 (0.42,1.43)**
**0.88 (0.15, 1.60)**	**ER+HIIT**		−0.32 (−0.91,0.28)	
**0.58 (0.07, 1.09)**	−0.30 (−0.93, 0.34)	**ER+AE**	0.14 (−0.26,0.55)	0.14 (−0.16,0.44)
**0.56 (0.07, 1.05)**	−0.31 (−0.85, 0.22)	−0.02 (−0.36, 0.32)	**ER**	
**0.79 (0.33, 1.25)**	−0.09 (−0.72, 0.55)	0.21 (−0.07, 0.49)	0.23 (−0.11, 0.57)	**CON**
**HOMA–IR**				
**ER+COE**			−0.31 (−0.71,0.09)	**−0.98 (−1.43,−0.53)**
0.07 (−0.67, 0.82)	**ER+HIIT**		−0.35 (−0.88,0.18)	
−0.05 (−0.54, 0.43)	−0.13 (−0.83, 0.57)	**ER+AE**	−0.17 (−0.51,0.16)	**−1.04(−1.25,−0.83)**
−0.28 (−0.70, 0.15)	−0.35 (−0.96, 0.26)	−0.22 (−0.57, 0.12)	**ER**	
**−0.93 (−1.38**, **−0.49)**	**−1.01 (−1.72**, **−0.29)**	**−0.88 (−1.20**, **−0.56)**	**−0.66 (−1.02**, **−0.29)**	**CON**

The upper-right triangle displays direct pairwise estimates, whereas the lower-left triangle displays random-effects network meta-analysis estimates. Cells show SMDs with 95% CIs for the column-defining intervention vs. the row-defining intervention. Statistically significant estimates, defined as 95% CIs not crossing 0, are highlighted in bold. For hs-CRP, CRP, TNF-α, IL-6, and HOMA-IR, negative values favor the column-defining intervention; for adiponectin, positive values favor the column-defining intervention. CON, control/usual care; ER, energy restriction; ER+AE, ER plus aerobic exercise; ER+HIIT, ER plus high-intensity interval training; ER+COE, ER plus combined exercise.

The strength of supporting evidence differed across outcomes. The networks for hs-CRP, CRP, and IL-6 included 10, 9, and 14 trials, respectively, whereas the networks for TNF-α, adiponectin, and HOMA-IR included 9, 7, and 11 trials (Supplementary Table 6). Across outcomes, the ER and ER+AE contributed the largest share of direct evidence, while ER+HIIT and ER+COE were supported by fewer head-to-head data (Supplementary Figure 2). Accordingly, estimates for hs-CRP/CRP and IL-6, particularly those involving ER+AE, were generally more stable than estimates for TNF-α, adiponectin, and HOMA-IR, which were based on smaller or less connected networks. Observed between-study heterogeneity estimates were generally low. Global and local inconsistency tests did not provide statistical evidence of inconsistency; however, because several outcome networks were small or sparsely connected, the power to detect inconsistency was limited. Therefore, findings from data-sparse nodes were interpreted cautiously (Supplementary Figure 3; Supplementary Table 6).

#### 3.6.1 Primary outcomes

For CRP and hs-CRP, ER+AE yielded significant reductions in CRP relative to ER alone (SMD −0.52, 95% CI −0.97 to −0.07), alongside comparable favorable effects vs. the control group (SMD −0.63, 95% CI −1.06 to −0.19). For hs-CRP, ER+AE exerted modest beneficial effects when compared with ER alone (SMD −0.32, 95% CI −0.52 to −0.12). Relative to the control group, ER+AE exerted the most prominent and consistent reductions in hs-CRP (SMD −0.63, 95% CI −0.78 to −0.48). None of the remaining treatment regimens yielded statistically noteworthy decreases in hs-CRP or CRP levels.

For TNF-α, only the ER+AE regimen induced statistically significant reductions in this inflammatory marker. Relative to ER alone, the pooled effect estimate for TNF-α was (SMD −0.48, 95% CI −0.85 to −0.11). Comparisons vs. the control group revealed a similar lowering effect (SMD −0.57, 95% CI −0.87 to −0.27). The prediction intervals spanned a broad range across all interventions, which can be attributed to the limited count of eligible trials as well as remaining heterogeneity across the included studies.

For IL-6, comparisons against ER alone showed that only ER+AE induced a further significant reduction in IL-6 (SMD −0.35, 95% CI −0.64 to −0.06), whereas ER+HIIT and ER+COE exhibited no statistically meaningful differences relative to ER alone. When compared with the control group, ER+AE (SMD −0.77, 95% CI −1.05 to −0.50) and ER+HIIT (SMD −0.70, 95% CI −1.27 to −0.12) yielded significant IL-6-lowering effects.

#### 3.6.2 Secondary outcomes

For adiponectin, only ER+COE was associated with a significant increase compared with ER alone (SMD 0.56, 95% CI 0.07 to 1.05), whereas ER+AE and ER+HIIT did not differ significantly from ER alone. When matched against the control group, solely ER+COE produced a significant increase in adiponectin levels (SMD 0.79, 95% CI 0.33 to 1.25); ER+AE and ER+HIIT failed to reach statistical significance for altering adiponectin concentrations.

For HOMA-IR, comparisons against ER alone indicated that none of the combined exercise regimens yielded statistically distinct HOMA-IR reductions compared with ER alone. Compared with control, ER alone and all three exercise-combined ER interventions significantly reduced HOMA-IR: ER+AE (SMD −0.88, 95% CI −1.20 to −0.56), ER+HIIT (SMD −1.01, 95% CI −1.72 to −0.29), and ER+COE (SMD −0.93, 95% CI −1.38 to −0.49), with ER+HIIT showing the largest reduction.

### 3.7 Exploratory treatment hierarchy

Before interpreting treatment hierarchy, we summarized the outcome-specific network geometry, available direct comparisons, and direct/indirect evidence contribution for key ER-referenced comparisons (Table 2). Observed heterogeneity was low to low-to-moderate across outcomes. Global and local tests did not provide statistical evidence of direct–indirect inconsistency. Statistical power to detect inconsistency was constrained, as many networks featured small sample sizes and sparse connections. Network connectivity also varied markedly across different outcome endpoints. ER+AE was generally supported by more comparisons than ER+HIIT and ER+COE, and ER+COE was not represented in the hs-CRP network. In addition, several key comparisons, particularly ER+HIIT vs. ER, were primarily or entirely informed by direct evidence from a limited number of studies. Therefore, subsequent hierarchy estimates were interpreted as exploratory and were not used as the primary basis for clinical recommendations.

**Figure 6:**
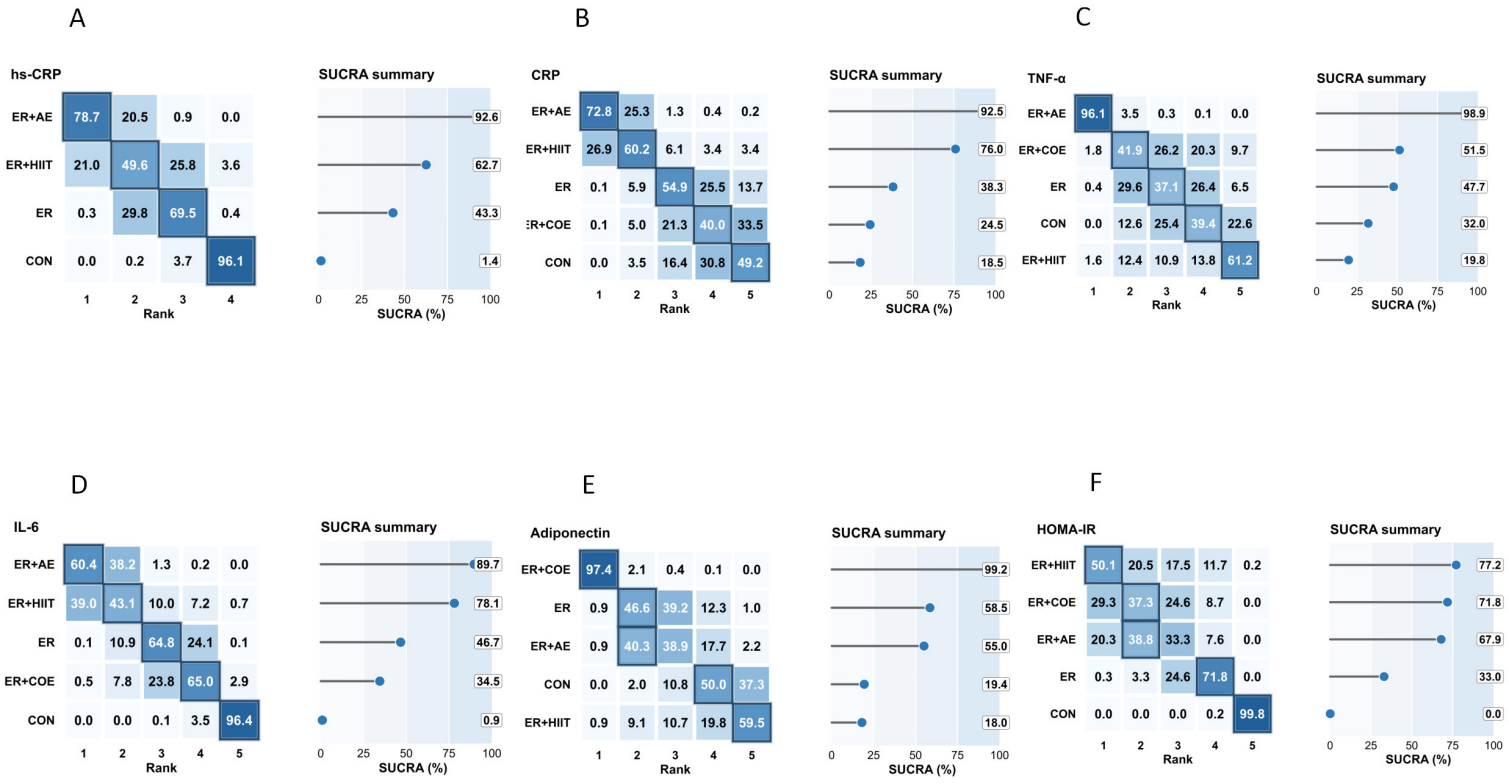
Treatment hierarchy of exercise modalities combined with energy-restricted diets. **(A)** hs-CRP. **(B)** CRP. **(C**) TNF-α. **(D**) IL-6. **(E)** Adiponectin. **(F)** HOMA-IR. Note that this hierarchy is exploratory and should not serve as a primary basis for clinical recommendations.

Exploratory treatment hierarchy analyses suggested potential differences across exercise modalities when combined with ER. The exploratory hierarchy results are presented in Figure 6 and Table 5. Overall, the exploratory hierarchy was broadly consistent with the effect estimates for several outcomes. ER+AE tended to have higher SUCRA values for hs-CRP/CRP and TNF-α, and ER+AE or ER+HIIT had higher exploratory values for IL-6. For secondary outcomes, ER+HIIT or ER+COE had higher exploratory values for HOMA-IR, and ER+COE had a higher value for adiponectin. These patterns should be interpreted as hypothesis-generating because several of the corresponding networks were small or supported by limited direct evidence. These findings cannot be taken as definitive proof of treatment superiority or serve as the core foundation for clinical guidance. This caveat applies especially to TNF-α, adiponectin and HOMA-IR, whose corresponding networks were smaller, sparser and more susceptible to the influence of single trials. Therefore, the hierarchy results were interpreted together with effect estimates, 95% CIs, prediction intervals, direct and indirect evidence contribution, and certainty of evidence.

**Table 5:** Exploratory treatment hierarchy of exercise modalities combined with energy restriction.

Treatment	SUCRA	Treatment	SUCRA	Treatment	SUCRA
hs-CRP		CRP		TNF-α	
ER+AE	92.6	ER+AE	92.5	ER+AE	98.9
ER	62.7	ER+HIIT	76.0	ER+HIIT	51.5
ER+HIIT	43.3	ER	38.3	ER	47.7
CON	1.4	ER+COE	24.5	ER+COE	32.0
		CON	18.5	CON	19.8
**IL-6**		**Adiponectin**		**HOMA-IR**	
ER+AE	89.7	ER+COE	99.2	ER+HIIT	77.2
ER+HIIT	78.1	ER	58.5	ER+COE	71.8
ER	46.7	ER+AE	55.0	ER+AE	67.9
ER+COE	34.5	ER+HIIT	19.4	ER	33.0
CON	0.9	CON	18.0	CON	0.0

CON, control/usual care; ER, energy restriction; ER+AE, ER plus aerobic exercise; ER+HIIT, ER plus high-intensity interval training; ER+COE, ER plus combined exercise. Ranking metrics are exploratory and should not be interpreted as definitive evidence of treatment superiority. These metrics should be considered together with effect estimates, 95% CIs, prediction intervals, formal direct/indirect evidence contribution, and certainty of evidence.

### 3.8 Heterogeneity, inconsistency, and small-study effects

Between-study heterogeneity estimates are reported in Supplementary Table 6. Outcome-specific random-effects models showed generally low-to-moderate observed heterogeneity, and I^2^ values falling within the low-to-moderate range. Design-by-treatment interaction tests did not provide statistical evidence of global inconsistency across the assessed biomarkers, including hs-CRP, CRP, TNF-α, IL-6, adiponectin, and HOMA-IR. Where side-splitting analyses were feasible, local comparisons also did not indicate substantial inconsistency between direct and indirect evidence for most available contrasts. However, because several outcome-specific networks contained sparse data and limited direct evidence, the power to detect inconsistency was limited. These findings should therefore be interpreted as no statistical evidence of detected inconsistency rather than as proof that inconsistency was absent, particularly for smaller networks involving TNF-α, adiponectin, and HOMA-IR (64–66, 86). Comparison-adjusted funnel plots did not show obvious asymmetry suggestive of important small-study effects, although interpretation was limited by the small number of studies in several outcome-specific networks (Supplementary Figure 4).

### 3.9 Population and intervention based sensitivity analyses

To further examine population heterogeneity and generalizability, additional study-level sensitivity analyses were conducted according to age category, metabolic phenotype, and intervention duration (Supplementary Table 7). Across the predefined sensitivity analyses, the direction of the main ER-referenced estimates was generally preserved, particularly for ER+AE on inflammatory outcomes. However, precision decreased after dataset restriction, and some ER+HIIT and ER+COE comparisons became non-estimable or less stable because of sparse or disconnected networks. Therefore, the sensitivity analyses supported the overall robustness of the main inflammatory findings, while indicating that node-specific estimates based on limited evidence should be interpreted cautiously. After excluding trials enrolling minors, ER+AE remained associated with lower hs-CRP and IL-6 compared with ER alone, whereas the corresponding estimates for CRP and TNF-α remained directionally favorable but were no longer statistically significant. After excluding trials specifically enrolling participants with metabolic syndrome, ER+AE remained associated with lower CRP, TNF-α, and IL-6, and ER+COE remained associated with higher adiponectin. Analyses restricted by intervention duration showed broadly similar directions for ER+AE on inflammatory outcomes, but several HIIT and COE comparisons became non-estimable because of sparse or disconnected networks. Across these population- and duration-based sensitivity analyses, none of the exercise-combined ER interventions showed a statistically consistent additional benefit over ER alone for HOMA-IR. These findings indicate key inflammatory outcomes were not predominantly influenced by pediatric trials, participants with metabolic syndrome, or brief interventions. These restricted sensitivity analyses remain exploratory, however, as they relied on study-level stratification and frequently lacked sufficient statistical power.

### 3.10 Exploratory effect modification

Because several ER-referenced outcome networks were too sparse to support stable meta-regression, CON was used as a unified reference comparator for these exploratory analyses. These supplementary meta-regression models, which assessed the impact of study-level covariates on treatment effect estimates, were not used to draw the primary conclusions of this analysis. This regression specifically evaluated effect modification related to mean participant age and intervention duration and acted only as a secondary adjunct to the main network meta-analysis (Supplementary Figure 5). For hs-CRP, ER+AE showed a significant age-related association, suggesting greater reductions in studies with older participants (β = −0.010 per year, *p* = 0.007). The duration association for ER+AE was directionally similar but not statistically significant (β = −0.039 per month, *p* = 0.073). For IL-6, age and duration were not significant modifiers, although ER+AE showed a nonsignificant age-related trend toward greater benefit. For HOMA-IR, age was associated with the ER+AE effect estimate (β = 0.053 per year, *p* = 0.026), but this small-node finding should be interpreted cautiously. Overall, no consistent evidence of effect modification by age or duration was observed, and these analyses should be considered hypothesis-generating.

## 4 Discussion

### 4.1 Main findings and relation to previous evidence

This systematic review and network meta-analysis evaluated the additional benefits of different exercise training modalities when combined with an energy-restricted diet. That design is clinically important because ER itself modifies inflammatory biomarkers, and failure to account for diet can obscure the exercise-specific signal (17, 23, 25, 26, 40, 41). We interpreted exploratory hierarchy metrics together with effect estimates, uncertainty, direct and indirect evidence contribution, and confidence in the evidence, rather than as standalone evidence (58, 64, 67). Overall, when ER alone was used as the primary reference, the incremental benefits of adding exercise appeared to be biomarker specific. ER+AE showed the most consistent additional anti-inflammatory benefit, with favorable effects on hs-CRP/CRP, TNF-α, and IL-6 compared with ER alone. ER+COE produced a significant incremental increase in adiponectin, whereas ER+HIIT and ER+COE did not show clear additional benefits over ER alone for most inflammatory outcomes. For HOMA-IR, no combined exercise plus ER intervention yielded statistically superior improvements relative to ER alone. This implies the beneficial effects against control groups mainly stemmed from ER itself, rather than extra incremental benefits brought by exercise. Taken together, these findings suggest that no single exercise modality is uniformly superior across all outcomes. Instead, the optimal adjunct to ER may depend on the dominant immunometabolic endpoint and should be interpreted in relation to the ER-alone comparator (6–9, 25, 27, 40, 41).

A further key observation from the network analysis was that the robustness of the evidence base varied across outcomes. The hs-CRP/CRP and IL-6 results, particularly those involving ER+AE, were supported by relatively larger and better-connected networks. In contrast, the TNF-α and adiponectin analyses, as well as some HOMA-IR comparisons, were based on smaller networks with fewer direct head-to-head comparisons and wider prediction intervals. Accordingly, although the present findings suggest differential potential benefits of specific exercise modalities when added to ER, they do not establish any single modality as uniformly superior across all clinical contexts. Comparisons vs. control should therefore be regarded as contextual evidence, whereas the primary interpretation should focus on the incremental effects of exercise modalities beyond ER alone (60, 64, 66).

### 4.2 Hs-CRP/CRP and TNF-α

When ER alone was used as the primary comparator, our NMA indicated that ER+AE appeared to yield relatively robust incremental benefits in lowering hs-CRP/CRP and TNF-α across all exercise strategies assessed. CRP is a hepatic acute-phase biomarker that reflects upstream pro-inflammatory signaling and is widely used as an indicator of residual inflammatory risk in cardiovascular disease; in obesity, circulating CRP is strongly related to adiposity, particularly visceral fat and adipose-derived cytokine signaling (14, 24, 87). This pattern is broadly consistent with previous obesity-focused NMAs and supports the possibility that adding AE to ER may provide additional anti-inflammatory benefits, potentially by enhancing reductions in visceral adiposity and attenuating downstream inflammatory spillover from adipose tissue to the liver (24, 32, 40, 41, 88–90).

### 4.3 IL-6

In interpreting IL-6, physiological context is critical. In obesity, elevated resting IL-6 generally reflects chronic low-grade inflammation and metabolic dysregulation, whereas IL-6 released during acute exercise behaves as a myokine involved in substrate mobilization and anti-inflammatory signaling (26, 27, 29, 30, 32). Accordingly, the acute post-exercise rise in IL-6 must be distinguished from chronically elevated resting concentrations, and blood-sampling time points can substantially influence measured responses (29, 32, 91). In this network meta-analysis, with ER alone as the primary comparator, ER+AE appeared to produce relatively strong incremental reductions in circulating IL-6. ER+HIIT also showed favorable IL-6-lowering effects in contextual comparisons vs. control, but its additional benefit beyond ER alone was less certain. Therefore, the observed reductions in circulating IL-6 are most plausibly interpreted as improvements in chronic inflammatory status rather than as acute exercise-induced myokine responses (26, 27, 29, 32, 41). These considerations are particularly important for interpreting IL-6 in a network that includes trials with different intervention durations, participant age groups, and exercise prescriptions. The overall reduction in resting IL-6 should be understood as a general improvement in chronic low-grade inflammation within ER-based interventions, rather than as a simple marker of the transient myokine response to a single exercise session. This distinction also underscores the requirement for standardized blood-sampling windows in upcoming clinical investigations that combine ER with exercise training, particularly when attempting to isolate the incremental contribution of exercise beyond ER alone (26, 29, 32, 91).

### 4.4 HOMA-IR and adiponectin

In the present study, the pattern observed for HOMA-IR differed from that of inflammatory cytokines. Although ER+HIIT and ER+COE appeared to rank more favorably for improving HOMA-IR, none of the exercise-combined ER interventions showed a statistically distinct reduction in HOMA-IR compared with ER alone. Therefore, these ranking results should be interpreted as exploratory rather than as evidence of clear incremental superiority over ER. This discrepancy is physiologically plausible, because insulin resistance is influenced not only by inflammatory status, but also by factors including skeletal muscle glucose metabolism, mitochondrial and metabolic function, and the preservation of fat-free mass throughout energy-restricted body mass reduction (25, 30, 32, 92, 93). HIIT may improve insulin sensitivity through robust metabolic stress and favorable adaptations in oxidative capacity and glucose transport (32, 51, 92, 94). Combined exercise (COE) may confer additional advantages by integrating the muscle-sparing effects of resistance training with the improvements in energy expenditure and cardiopulmonary fitness induced by aerobic training (25, 30, 93). Such a consideration carries special significance during energy restriction, since uncompensated depletion of lean tissue could negate a portion of the advantageous metabolic outcomes (25, 30, 93). As HOMA-IR represents a fasting surrogate index rather than a tissue-resolved mechanistic assay, the proposed biological pathways should be interpreted as plausible mechanistic inferences rather than definitive evidence. Notably, our results suggest that exercise modalities associated with favorable HOMA-IR estimates may differ from those associated with inflammatory-marker reductions; however, this interpretation should be considered hypothesis-generating.

The different exploratory hierarchy patterns across biomarkers do not necessarily indicate a flaw in the dataset. Rather, they suggest that these outcomes capture overlapping but distinct biological processes. CRP/hs-CRP and TNF-α are more closely related to systemic inflammatory status, adiponectin reflects adipose endocrine function, and HOMA-IR is a composite fasting marker of glucose-insulin homeostasis (13, 14, 91, 95). It is therefore biologically plausible that AE, HIIT, and COE show different exploratory hierarchy patterns across biomarkers. However, given the sparse evidence structure and the lack of statistically significant HOMA-IR differences compared with ER alone, these rankings should be viewed as hypothesis-generating rather than as a basis for concluding modality-specific superiority (13, 25–30, 37, 90, 93, 95–98).

### 4.5 Clinical relevance and innovation

Clinically, these findings do not support a single exercise prescription for all patients undergoing ER-based obesity treatment. When ER alone served as the primary comparator, ER+AE demonstrated relatively strong incremental effects in lowering systemic inflammatory markers, particularly hs-CRP/CRP, TNF-α, and IL-6. ER+COE showed a favorable signal for increasing adiponectin, but this interpretation should be tempered by the smaller evidence base. By contrast, although ER+HIIT and ER+COE appeared favorable in exploratory hierarchy analyses for some metabolic outcomes, none of the exercise-combined ER interventions demonstrated a statistically consistent additional benefit over ER alone for HOMA-IR.

The clinical application of these findings should therefore be individualized rather than interpreted as a universal ranking of exercise modalities. The included trials enrolled minors, adults, older adults, and participants with either uncomplicated overweight/obesity or metabolic syndrome, with intervention durations ranging from very short-term to long-term programs. Although sensitivity analyses suggested that the main inflammatory findings were not entirely dependent on one specific population subgroup, several restricted networks became sparse and less precise. Thus, the pooled estimates should be interpreted as average effects across heterogeneous obesity phenotypes rather than as subgroup-specific treatment prescriptions. In practice, adjunctive exercise selection should consider the target biomarker, age, metabolic phenotype, exercise capacity, supervision, safety, and long-term adherence (99, 100).

### 4.6 Advantages, limitations, and prospective research directions

Principal merits of this systematic review include the exclusive use of RCTs, pre-specified and clinically meaningful intervention groupings, explicit assessment of transitivity and inconsistency, and cautious interpretation of treatment hierarchy metrics alongside effect sizes, uncertainty intervals, prediction intervals, and evidence certainty rather than as stand-alone indicators of superiority (43, 47, 56–60, 64, 66, 67).

However, several limitations should be considered. First, the networks for TNF-α, adiponectin, and HOMA-IR were smaller than those for hs-CRP/CRP and IL-6, increasing imprecision and reducing the stability of some estimates, particularly for ER+HIIT and ER+COE. Second, residual clinical heterogeneity remained because trials differed in achieved energy deficit, dietary composition, exercise intensity, supervision, adherence, and biomarker sampling procedures (16, 17, 23, 25, 26, 29, 32, 35, 40, 41, 91). Because the available trials did not consistently report sufficient information for assay- or sampling-based subgroup analyses, residual measurement heterogeneity may have contributed to imprecision. Third, broad aggregation of AE, HIIT, and COE may have masked within-node differences in exercise dose, modality, progression, supervision, and adherence. Finally, non-significant global and local inconsistency tests should not be interpreted as proof of consistency, because the power to detect inconsistency was limited in small or sparsely connected networks. Future research should prioritize sufficiently powered head-to-head trials. Such trials should directly compare exercise modalities vs. ER alone with standardized, quantified ER protocols. They also require unified blood sampling timing, complete adherence and supervision records, and parallel assessments of visceral fat, lean mass, and tissue-specific or exerkine mediators (24, 27, 30, 33, 90, 93, 95).

## 5 Conclusion

When ER alone was used as the primary comparator, the incremental effects of adding exercise were biomarker specific. ER+AE conferred relatively consistent incremental anti-inflammatory effects, particularly for hs-CRP, CRP, TNF-α, and IL-6. ER+COE showed a favorable signal for increasing adiponectin, although this finding was based on a smaller evidence network and should be interpreted cautiously. In contrast, none of the exercise-combined ER interventions demonstrated a statistically consistent additional benefit over ER alone for HOMA-IR across the primary and sensitivity analyses. Overall, adjunctive exercise may be a useful component of ER-based obesity treatment, but the current evidence does not identify a single universally superior exercise modality across biomarkers or patient subgroups. Clinical application should be individualized according to age, metabolic phenotype, exercise capacity, safety, supervision, and long-term adherence.

## Data Availability

The original contributions presented in the study are included in the article/Supplementary material, further inquiries can be directed to the corresponding authors.
