# Supplementary material for: Comparative efficacy of exercise modalities added to energy restriction on inflammatory markers in obesity: a systematic review and network meta-analysis
Source: Front Nutr. 2026 Aug 5;13:1879890. doi: 10.3389/fnut.2026.1879890 (PMC13485891; doi:10.3389/fnut.2026.1879890)
Supplement: Supplementary file 1 [file Supplementary_File_1.docx]

| **Supplemental materials** | Page |
| --- | --- |
| **Supplementary Table 1.** PRISMA NMA checklist of items to include when reporting a systematic review involving a network meta-analysis. | 2-5 |
| **Supplementary Table 2.** Intervention node definitions and decision rules(energy restriction ± exercise). | 6-15 |
| **Supplementary Table 3.** Search strategy of PubMed/MEDLINE, Embase, Web of Science, SPORTDiscus, and the Cochrane Library. | 16-18 |
| **Supplementary Table 4.** Characteristics of Included Studies. | 19-36 |
| **Supplementary Table 5.** Distribution of key effect modifiers across intervention nodes. | 37-44 |
| **Supplementary Figure 1.** Risk of bias of the included study. | 45-52 |
| **Supplementary Figure 2.** Contributions of direct and indirect comparisons to NMA and the number of studies of each direct comparison of hs-CRP, CRP,TNF-α,IL-6,Adiponectin and HOMA-IR. | 53-58 |
| **Supplementary Figure 3.** Forest plots of eligible comparisons of hs-CRP, CRP,TNF-α,IL-6,Adiponectin and HOMA-IR. | 59-64 |
| **Supplementary Table 6.** Heterogeneity diagnostics by outcome (random-effects network meta-analysis). | 65-70 |
| **Supplementary Figure 4.** The funnel plot graphics of hs-CRP, CRP,TNF-α,IL-6,Adiponectin and HOMA-IR in NMA. | 71-76 |
| **Supplementary Table 7.**Sensitivity analysis results of the network meta-analysis | 77-97 |
| **Supplementary Figure 5.** Exploratory study-level meta-regression analyses of mean age and intervention duration modified treatment effects across the network. | 98-109 |

**Supplementary Table 1.** PRISMA NMA checklist of items to include when reporting a systematic review involving a network meta-analysis.

| **Section/Topic** | **Item#** | **Checklist Item** | **Reported on Page #** |
| --- | --- | --- | --- |
| **TITLE** |  |  |  |
| Title | 1 | Identify the report as a systematic review *incorporating a network meta–analysis (or related form of meta–analysis).* | 1 |
| **ABSTRACT** |  |  |  |
| Structurd summary | 2 | Provide a structured summary including, as applicable: **Background:** main objectives  **Methods:** data sources; study eligibility criteria, participants, and interventions; study appraisal; and *synthesis methods, such as network meta–analysis.*  **Results:** number of studies and participants identified; summary estimates with corresponding confidence/credible intervals;  *treatment rankings may also be discussed. Authors may choose to summarize pairwise comparisons against a chosen treatment included in their analyses for brevity.*  **Discussion/Conclusions:** limitations; conclusions and implications of findings.  **Other:** primary source of funding; systematic review registration number with registry name. | 1 |
| **INTRODUCTION** |  |  |  |
| Rationale | 3 | Describe the rationale for the review in the context of what is already known*, including mention of why a network meta–analysis has been conducted.* | 2-3 |
| Objectives | 4 | Provide an explicit statement of questions being addressed, with reference to participants, interventions, comparisons, outcomes, and study design (PICOS). | 2-3 |
| **METHODS** |  |  |  |
| Protocol and  registration | 5 | Indicate whether a review protocol exists and if and where it can be accessed (e.g., Web address); and, if available, provide registration information, including registration number. | 3 |
| Eligibility criteria | 6 | Specify study characteristics (e.g., PICOS, length of follow-up) and report characteristics (e.g., years considered, language, publication status) used as criteria for eligibility, giving rationale. *Clearly describe eligible treatments included in the treatment network, and note whether any have been clustered or merged into the same node (with justification).* | 3 |
| Information sources | 7 | Describe all information sources (e.g., databases with dates of coverage, contact with study authors to identify additional studies) in the search and date last searched. | 4 |
| Search | 8 | Present full electronic search strategy for at least one database, including any limits used, such that it could be repeated. | Supplementary Table 3 |
| Study selection | 9 | State the process for selecting studies (i.e., screening, eligibility, included in systematic review, and, if applicable, included in the meta-analysis). | 5 |
| Data collection process | 10 | Describe method of data extraction from reports (e.g., piloted forms, independently, in duplicate) and any processes for obtaining and confirming data from investigators. | 5 |
| Data items | 11 | List and define all variables for which data were sought (e.g.,  PICOS, funding sources) and any assumptions and simplifications made. | 6-7 |
| **Geometry of the network** | S1 | Describe methods used to explore the geometry of the treatment  network under study and potential biases related to it. This shouldinclude how the evidence base has been graphically summarized for presentation, and what characteristics were compiled and used to  describe the evidence base to readers. | 8 |
| Risk of bias  within individual studies | 12 | Describe methods used for assessing risk of bias of individual  studies (including specification of whether this was done at the  study or outcome level), and how this information is to be used in any data synthesis. | 8 |
| Summary  measures | 13 | State the principal summary measures (e.g., risk ratio, difference in means). *Also describe the use of additional summary measures assessed, such as treatment rankings and surface under the cumulative ranking curve (SUCRA) values, as well as modified approaches used to present summary findings from meta–analyses.* | 8-9 |
| Planned methods of analysis | 14 | Describe the methods of handling data and combining results of studies for each network meta-analysis. This should include, but not be limited to:  • *Handling of multi–arm trials;*  • *Selection of variance structure;*  • *Selection of prior distributions in Bayesian analyses; and*  • *Assessment of model fit.* | 7 |
| **Assessment of Inconsistency** | **S2** | Describe the statistical methods used to evaluate the agreement of direct and indirect evidence in the treatment network(s) studied.Describe efforts taken to address its presence when found. | 7 |
| Risk of bias across studies | 15 | Specify any assessment of risk of bias that may affect the cumulative evidence (e.g., publication bias, selective reporting within studies). | 8 |
| Additional analyses | 16 | Describe methods of additional analyses if done, indicating which were pre-specified. This may include, but not be limited to, thefollowing:  • Sensitivity or subgroup analyses;  • Meta-regression analyses;  • *Alternative formulations of the treatment network; and*  • *Use of alternative prior distributions for Bayesian analyses (if applicable).* | 12-13 |
| **RESULTS**† |  |  |  |
| Study selection | 17 | Give numbers of studies screened, assessed for eligibility, and included in the review, with reasons for exclusions at each stage, ideally with a flow diagram. | 7 |
| **Presentation of**  **network structure** | **S3** | Provide a network graph of the included studies to enable visualization of the geometry of the treatment network. | Figure 2 |
| **Summary of**  **network geometry** | **S4** | Provide a brief overview of characteristics of the treatment network. This may include commentary on the abundance of trials and randomized patients for the different interventions and pairwise comparisons in the network, gaps of evidence in the treatment network, and potential biases reflected by the network structure. | 7-8 |
| Study  characteristics | 18 | For each study, present characteristics for which data were extracted (e.g., study size, PICOS, follow-up period) and provide the citations. | Supplementary Table 4 |
| Risk of bias within studies | 19 | Present data on risk of bias of each study and, if available, any outcome level assessment. | Supplementary Figure 1 |
| Results of  individual studies | 20 | For all outcomes considered (benefits or harms), present, for each study: 1) simple summary data for each intervention group, and 2) effect estimates and confidence intervals. *Modified approaches may* | 10-11 |
| Synthesis of results | 21 | Present results of each meta-analysis done, including confidence/credible intervals. *In larger networks, authors may focus on comparisons versus a particular comparator (e.g., placebo or standard care), with full findings presented in an appendix. League tables and forest plots may be considered to summarize pairwise comparisons.* If additional summary measures were explored (such as treatment rankings), these should also be presented. | 10-11  Figure 3  Table 2 |
| **Exploration for inconsistency** | **S5** | Describe results from investigations of inconsistency. This may include such information as measures of model fit to compare consistency and inconsistency models, *P* values from statistical tests, or summary of inconsistency estimates from different parts of the treatment network. | 12-13  Supplementary Table 5 |
| Risk of bias across studies | 22 | Present results of any assessment of risk of bias across studies for the evidence base being studied. | 11  Supplementary Figure 1 |
| Results of additional analyses | 23 | Give results of additional analyses, if done (e.g., sensitivity or subgroup analyses, meta-regression analyses*, alternative network geometries studied, alternative choice of prior distributions for Bayesian analyses,* and so forth). | 12-13  Supplementary Figure 5 |
| **DISCUSSION** |  |  |  |
| Summary of evidence | 24 | Summarize the main findings, including the strength of evidence for each main outcome; consider their relevance to key groups (e.g.,healthcare providers, users, and policy-makers). | 13 |
| Limitations | 25 | Discuss limitations at study and outcome level (e.g., risk of bias),and at review level (e.g., incomplete retrieval of identified research, reporting bias). *Comment on the validity of the assumptions, such as transitivity and consistency. Comment on any concerns regarding network geometry (e.g., avoidance of certain comparisons).* | 15 |
| Conclusions | 26 | Provide a general interpretation of the results in the context of other evidence, and implications for future research. | 16 |
| **FUNDING** |  |  |  |
| Funding | 27 | Describe sources of funding for the systematic review and other support (e.g., supply of data); role of funders for the systematic review. This should also include information regarding whether funding has been received from manufacturers of treatments in the network and/or whether some of the authors are content experts with professional conflicts of interest that could affect use of treatments in the network. | 16 |

PICOS = population, intervention, comparators, outcomes, study design.

* Text in italics indicate wording specific to reporting of network meta-analyses that has been added to guidance from the PRISMA statement.

**Supplementary Table 2. Intervention node definitions and decision rules (energy restriction + exercise)**

**Supplementary Table 2. Part 1. Exercise modality nodes and minimum operational criteria**

| **Node** | **Operational definition** | **Key implementation details recorded (for transitivity)** | **Reference** |
| --- | --- | --- | --- |
| Usual care / usual lifestyle (Control) | Usual diet and physical activity with no quantified ER prescription and no structured/supervised exercise programme; may include general health education or lifestyle advice. | Any co-interventions; contact time; whether diet or physical-activity advice was provided. | [1-4] |
| Energy restriction only (ER) | Dietary programme meeting strict, quantified ER criteria A-C without a structured exercise programme; habitual physical activity maintained. Active dietary/lifestyle interventions without clear quantified ER were flagged as borderline/non-strict ER for sensitivity analysis rather than treated as strict ER. | Deficit/target (kcal/day or %), diet type, monitoring, counseling frequency, and whether the arm was strict ER or borderline/non-strict ER. | [1-3] |
| ER + Aerobic exercise (ER+AE) | ER diet plus structured aerobic/endurance training not meeting HIIT criteria. Aerobic training prescribed >=2 sessions/week for >=2 weeks; intensity in moderate-to-vigorous domain where reported. | FITT, supervision, intensity prescription/verification, adherence. | [4] |
| ER + High-intensity interval training (ER+HIIT) | ER diet plus interval-based training explicitly described as HIIT/HIT or clearly interval-structured, with repeated work bouts performed in the severe-intensity domain, typically >=85% HRmax or >=80-90% VO2peak/peak power, interspersed with recovery; prescribed >=2 sessions/week for >=2 weeks. | Interval structure, intensity targets and verification, supervision, adherence, adverse events. | [6,7] |
| ER + Combined training (ER+COE) | ER diet plus an intervention intentionally integrating both aerobic/endurance and resistance/strength training within the same programme. Resistance component required a structured training prescription targeting major muscle groups; aerobic component meeting AE criteria; programme duration >=2 weeks. | Resistance prescription, aerobic prescription, sequencing, supervision, adherence. | [5,8] |

**Supplementary Table 2. Part 2. Hierarchy for classifying the dietary platform**

Dietary arms were first assessed against strict, quantified ER criteria A-C. Arms satisfying at least one of these criteria were considered strict ER for the main classification. When an active dietary or lifestyle intervention was reported but the published article did not clearly quantify the energy prescription, the arm was flagged as borderline/non-strict ER for transparency and sensitivity testing. Such arms were not automatically reallocated to the passive comparator node because they involved active dietary counselling, food provision, macronutrient restriction, or structured weight-management education and were therefore not clinically equivalent to no dietary intervention. To evaluate robustness, all borderline/non-strict ER cases were excluded in the strict-ER sensitivity analysis.

A.Explicit energy deficit: stated energy deficit relative to requirements (kcal/day) or percent deficit (e.g., 15-40% below estimated energy requirements. [1,2]

B. Explicit energy intake target: fixed energy prescription (kcal/day) or energy-per-kg target with weight-loss intent and monitoring (e.g., meal plans specifying total daily energy. [1,2]

C. Quantified hypoenergy diet protocol: low-/very-low-energy diet, meal-replacement formula, intermittent fasting protocol, or structured menus with stated energy content enabling inference of sustained negative energy balance. [1,2]

Additional analytic flag: borderline/non-strict ER = active dietary/lifestyle weight-management intervention without a clearly quantified energy deficit, fixed kcal target, or stated-energy hypoenergy menu in the published report. These cases were retained only in the broad primary ER-based network and excluded in strict-ER sensitivity analyses.

**References**

1. Jensen MD, Ryan DH, Apovian CM, Ard JD, Comuzzie AG, Donato KA, et al. 2013 AHA/ACC/TOS guideline for the management of overweight and obesity in adults. Circulation. 2014;129(25 Suppl 2):S102-S138. doi:10.1161/01.cir.0000437739.71477.ee.

2. Garvey WT, Mechanick JI, Brett EM, Garber AJ, Hurley DL, Jastreboff AM, et al. American Association of Clinical Endocrinologists and American College of Endocrinology comprehensive clinical practice guidelines for medical care of patients with obesity. Endocr Pract. 2016;22(Suppl 3):1-203. doi:10.4158/EP161365.GL.

3. Yumuk V, Tsigos C, Fried M, Schindler K, Busetto L, Micic D, et al. European guidelines for obesity management in adults. Obes Facts. 2015;8(6):402-424. doi:10.1159/000442721.

4. Garber CE, Blissmer B, Deschenes MR, Franklin BA, LaMonte MJ, Lee IM, et al. American College of Sports Medicine position stand. Quantity and quality of exercise for developing and maintaining cardiorespiratory, musculoskeletal, and neuromotor fitness. Med Sci Sports Exerc. 2011;43(7):1334-1359. doi:10.1249/MSS.0b013e318213fefb.

5. American College of Sports Medicine. Progression models in resistance training for healthy adults. Med Sci Sports Exerc. 2009;41(3):687-708. doi:10.1249/MSS.0b013e3181915670.

6. Buchheit M, Laursen PB. High-intensity interval training, solutions to the programming puzzle. Part II. Sports Med. 2013;43(10):927-954. doi:10.1007/s40279-013-0066-5.

7. Coates AM, Joyner MJ, Little JP, Jones AM, Gibala MJ. A Perspective on High-Intensity Interval Training for Performance and Health. Sports Med. 2023;53(Suppl 1):85-96. doi:10.1007/s40279-023-01938-6.

8. Schumann M, Feuerbacher JF, Sünkeler M, Freitag N, Rønnestad BR, Doma K, et al. Compatibility of Concurrent Aerobic and Strength Training for Skeletal Muscle Size and Function. Sports Med. 2022;52(3):601-612. doi:10.1007/s40279-021-01587-7.=38664

**Supplementary Table 2, Part 3. Study-level classification of ER criteria**

Strict ER criteria: explicit energy deficit, explicit energy-intake target, or quantified hypoenergy protocol. Borderline/non-strict ER: active dietary intervention without a clearly quantified energy prescription in the published report. Sensitivity rule: studies/arms meeting strict ER criteria were retained in the strict-ER sensitivity analysis. Borderline/non-strict ER cases were retained in the broad primary ER-based network and excluded from the strict-ER sensitivity analysis.Classification summary: 24 studies; 18 retained as strict ER; 6 flagged as borderline/non-strict ER and excluded in the strict-ER sensitivity analysis.

| **Study** | **Dietary prescription reported in the study** | **ER classification** | **Classification rationale** | **Monitoring / adherence information** | **Strict-ER sensitivity decision** |
| --- | --- | --- | --- | --- | --- |
| Gyorkos 2019 | Carbohydrate-restricted Paleolithic-based diet; CHO <50 g/day; macronutrient goals approximately 25% protein, 60% fat, and 15% carbohydrate. No explicit total caloric intake instruction was provided. Reported energy intake decreased spontaneously during the intervention. | Borderline/non-strict ER | Structured macronutrient-restricted dietary intervention with weight-management intent, but the article did not report a quantified energy-deficit target, fixed kcal prescription, or stated-energy hypocaloric menu. It was therefore flagged as borderline/non-strict rather than strict quantified ER. | Self-monitoring app, 24-hour dietary recalls, NDSR nutrient analysis, beta-hydroxybutyrate checks; diet goals reportedly achieved. | Included in the broad primary ER-based network with a borderline/non-strict flag; excluded from the strict-ER sensitivity analysis. |
| Nicklas 2004 | Dietary weight-loss intervention intended to produce and maintain approximately 5% baseline weight loss. Participants were initially counseled to decrease energy intake by 500 kcal/day, targeting about 0.5 kg/week weight loss. | A | Meets strict quantified ER because an explicit daily energy deficit of 500 kcal/day was prescribed. | Registered-dietitian sessions; weekly body-weight checks during the intensive phase; attendance and monthly weight assessments used for adherence. | Retained in primary and strict-ER sensitivity analyses. |
| Reljic 2022 | All groups received comprehensive one-on-one nutritional counseling. Participants were advised to achieve a daily caloric deficit of 500 kcal and to maintain protein intake of >=1.0 g/kg/day. | A | Meets strict quantified ER because an explicit 500 kcal/day energy deficit was prescribed. | Handouts with recipes and food lists; 3-day food records at study onset and final intervention week; dietary analysis with PRODI software. | Retained in primary and strict-ER sensitivity analyses. |
| Villareal 2006 | Balanced low-calorie diet prescribed to provide an energy deficit of approximately 750 kcal/day. Daily energy requirement was estimated from resting energy expenditure multiplied by 1.3; goal was 10% weight loss by study completion. | A | Meets strict quantified ER because an explicit daily energy deficit of approximately 750 kcal/day was prescribed. | Weekly group meetings with a study dietitian; behavioral strategies including goal setting and self-monitoring. | Retained in primary and strict-ER sensitivity analyses. |
| Weiss 2016 | Interventions were designed to create a 20% energy deficit and 6-8% weight loss. CR reduced energy intake by approximately 20%; CREX created the same total deficit with CR and exercise each contributing about half. | A | Meets strict quantified ER because a percentage energy deficit was prescribed, including a defined dietary CR component in CREX. | 3-day food diaries, weekly clinic weights, daily home weight logs, and prescription adjustment to achieve targeted weight loss; body-weight stabilization before follow-up testing. | Retained in primary and strict-ER sensitivity analyses. |
| Heiston 2021 | Thirteen-day low-calorie diet. Participants were instructed to consume 1000-1200 kcal/day; meal-replacement shakes were provided for breakfast and lunch, with menus for low-kcal snacks and dinners not exceeding 600 kcal. | B/C | Meets strict quantified ER because a fixed kcal target and structured low-calorie/meal-replacement protocol were specified. | Diet adherence reported; meal replacements and menus supported adherence; interval-exercise adherence reported separately for LCD+INT. | Retained in primary and strict-ER sensitivity analyses. |
| Oh 2013 | Low-calorie dietary intervention with intake monitored to remain <1500 kcal/day; carbohydrate intake was reported as 55-60% of calories, and dietary instruction emphasized reducing high-glycemic foods. | B | Meets strict quantified ER because a fixed caloric ceiling (<1500 kcal/day) was stated. | Daily food diary; individual monitoring/counseling based on food diaries and health monitoring at intervention sessions. | Retained in primary and strict-ER sensitivity analyses. |
| Sartor 2010 | Two-week moderately low-carbohydrate, high-unsaturated-fat diet. Calorie intake was reduced to 75% of daily energy expenditure; macronutrient distribution was approximately 35% carbohydrate, 50% fat, and 15% protein. Food was provided by research staff. | A | Meets strict quantified ER because an explicit 25% energy restriction relative to daily energy expenditure was prescribed. | 7-day diet diary before intervention; provided foods during intervention; participants reported deviations and diets were adjusted to preserve average diet values. | Retained in primary and strict-ER sensitivity analyses. |
| Schweitzer 2023 | diet prescribed 10-15% fat, 15-20% protein, and 65-75% carbohydrates, while restricting salt to <1500 mg/day, cholesterol to <100 mg/day, and providing approximately 40g of fiber per 1000 kcal. The diet specified macronutrient composition and food restrictions, but the article did not report a fixed kcal target or prescribed energy deficit. | Borderline/non-strict ER | Active food-provision and dietary-quality intervention with weight-management intent, but the published report did not clearly state a quantified energy deficit or fixed caloric target. It was therefore treated as borderline/non-strict for sensitivity testing. | All meals shipped weekly; nutrition/behavioral sessions and cooking classes; receipt confirmed by mail tracking; intervention attendance reported. | Included in the broad primary ER-based network with a borderline/non-strict flag; excluded from the strict-ER sensitivity analysis unless additional trial records document a quantified kcal prescription. |
| Imayama 2012 | Twelve-month caloric restriction weight-loss diet based on Diabetes Prevention Program / Look AHEAD. Goals: 1200-2000 kcal/day based on weight, <=30% calories from fat, 10% weight loss within the first 24 weeks, then maintenance. | B | Meets strict quantified ER because a fixed daily caloric intake target range was stated and tied to weight-loss intent. | Dietitians delivered behavior-modification sessions; 2-4 individual sessions, weekly group sessions to week 24, monthly sessions thereafter, plus phone/e-mail contacts; daily diet logs for first 6 months. | Retained in primary and strict-ER sensitivity analyses. |
| Nunes 2015 | energy intake set at Dietary Reference Intake levels for low physical activity.A 3-day dietary record was made for each adolescent.Weekly nutritional education on topics such as food pyramid, food records, weight-loss diets, diet/light concepts, fat/cholesterol, and eating disorders. Energy intake was set at dietary-reference levels for low physical activity by age and sex; no explicit kcal deficit or hypocaloric target was reported. | Borderline/non-strict ER | Structured nutrition education and reference-based dietary guidance were reported, but the article did not clearly prescribe a sustained quantified energy deficit or fixed hypocaloric target. It was therefore flagged as borderline/non-strict. | 3-day dietary records completed with help from parents; Dietpro software used for macronutrient evaluation. | Included in the broad primary ER-based network with a borderline/non-strict flag; excluded from the strict-ER sensitivity analysis. |
| Plavsic 2020 | Standard, well-balanced, low-fat, hypocaloric diet of 1500-1700 kcal/day for 3 months. Participants were instructed to reduce caloric intake by 300-500 kcal/day; written weekly menu examples were provided. | A/B | Meets strict quantified ER because both a fixed kcal target range and explicit 300-500 kcal/day reduction were specified. | Four to six individual nutrition sessions with a pediatrician; 4-day dietary recalls before and after intervention reviewed by a dietitian and analyzed with Nutrisoft. | Retained in primary and strict-ER sensitivity analyses. |
| Bouchonville 2014 | Balanced diet prescribed to provide a 500-750 kcal/day deficit from daily energy requirement and approximately 1 g/kg body weight high-quality protein. Goal: 10% weight loss at 6 months and weight maintenance thereafter. | A | Meets strict quantified ER because an explicit 500-750 kcal/day deficit was prescribed. | Weekly visits with a dietitian; standard behavioral strategies to modify eating habits; compliance data reported in parent trial. | Retained in primary and strict-ER sensitivity analyses. |
| Lefevre 2009 | Six-month intervention. CR prescribed 25% calorie restriction from baseline energy requirements; ER+EX prescribed 12.5% calorie restriction plus 12.5% increased energy expenditure through structured aerobic exercise. Diets were based on AHA guidelines. | A | Meets strict quantified ER because percentage calorie restriction was prescribed and individualized from measured baseline energy requirements. | Energy prescription based on doubly labeled water and body-weight change during kitchen-fed run-in; metabolic kitchen provided foods during weeks 1-12 and 23-24; weekly behavioral meetings. | Retained in primary and strict-ER sensitivity analyses. |
| Lakhdar 2013 | Balanced personalized dietary restriction program set at 500 kcal/day below initial dietary records; approximately 15% protein, 55% carbohydrate, and 30% lipid. Diet designed according to individual dietary habits. | A | Meets strict quantified ER because an explicit 500 kcal/day deficit below baseline intake was prescribed. | Food/fluid intake recorded before program; specifically designed notebooks used during intervention; diet calculated with Bilnut software; body mass measured weekly. | Retained in primary and strict-ER sensitivity analyses. |
| Keawtep 2024 | Three-month intermittent fasting protocol: self-selected intake at 25-75% of estimated energy requirements for 2 days/week (75% weeks 1-4, 50% weeks 5-8, 25% weeks 9-12), with ad libitum intake on the remaining 5 days. | A/C | Meets strict quantified ER as a quantified hypocaloric protocol because the fasting-day prescription was expressed as a percentage of estimated energy requirements. Restriction was intermittent rather than daily. | Mobile-phone food records; caloric intake calculated using Thai nutritive values; participants trained before intervention; weekly contact encouraged adherence. | Retained in primary and strict-ER sensitivity analyses; intermittent-fasting design noted in transitivity assessment. |
| Bo 2007 | Intervention group received individualized verbal and written recommendations. First session provided an individually prescribed written normo- or hypocaloric diet tailored to body weight and reported intake; recommended distribution: 50-60% carbohydrate, 15-20% protein, <30% fat, <10% saturated fat, up to 10% polyunsaturated fat, and 20-30 g fiber. | Borderline/non-strict ER | Individualized dietary counseling and possible hypocaloric prescription were described, but the report did not provide a quantified energy deficit or fixed kcal target. It was therefore flagged as borderline/non-strict rather than strict quantified ER. | Validated food-frequency questionnaire and Minnesota Leisure-Time Physical Activity questionnaire before and after; 5 professional sessions including diet/exercise/behavior counseling. | Included in the broad primary ER-based network with a borderline/non-strict flag; excluded from the strict-ER sensitivity analysis unless study records provide subject-level kcal targets. |
| Christiansen 2010 | Twelve-week protocol: DIO and DEX received liquid very-low-energy diet for 8 weeks followed by 4-week weight-maintenance diet. DIO received 600 kcal/day and DEX 800 kcal/day to account for exercise energy expenditure; maintenance diet was based on estimated energy requirements. | B/C | Meets strict quantified ER because explicit fixed kcal targets and VLED protocol were stated. | Subjects randomized to DIO/DEX; weight loss maintained through subsequent 4-week maintenance phase. | Retained in primary and strict-ER sensitivity analyses. |
| Tang 2021 | Eight-week calorie-restriction protocol. Daily energy intake was controlled at 100-110% of each participant's measured basal energy expenditure. Participants gradually reduced intake to the target during the first 2 weeks and maintained the target range during the following 6 weeks; dietary advice also included a diverse diet, avoiding/reducing sugar-sweetened beverages, and a 30/40/30 distribution for breakfast/lunch/dinner. | B | Meets strict quantified ER because the dietary prescription provided an individualized quantitative daily energy-intake target based on measured BEE. It was classified as an intake-target criterion because the deficit relative to total daily energy expenditure was not explicitly stated. | Daily food-intake reporting through a self-designed mobile application; app alerts and WeChat contact for compliance; 25/26 participants completing CR provided dietary records at baseline, week 4, and week 8. | Retained in primary and strict-ER sensitivity analyses; relatively mild/intake-target-based CR noted in transitivity assessment. |
| Makni 2010 (Ben Ounis et al.) | Eight-week dietary-restriction program combined with training. Energy requirements were estimated from age and habitual activity. The diet/training group diet was set at 500 kcal/day below initial dietary records; macronutrient composition was approximately 15% protein, 55% carbohydrate, and 30% lipid, with high-fiber grains, vegetables, and fruits emphasized. | A | Meets strict quantified ER because an explicit daily energy deficit of 500 kcal/day below baseline dietary records was prescribed. | One-week pre-program dietary records; nutrient intake calculated with Bilnut software and Tunisian food-composition tables; community education program delivered 4 h/week. The control group was instructed to maintain usual eating behaviors. | Retained in primary and strict-ER sensitivity analyses. |
| Nemet 2013 | Three-month multidisciplinary childhood-obesity intervention. Participants received a balanced hypocaloric diet: 1200-2000 kcal/day depending on age and weight, or about 30% below reported intake, or 15% below estimated daily required intake. | A/B | Meets strict quantified ER because both a fixed kcal range and explicit percentage energy-reduction options were reported. | Six dietitian meetings; first appointment used a 24-hour dietary recall and assessed food choices, dietary/cooking habits, and family motivation; subsequent meetings provided nutritional education with worksheets/flyers. | Retained in primary and strict-ER sensitivity analyses. |
| Pena 2023 | Six-month lifestyle intervention for Latino youth with obesity and prediabetes. Nutrition/health education promoted a healthy balanced diet, reducing saturated fat, added sugars, and sugar-sweetened beverages, managing portion sizes, and increasing fiber, fruit, and vegetable intake. No quantified energy deficit, fixed kcal target, or hypocaloric menu was reported. | Borderline/non-strict ER | Structured nutrition/health education with weight-management intent was reported, but a sustained caloric deficit or energy-intake target was not specified. It was therefore flagged as borderline/non-strict. | Weekly 75-min nutrition/health education with behavior-change skills training in the intervention group; usual care involved two meetings with a pediatric endocrinologist and registered dietitian to discuss healthy lifestyle SMART goals. | Included in the broad primary ER-based network with a borderline/non-strict flag; excluded from the strict-ER sensitivity analysis. |
| Shalitin 2009 | Twelve-week diet-only or diet-plus-exercise program. D and D+E participants received a balanced hypocaloric diet of 1200 kcal/day with 25-30% fat, 10-15% protein, and 55-65% carbohydrate. At the end of the intervention, participants received counseling to maintain a balanced 1500 kcal/day diet during follow-up. | B | Meets strict quantified ER because a fixed daily caloric target of 1200 kcal/day was prescribed during the intervention. | Twelve weekly 60-min group meetings with a dietitian; food records completed by children and parents and reviewed/discussed by the study dietitian, although detailed statistical analysis of each record was not performed. | Retained in primary and strict-ER sensitivity analyses. |
| Vos 2011 | Three-month family-based cognitive-behavioral multidisciplinary lifestyle intervention. Nutritional advice was based on a 3-day dietary recall and traffic-light nutrition list; children and parents planned their daily diet using healthy-eating principles. The paper reported treatment goals for weight reduction, but no explicit kcal deficit, fixed kcal target, or quantified hypocaloric protocol was provided. | Borderline/non-strict ER | Family-based dietary counseling and weight-management goals were reported, but the dietary prescription was not quantitatively energy-restricted according to the strict criteria. It was therefore flagged as borderline/non-strict. | Individual screening/counseling by a dietitian, physiotherapist, psychologist, and social worker; 7 child group meetings, 5 parent meetings, one joint parent-child meeting, and refresher sessions over 2 years. | Included in the broad primary ER-based network with a borderline/non-strict flag; excluded from the strict-ER sensitivity analysis. |

Abbreviations: ER, energy restriction; AE, aerobic exercise; HIIT, high-intensity interval training; COE, combined exercise; VLED, very-low-energy diet; IF, intermittent fasting; BEE, basal energy expenditure.

**Supplementary Table 3.** Search strategy of PubMed/MEDLINE, Embase, Web of Science, SPORTDiscus, and the Cochrane Library.

| **Database** | **Dietary Energy Restriction / Weight Loss** | **Exercise** | **Inflammatory Biomarkers** | **Limits / Filters** |
| --- | --- | --- | --- | --- |
| PubMed MEDLINE | "Caloric Restriction"[Mesh]; "Diet, Reducing"[Mesh]; "Diet, High-Protein Low-Carbohydrate"[Mesh]; "Diet"[Mesh]  Free-text terms: caloric restricted; energy restricted diet*; low-energy diet*; low energy diet; reducing diet*; weight loss diet*; weight reduction diet*;diet, high protein low carbohydrate; diets, high-protein low-carbohydrate;high-protein carbohydrate-restricted diet*; high protein carbohydrate restricted diet*; carbohydrate-restricted high-protein diet*; carbohydrate restricted high protein diet*; low carbohydrate high protein diet; Atkins Diet; South Beach Diet;Broad diet capture: diet*; Japanese Diet; New Nordic Diet. | "Exercise"[Mesh]; "Resistance Training"[Mesh]; "High-Intensity Interval Training"[Mesh]; "Racquet Sports"[Mesh]; "Sports"[Mesh]  Free-text terms: physical exercise*; physical activit*; aerobic exercise*; isometric exercise*; acute exercise*; exercise training*;resistance training*; strength training; weight-lifting strengthening program*; weight-lifting exercise program*; weight-bearing strengthening program*; weight-bearing exercise program*;high intensity interval training*; high-intensity intermittent exercise*; sprint interval training*; racquet sport*; racket sport*; badminton; lacrosse; racquetball; racketball; racket ball; squash; sport*; athletic*. | "Inflammation"[Mesh] OR inflammation*[tiab] OR innate inflammatory response*[tiab]; "Interleukins"[Mesh] OR interleukin*[tiab]; "Interleukin-6"[Mesh]; "Interleukin-8"[Mesh]; "Interleukin-10"[Mesh]; "Interleukin 1 Receptor Antagonist Protein"[Mesh]; "C-Reactive Protein"[Mesh]; "Tumor Necrosis Factor-alpha"[Mesh]; "Interleukin-18"[Mesh]; "Transforming Growth Factor beta2"[Mesh]; "Leptin"[Mesh]; "Adiponectin"[Mesh]; "Insulin Resistance"[Mesh].  Free-text terms: IL-6/IL6; IL-8/IL8/CXCL8; IL-10/IL10; IL-1Ra/anakinra; CRP/hs-CRP; TNF-α/TNFalpha/cachectin; IL-18/IL18; TGF-beta2/TGF-beta-2; leptin-related ob-gene terms; adiponectin-related ACRP30/apM-1 terms; and insulin resistance/HOMA-IR terminology. | Jan 1, 2000-Jan 10, 2026  Humans  RCT filter: randomized controlled trial[pt] OR randomized[tiab] OR placebo[tiab]  No language restriction |
| Embase (Elsevier) | Emtree terms: 'caloric restriction'/exp OR 'caloric restriction'; 'low energy diet'/exp OR 'low energy diet'; 'high-protein low-carbohydrate diet'/exp OR 'high-protein low-carbohydrate diet'; 'diet'/exp OR 'diet'  Free-text terms (:ab,ti): caloric restricted; energy-restricted/low-energy diet*; diet, reducing; reducing/weight-loss diet*; diet*; Japanese diet; New Nordic diet; high-protein low-carbohydrate/carbohydrate-restricted diet variants; Atkins diet; South Beach diet. | Emtree terms: 'exercise'/exp OR 'exercise'; 'resistance training'/exp OR 'resistance training'; 'high intensity interval training'/exp OR 'high intensity interval training'; 'racquet sport'/exp OR 'racquet sport'; 'sport'/exp OR 'sport'  Free-text terms (:ab,ti): physical exercise/activity*; aerobic/isometric/acute exercise*; exercise training*; resistance/strength training; weight-lifting or weight-bearing strengthening/exercise program*; high intensity interval training/high-intensity intermittent exercise/sprint interval training*; racquet/racket sport*; badminton; lacrosse; racquetball/racketball/racket ball; squash (sport); sport*; athletic*. | Emtree terms: 'inflammation'/exp OR 'inflammation'; 'cytokine'/exp OR 'cytokine'; 'interleukin 6'/exp OR 'interleukin 6'; 'interleukin 8'/exp OR 'interleukin 8'; 'interleukin 10'/exp OR 'interleukin 10'; 'interleukin 1 receptor blocking agent'/exp OR 'interleukin 1 receptor blocking agent'; 'c reactive protein'/exp OR 'c reactive protein'; 'tumor necrosis factor'/exp OR 'tumor necrosis factor'; 'interleukin 18'/exp OR 'interleukin 18'; 'transforming growth factor beta2'/exp OR 'transforming growth factor beta2'; 'leptin'/exp OR 'leptin'; 'adiponectin'/exp OR 'adiponectin'; 'insulin resistance'/exp OR 'insulin resistance'  Free-text terms (:ab,ti): inflammation*; innate inflammatory response*; interleukin*; cytokine*; IL-6/IL6 and historical aliases; IL-8/IL8/CXCL8 and chemotactic-factor aliases; IL-10/IL10; IL-1Ra/anakinra; c reactive protein/hs-CRP/hs-CRP; TNF-α/TNFalpha/cachectin; IL-18/IL18/IFN-gamma-inducing factor aliases; TGF-beta2/TGF-beta-2/TGF-beta2LAP; ob gene product; ACRP30/apM-1; insulin sensitivity/HOMA-IR/homeostatic model assessment terms. | Publication year: [2000-2026]/py  Study filter: 'randomized controlled trial':ab,ti OR 'randomized':ab,ti OR 'placebo':ab,ti  Field restriction: :ab,ti for free-text terms; /exp for exploded Emtree terms |
| Web of Science Core Collection (Topic search, TS=) | TS=("Mediterranean diet*" OR "caloric restriction" OR "low-energy diet*" OR "weight loss diet*" OR "high-protein low-carbohydrate" OR "Atkins diet" OR "South Beach diet" OR diet* OR "Japanese diet" OR "New Nordic diet") | TS=(exercise OR "physical activit*" OR "aerobic exercise*" OR "resistance training" OR "strength training" OR "high-intensity interval training" OR "sprint interval training*" OR "racquet sport*" OR sport* OR athletic*) | TS=(inflammation OR interleukin* OR cytokine* OR "interleukin-6" OR "interleukin-8" OR "interleukin-10" OR "interleukin-18" OR "interleukin 1 receptor antagonist protein" OR "C-reactive protein" OR "tumor necrosis factor-alpha" OR "transforming growth factor beta2" OR leptin OR adiponectin OR "insulin resistance") | Timespan: 2000-01-01 to 2026-01-10 Study design: TS=("randomized controlled trial" OR randomized OR placebo) |
| SPORTDiscus with Full Text (EBSCOhost) | Key terms extracted under SU: caloric restriction; energy restricted diet*; low-energy diet*; diet, reducing; reducing diet*; weight loss diet*; weight reduction diet*; high-protein low-carbohydrate diet; Atkins Diet; South Beach Diet; diet*; Japanese Diet; New Nordic Diet. | Key terms extracted under SU: exercise; physical exercise*; physical activit*; aerobic exercise*; isometric exercise*; acute exercise*; exercise training*; resistance training*; strength training; high-intensity interval training; sprint interval training*; racquet sports; badminton; lacrosse; racquetball; squash; sport*; athletic*. | Key terms extracted under SU: inflammation; interleukin*; IL-6; IL-8/CXCL8; IL-10; IL-1 receptor antagonist / IL-1Ra / anakinra; C-reactive protein / hs-CRP; TNF-α / cachectin; IL-18; transforming growth factor beta2; leptin; adiponectin; insulin resistance / HOMA-IR. | Study design filter: SU(randomized controlled trial OR randomized OR placebo). Additional interface limits recorded in the log: peer-reviewed; publication date 20000101-20260110 |
| Cochrane Library Trials | MeSH descriptors: [Caloric Restriction], [Diet, Reducing], [Diet, High-Protein Low-Carbohydrate], and [Diet] (all exploded).  Free-text terms: caloric restricted; energy restricted diet*; low-energy diet*; low energy diet; reducing diet*; weight loss diet*; weight reduction diet*; diet, high protein low carbohydrate; diets, high-protein low-carbohydrate; carbohydrate-restricted/high-protein diet variants; low carbohydrate high protein diet; Atkins Diet; South Beach Diet; diet*; Japanese Diet; New Nordic Diet. | MeSH descriptors: [Exercise], [Resistance Training], [High-Intensity Interval Training], [Racquet Sports], and [Sports] (all exploded).  Free-text terms: physical exercise*; physical activit*; aerobic exercise*; isometric exercise*; acute exercise*; exercise training*; resistance training*; strength training; weight-lifting/weight-bearing program variants; high intensity interval training*; high-intensity intermittent exercise*; sprint interval training*; racquet/racket sport*; badminton; lacrosse; racquetball; racketball; racket ball; squash (sport); sport*; athletic*. | MeSH descriptors: General and specific biomarker descriptors: [Inflammation], [Interleukins], [Interleukin-6], [Interleukin-8], [Interleukin-10], [Interleukin 1 Receptor Antagonist Protein], [C-Reactive Protein], [Tumor Necrosis Factor-alpha], [Interleukin-18], [Transforming Growth Factor beta2], [Leptin], [Adiponectin], and [Insulin Resistance] (all exploded).  Free-text terms: inflammation*; innate inflammatory response*; interleukin*; IL-6/IL6; IL-8/IL8/CXCL8; IL-10/IL10; IL-1Ra/anakinra; C reactive protein/hs-CRP; TNF-α/TNFalpha/cachectin; IL-18/IL18; TGF-beta2/TGF-beta-2; ob-gene product terms; ACRP30/apM1 terms; insulin resistance/HOMA-IR terminology. | Free-text fields restricted to ti, ab, kw  Publication Year: 2000-2026  Source subset: Trials |

**Supplementary Table 4.** Characteristics of Included Studies.

**Supplementary Table 4.part 1.** Characteristics of the included studies.

| **Study** | **Country** | **Sample size** | | **Age, years** | | **Participants** | **Duration** | **Intervention type** | **Diet / energy restriction** | **Exercise** | **Outcomes** |
| --- | --- | --- | --- | --- | --- | --- | --- | --- | --- | --- | --- |
|  |  | **Con/**  **inter** | **Female/male** | **con** | **Inter** |  |  |  |  |  |  |
| Gyorkos 2019 | USA/Canada | 5/7 | 8/4 | 40.9±20.2 | | Subjects with Metabolic  Syndrome | 4 weeks | ER+HIIT | Carbohydrate-restricted Paleolithic-based diet; Subjects were instructed to reduce  carbohydrate (CHO) consumption to <50g/d. | intervention consisted of 3 supervised sessions per week on a cycle ergometer. Each 26-minute session included 10 intervals of 60-second sprinting at ~90% HRmax, each followed by 60 seconds of active recovery. | hs-CRP,  IL-6,  TNF-α, HOMA-IR, |
| Nicklas 2004 | USA | 60/53 | 78/35 | 69±6 | 68±7 | Older overweight and obese, sedentary men and women | 18 months | ER+COE | Energy intake reduced by ~500 kcal/day; goal ~5% weight loss. | .intervention involved 60-minute sessions, 3 days/week. The sessions comprised 30 minutes of walking at 50-75% heart rate reserve and 15 minutes of resistance training (2 sets of 12 reps for leg extension, curl, heel raise, and step up using cuff weights/vests). | CRP,  IL-6,  TNF-α |
| Reljic 2022 | Germany | 26/26 | 28/24 | 49±15.1 | 50.6±11.3 | Adults with obesity, metabolic syndrome and a sedentary lifestyle | 12 weeks | ER+HIIT | intervention required patients to achieve a daily caloric deficit of 500 kcal while maintaining a protein intake of ≥1.0 g/kg/day. Nutritional intake was assessed using 3-day food records and specialized software. | intervention involved two 14-minute weekly sessions on cycle ergometers. Each session comprised 5 intervals of 1 minute at 80-95% HRpeak (intensity progressively increased every 4 weeks), interspersed with 1-minute recovery periods. | hs-CRP,  CRP,  IL-6 |
| Villareal 2006 | USA | 10/17 | 9/18 | 71±4 | 69±5 | Obese older adults. | 6 months | ER+AE | intervention prescribed a daily energy deficit of 750 kcal, with a diet composition of 30% fat, 50% carbohydrate, and 20% protein, aiming to limit weekly weight loss to ≤1.5%.. | 90-minute group sessions, 3 days per week, comprising endurance exercise (≥70% VO₂peak) and flexibility exercises | CRP,  IL-6,  HOMA-IR |
| Weiss 2016 | USA | 17/19 | 28/8 | 57±5 | 57±7 | Sedentary, middle-aged men and postmenopausal women aged  45–65 y who were overweight | 12 weeks | ER+AE | intervention aimed to reduce energy intake by 20%, guided by personalized advice from 3-day food diaries. Macronutrient targets were set at 45–65% carbohydrate, 20–35% fat, and 10–35% protein. For some participants, a full provision of a 20% hypocaloric diet was used. | intervention aimed to increase total energy expenditure by 20%. Participants used Polar HR monitors for tracking and were encouraged toward daily moderate-to-high intensity cardiovascular exercise (e.g., walking, cycling), explicitly excluding strength training. Initial 3-6 sessions were supervised. | CRP |
| Heiston 2021 | USA | 12/13 | 25/0 | 45.6±3.5 | 48.5±3.8 | Females with obesity | 2 weeks | ER+HIIT | intervention prescribed a diet of 1000–1200 kcal/day, utilizing 160-kcal meal replacement shakes for breakfast and lunch, with dinners not exceeding 600 kcal. | Intervention consisted of 12 supervised, 60-minute sessions comprising alternating 3-minute intervals at 50% and 90% HRpeak, with a 350-kcal shake consumed post-exercise. | CRP,  TNF-α |
| Oh 2013 | Korea | 21/31 | 52/0 | 66.9±9 | 59.8±7.9 | Korean women, 40 years of age, with metabolic syndrome. | 6 months | ER+AE | Intervention aimed for an intake of <1500 kcal/day with carbohydrates limited to 55–60% of total energys, primarily by reducing high-glycemic foods. Participants maintained daily food diaries for monitoring. | intervention included supervised group sessions (3/week for months 1-3, then 2/week), each 40 minutes long and expending approximately 200 kcal. Participants were also encouraged to perform daily brisk walking with pedometers and maintain an exercise diary.. | hs-CRP,  TNF-α, Adiponectin, |
| Sartor 2010 | UK (Wales) | 9/10 | 14/5 | 37±10 | 41±14 | Obese adults | 2 weeks | ER+HIIT | a 2-week hypocaloric diet providing 75% of daily energy needs, with a macronutrient composition of 35% carbohydrates (150–200 g/day), 50% fat (33% unsaturated, 17% saturated), and 15% protein. | intervention consisted of supervised HIIT sessions performed 3 times/week for 2 weeks, comprising up to ten 4-minute cycling bouts at 90% VO₂peak interspersed with 2–3 minutes of rest, following a 1-week familiarization period. | IL-6,  TNF-α, |
| Schweitzer 2023 | USA | 26/28 | 44/10 | 55±7 | 54±9 | overweight or obese adults | 6 weeks | ER+COE | diet prescribed 10-15% fat, 15-20% protein, and 65-75% carbohydrates, while restricting salt to <1500 mg/day, cholesterol to <100 mg/day, and providing approximately 40g of fiber per 1000 kcal. | exercise intervention included 18 supervised 1-hour sessions (3 sessions/week) involving endurance exercise (targeting 70-85% of maximal heart rate), strength training, and flexibility exercises. | CRP |
| Imayama 2012 | USA | 87/116 | 203/0 | 57.4±4.4 | 58±4.4 | Overweight/obese postmenopausal women. | 12 months | ER+AE | intervention aimed for a daily intake of 1,200-2,000 kcal with ≤30% energys from fat and a 10% weight loss goal within 24 weeks. It was delivered by dietitians via individual sessions followed by weekly (to week 24). | intervention prescribed 225 minutes of moderate-to-vigorous activity weekly, achieved through 3 supervised facility and 2 home sessions. Participants progressively increased to 45-minute sessions at 70-85% of maximal heart rate, using equipment like treadmills and stationary bikes. | hs-CRP, IL-6 |
| Nunes 2015 | Brazil | 8/17 | 13/12 | 15.4±1.2 | 16.18±1.51 | Obese adolescents | 6 months | ER+COE | energy intake set at Dietary Reference Intake levels for low physical activity.A 3-day dietary record was made for each adolescent. | intervention combined twice-weekly aerobic and resistance training. Aerobic sessions on a treadmill cycled through low-intensity (60% VO₂max), moderate, and high-intensity interval protocols. Resistance training used non-linear periodization across five exercises, with intensity structured in three repetition zones (2-6 RM, 8-12 RM, 14-18 RM) and loads progressing by 10%. | CRP, Adiponectin |
| Plavsic 2020 | Serbia | 22/22 | 44/0 | 15.8±1.5 | 16.6±1.3 | Adolescent girls with obesity | 12 weeks | ER+HIIT | intervention prescribed a daily intake of 1500–1700 kcal, aiming for a deficit of 300–500 kcal, with macronutrient distribution of 50–55% carbohydrates, 25–30% lipids, and 20% protein. It included 4–6 individual nutrition counseling sessions (20–30 min each) | intervention was performed 2 days per week for 43 minutes per session. The protocol consisted of a 10-minute warm-up, followed by 4 intervals of 4 minutes at 85-90% HRmax with 3-minute active recovery periods at 70% HRmax, and concluded with a 5-minute cool-down. Adherence was monitored with Polar HR monitors, and a 90% session completion rate was required. | hs-CRP, HOMA-IR |
| Bouchonville 2014 | Mexico | 27/28 | 34/21 | 69±4 | 70±4 | Obese older adults | 12 months | ER+AE | intervention prescribed a daily deficit of 500-750 kcal, 1 g/kg protein intake, and aimed for a 10% weight loss at 6 months followed by maintenance | intervention consisted of 30-minute sessions, conducted three times per week in a supervised facility setting. Exercise modes included treadmill, stationary cycling, and stair climbing, with intensity progressively increased from an initial 65% to 70-85% of peak heart rate | hs-CRP, Adiponectin, HOMA-IR |
| Lefevre 2009 | USA | 11/12 | 13/10 | 38±8 | 36±6 | overweight adult. | 24 weeks | ER+AE | intervention included a 25% reduction from baseline energy requirements (CR group) and a combined approach of 12.5% caloric restriction with 12.5% increased expenditure (ER+EX group). The diet composition was fixed at 30% fat, 15% protein, and 55% carbohydrate. For the first 12 and final 2 weeks. | intervention was performed 5 days per week. Participants self-selected exercise intensity (47-76% VO₂max), with individual energy expenditure targets set at 403±63 kcal/session (women, ~53±11 min) and 569±118 kcal/session (men, ~45±14 min)., aiming for a 12.5% increase in total energy expenditure. | Hs-CRP, |
| Lakhdar 2013 | Tunisia | 10/10 | 20/0 | 38.9±3.94 | 38.9±4.37 | Obese women | 24 weeks | ER+AE | intervention prescribed a daily deficit of 500 kcal with a macronutrient composition of 15% protein, 55% carbohydrates, and 30% lipids. Participants recorded their intake four times weekly in a designated notebook. | intervention consisted of three weekly treadmill walking/running sessions. The protocol was progressive, starting at 55% of maximal heart rate for 30 minutes during weeks 1-4, and gradually increasing to 80% of maximal heart rate for 45 minutes by weeks 20-24. | IL-6,  TNF-α, Adiponectin |
| Keawtep 2024 | Thailand | 23/23 | 46/0 | 53.61±2.81 | 52.17±3..35 | Postmenopausal women with obesity | 3 months | ER+COE | dietary intake with 25–75%  of their estimated energy requirements for 2 days/week  (75% of energy requirements for weeks 1–4, 50% of  energy requirements for weeks 5–8, and 25% of energy  requirements for weeks 9–12) | intervention involved 60-minute sessions (10-min warm-up, 40-min core exercise, 10-min cool-down) conducted 3 days per week. The core exercise combined aerobic dance (e.g., high knee marching) with resistance training for major muscle groups . | IL-6, Adiponectin, HOMA-IR |
| Bo 2007 | Italy | 166/169 | 195/140 | 55.7±5.7 | 55.7±5.6 | Adults with metabolic syndrome | 12 months | ER+AE | intervention provided a personalized hypocaloric diet prescription, recommending a daily energy distribution of 50-60% from carbohydrates, 15-20% from protein, <30% from fat (<10% saturated fat), and 20-30g of fiber | intervention provided personalized advice, recommending at least 150 minutes per week of moderate-intensity activity, such as brisk walking, in line with established guidelines.. | CRP,  HOMA-IR |
| Christiansen 2010 | Denmark | 19/21 | 20/20 | 35.6±7 | 37.5±8 | Obese older adults | 12 weeks | ER+AE | a very low energy liquid diet of 600 kcal/day and 800 kcal/day, respectively, for 8 weeks. This was followed by a 4-week weight maintenance phase. | supervised aerobic exercise 3 times per week, with each session lasting 60-75 minutes and designed to expend 500-600 kcal. | IL-6, Adiponectin, HOMA-IR |
| Tang 2021 | China | 14/12 | 16/10 | 19.4±0.6 | 19.4±0.7 | Young adults with overweight/obesity | 8 weeks | ER+AE | intervention prescribed an energy intake of 100-110% of measured basal expenditure over 8 weeks. It advised a diverse diet avoiding sugary drinks, with daily energys split 30%/40%/30% | intervention prescribed individualized rope skipping exercise 3 times per week, with each 90-minute session structured as 20 minutes of skipping followed by 10 minutes of rest, repeated 3 times. The target intensity was 1,500 skips per session, maintained after a 2-week adaptation period. | CRP, IL-6, TNF-α, HOMA-IR |
| Makni 2010 | France | 14/14 | 14/14 | 13.3 ± 0.6 | 13.1±0.8 | Obese children | 8 weeks | ER+AE | intervention prescribed a daily deficit of 500 kcal with a macronutrient composition of 15% protein, 55% carbohydrates, and 30% fat. Key dietary restrictions included cholesterol intake ≤100 mg/day and sodium ≤1600 mg/day. The education component was delivered for 4 hours per week.. | intervention involved children in 90-minute sessions, 4 times per week. Activities included running, jumping, and ball play, with exercise intensity individually fixed at the heart rate corresponding to each participant's Lipox max. | CRP,  IL-6,  TNF-α |
| Nemet 2013 | Israel | 20/21 | 21/20 | 10.49 ± 2.67 | 10.41±1.96 | Children with obesity | 3 months | ER+COE | intervention for children prescribed an individualized daily intake of 1,200–2,000 kcal or a deficit of approximately 30%, based on age and weight. | intervention for children involved twice-weekly, 1-hour group sessions primarily consisting of endurance games (50% team sports, 50% running games). Additionally, participants were instructed to perform an extra 30–45 minutes of weight-bearing sport activities at least once a week. | IL-6,  HOMA-IR |
| Peña 2023 | USA | 24/40 | 25/39 | 13.2 ± 1.4 | 13.4±1.4 | Latino adolescents with obesity | 6 months | ER+AE | reducing saturated  fat intake, added sugars, and sugar-sweetened beverages, managing portion sizes, and  increasing intake of fiber, fruit, and vegetables. | intervention included 2 weekly 60-min YMCA sessions (circuit/cardiovascular training, sports) with ≥150 bpm target heart rate, plus 1 monitored weekly session to reach 180 mins/week of moderate-to-vigorous aerobic activity.t. | IL-6,  TNF-α |
| Shalitin 2009 | Israel | 27/28 | 26/29 | 8.51 ± 1.52 | 8.2±1.56 | Children with obesity | 12 weeks | ER+COE | hypocaloric diet of 1,200 kcal/day (25–30% fat, 10–15% protein, 55–65% carbohydrates). | intervention involved 90-minute supervised sessions, conducted 3 days per week. Each session was equally divided, with 45 minutes dedicated to aerobic exercise (team sports and running games) and 45 minutes dedicated to resistance training (including exercises such as sit-ups, hand-lifting of small weights, and ball exercises). | CRP,  IL-6,  HOMA-IR |
| Vos 2011 | Netherlands | 33/36 | 32/37 | 13.1± 1.9 | 13.3±2.0 | Children with obesity | 3 months | ER+AE | The Dutch dietary guidelines align with caloric restriction standards via colour-coded low-energy-dense, nutrient-rich food prioritisation and controlled high-energy intake, ensuring adequate nutrition with reduced total energy. | increasing aerobic activities like walking/cycling and reducing sedentary behavior to optimize daily aerobic exercise engagement. | Hs-CRP, Adiponectin |

**Supplementary Table 4.part 2.** List of included studies

1.Gyorkos A, Baker MH,et al. Carbohydrate-restricted Diet and High-intensity Interval Training Exercise Improve Cardio-metabolic and Inflammatory Profiles in Metabolic Syndrome: A Randomized Crossover Trial. Cureus. 2019;11(9):e5596.

2.Barbara J Nicklas,et al. Diet-induced weight loss, exercise, and chronic inflammation in older, obese adults: a randomized controlled clinical trial. The American Journal of Clinical Nutrition. 2004;79(3):544-551.

3.Reljic D, Dieterich W, Herrmann HJ, Neurath MF, Zopf Y. “HIIT the Inflammation”: Comparative Effects of Low-Volume Interval Training and Resistance Exercises on Inflammatory Indices in Obese Metabolic Syndrome Patients Undergoing Caloric Restriction. Nutrients. 2022;14(10):1996. doi:10.3390/nu14101996

4.Villareal DT, Miller BV 3rd, Banks M, Fontana L, Sinacore DR, Klein S. Effect of lifestyle intervention on metabolic coronary heart disease risk factors in obese older adults. Am J Clin Nutr. 2006;84(6):1317-1323.

5.Weiss EP, Albert SG, Reeds DN, Kress KS, McDaniel JL, Klein S, Villareal DT. Effects of matched weight loss from energy restriction, exercise, or both on cardiovascular disease risk factors: a randomized intervention trial. Am J Clin Nutr. 2016;104(3):576-586.

6.Heiston EM, Gilbertson NM, Eichner NZM, Malin SK. A Low-energy Diet with or without Exercise Reduces Postprandial Aortic Waveform in Females with Obesity. Med Sci Sports Exerc. 2021;53(4):796-803.

7.Oh EG, Bang SY, Kim SH, Hyun SS, Chu SH, Jeon JY, Im JA, Lee JE, Lee MK. Therapeutic Lifestyle Modification Program Reduces Plasma Levels of the Chemokines CRP and MCP-1 in Subjects With Metabolic Syndrome. Biol Res Nurs. 2013;15(1):48-55.

8.Sartor F, de Morrée HM, Matschke V, Marcora SM, Milousis A, Thom JM, Kubis HP. High-intensity exercise and carbohydrate-reduced energy-restricted diet in obese individuals. Eur J Appl Physiol. 2010;110(5):893-903.

9.Schweitzer GG, Butsch WS, Kincade MR, et al. Short-term Intensive Lifestyle Therapy in a Worksite Setting Improves Cardiometabolic Health in People With Obesity. J Endocr Soc. 2023;7(6):bvad048.

10.Imayama I, Ulrich CM, Alfano CM, et al. Effects of a Caloric Restriction Weight Loss Diet and Exercise on Inflammatory Biomarkers in Overweight/Obese Postmenopausal Women: A Randomized Controlled Trial. Cancer Res. 2012;72(9):13.

11.Nunes JED, Cunha HS, Freitas ZR, et al. Interdisciplinary therapy changes superoxide dismutase activity and adiponectin in obese adolescents: a randomised controlled trial. J Sports Sci. 2016;34(10):945-950.

12.Fillon A, Beaulieu K, Miguet M, et al. Effects of high-intensity interval training and nutrition advice on cardiometabolic markers and aerobic fitness in adolescent girls with obesity. Appl Physiol Nutr Metab. 2020;45(3):294-300.

13.Villareal DT, Chode S, Parimi N, et al. Weight Loss, Exercise, or Both and Cardiometabolic Risk Factors in Obese Older Adults: Results of a Randomized Controlled Trial. Int J Obes (Lond). 2014;38(3):423-431.

14.Lefevre M, Redman LM, Heilbronn LK, et al. Caloric Restriction Alone and with Exercise Improves CVD Risk in Healthy Non-obese Individuals. Atherosclerosis. 2009;203(1):206-213.

15.Lakhdar N, Denguezli M, Zaouali M, et al. Diet and Diet Combined with Chronic Aerobic Exercise Decreases Body Fat Mass and Alters Plasma and Adipose Tissue Inflammatory Markers in Obese Women. Inflammation. 2013;36(6).

16.Keawtep P, Sungkarat S, Boripuntakul S, et al. Effects of combined dietary intervention and physical-cognitive exercise on cognitive function and cardiometabolic health of postmenopausal women with obesity: a randomized controlled trial. Int J Behav Nutr Phys Act. 2024;21(1):28.

17.Esposito K, Pontillo A, Di Palo C, et al. Effectiveness of a Lifestyle Intervention on Metabolic Syndrome. A Randomized Controlled Trial. J Gen Intern Med. 2007;22(12):1695-1703.

18.Christiansen T, Paulsen SK, Bruun JM, et al. Exercise training versus diet-induced weight-loss on metabolic risk factors and inflammatory markers in obese subjects: a 12-week randomized intervention study. Am J Physiol Endocrinol Metab. 2010;299(4):E752-E760.

19.Tang Z, Ming Y, Wu M, et al. Effects of Caloric Restriction and Rope-Skipping Exercise on Cardiometabolic Health: A Pilot Randomized Controlled Trial in Young Adults. Nutrients. 2021;13(9):3322.

20.Makni E, Makni E, Elloumi M, et al. Effect of individualized exercise training combined with diet restriction on inflammatory markers and IGF-1/IGFBP-3 in obese children. Ann Nutr Metab. 2010;56(4):260-266.

21.Nemet D, Oren S, Pantanowitz M, et al. Effects of a Multidisciplinary Childhood Obesity Treatment Intervention on Adipocytokines, Inflammatory and Growth Mediators. Horm Res Paediatr. 2013;79(6):325-332.

22.Peña A, Olson ML, Ayers SL, et al. Inflammatory Mediators and Type 2 Diabetes Risk Factors before and in Response to Lifestyle Intervention among Latino Adolescents with Obesity. Nutrients. 2023;15(11):2442

23.Nagelberg N, Phillip M, Shtaif B, et al. Effects of a Twelve-Week Randomized Intervention of Exercise and/or Diet on Weight Loss and Weight Maintenance, and Other Metabolic Parameters in Obese Preadolescent Children. Horm Res Paediatr. 2009.

24.Vos RC, Wit JM, Pijl H, et al. Long-term effect of lifestyle intervention on adiposity, metabolic parameters, inflammation and physical fitness in obese children: a randomized controlled trial. Nutr Diabetes. 2011.

**Supplementary Table 4.part 3.** List of included studies Study-specific conversion table

| Study | Outcome(s) | Provided data type | Conversion formula |
| --- | --- | --- | --- |
| Weiss 2016 | CRP | mean ± SE | SD = SE × sqrt(n). |
| Heiston 2021 | CRP, TNF-α | Pre-intervention mean and change values reported as mean ± SE | SD = SE × sqrt(n). SD_post = sqrt(SD_pre^2 + SD_change^2 − 2 × r × SD_pre × SD_change), r = 0.5. |
| Oh 2013 | hs-CRP, TNF-α, adiponectin | mean ± SE | SD = SE × sqrt(n). |
| Imayama 2012 | hs-CRP, IL-6 | mean with 95% CI | SD = sqrt(n) × (Upper − Lower) / 3.92. |
| Bouchonville 2014 | hs-CRP, adiponectin, HOMA-IR | Pre-intervention values and change values reported as mean ± SD | SD_post = sqrt(SD_pre^2 + SD_change^2 − 2 × r × SD_pre × SD_change), r = 0.5. |
| Lefevre 2009 | hs-CRP | Baseline mean of hs-CRP plus log-transformed change, reported with SE | SD_change = SE_change × sqrt(n). mean_endpoint =mean_baseline×e^[−(ln mean_baseline−ln mean_endpoint)].  SD_post = sqrt(SD_pre^2 + SD_change^2 − 2 × r × SD_pre × SD_change), r = 0.5. |
| Tang 2021 | hs-CRP, HOMA-IR | hs-CRP: Mean and median (IQR)). TNF-α and IL-6 were reported as mean ± SD | Approximate SD = (Q3 − Q1) / 1.35. |
| Peña 2023 | TNF-α, IL-6 | mean ± SE | SD = SE × sqrt(n). |
| Shalitin 2009 | CRP, IL-6, HOMA-IR | mean ± SE | SD = SE × sqrt(n). |
| Vos 2011 | hs-CRP, adiponectin | Mean with 95% CI | SE = (Upper − Lower) / (2 × t0.975,df) SD = SE × sqrt(n).  SD = sqrt(n) × (Upper − Lower) / 3.92. |
| Bo 2007 | hs-CRP, HOMA-IR | hs-CRP,HOMA-IR: Mean and median (IQR)). | Approximate SD = (Q3 − Q1) / 1.35. |

**Supplementary Table 4. Part 4.** Population and Intervention Characteristics of Included Studies

| No. | Study | Age group / age profile | Intervention group baseline BMI | Comparator baseline BMI (CON and/or ER / diet-only) | Population category | Metabolic status | Intervention | Duration category | Generalizability note |
| --- | --- | --- | --- | --- | --- | --- | --- | --- | --- |
| 1 | Gyorkos 2019 | Adults; mean age 40.9 ± 20.2 years | 34.9 ± 6.3 | ER: 35.4 ± 4.3 | Adults with metabolic syndrome | Metabolic syndrome / elevated cardiometabolic risk | CR + HIIT | 4 weeks; short-term | Findings mainly apply to adults with metabolic syndrome; very short intervention period. |
| 2 | Nicklas 2004 | Older adults; control 69 ± 6, intervention 68 ± 7 years | 33.9 ± 5.6 | CON:34.5±5.3 ER: 34.4 ± 4.9 | Older overweight/obese sedentary men and women | Overweight/obesity; older sedentary population | CR + COE | 18 months; longer-term | Older adult and long-duration trial; generalizability to younger adults or minors should be cautious. |
| 3 | Reljic 2022 | Adults; control 49 ± 15.1, intervention 50.6 ± 11.3 years | 37.8 ± 6.6 | CON:36.6± 5.2 | Adults with obesity, metabolic syndrome, and sedentary lifestyle | Obesity with metabolic syndrome / elevated cardiometabolic risk | CR + HIIT | 12 weeks; short-term | Applies mainly to obese adults with metabolic syndrome; not directly equivalent to uncomplicated obesity. |
| 4 | Villareal 2006 | Older adults; control 71 ± 4, intervention 69 ± 5 years | 39 ± 5 | CON: 39 ± 5 | Obese older adults | Obesity in older adults | CR + AE | 6 months; longer-term | Older adult population; clinical implications should not be generalized to children or young adults. |
| 5 | Weiss 2016 | Adults; control 57 ± 5, intervention 57 ± 7 years | 28.3 ± 0.4 | ER: 27.7 ± 0.4 | Sedentary middle-aged men and postmenopausal women with overweight | Overweight/obesity; postmenopausal/middle-aged sedentary population | CR + AE | 12 weeks; short-term | Middle-aged/postmenopausal profile may limit generalizability to younger adults and minors. |
| 6 | Heiston 2021 | Adults; control 45.6 ± 3.5, intervention 48.5 ± 3.8 years | 37.3 ± 2.0 | ER: 37.9 ± 1.6 | Females with obesity | Obesity; female-only population | CR + HIIT | 2 weeks; short-term | Very short-term female-only study; reflects early response rather than long-term effects. |
| 7 | Oh 2013 | Adults; control 66.9 ± 9, intervention 59.8 ± 7.9 years | 26.3 ± 3.7 | CON:26.2± 3.7 | Korean women aged 40 years or older with metabolic syndrome | Metabolic syndrome / elevated cardiometabolic risk | CR + AE | 6 months; longer-term | Female metabolic syndrome population; age distribution is adult to older-leaning. |
| 8 | Sartor 2010 | Adults; control 37 ± 10, intervention 41 ± 14 years | 32 ± 4 | ER: 32 ± 3 | Obese adults | Obesity without explicit metabolic syndrome classification | CR + HIIT | 2 weeks; short-term | Very short intervention; useful for early biomarker changes but limited for long-term inference. |
| 9 | Schweitzer 2023 | Adults; control 55 ± 7, intervention 54 ± 9 years | 41.6 ± 8.3 | CON:37.7± 5.4 | Overweight or obese adults | Overweight/obesity without explicit metabolic syndrome classification | CR + COE | 6 weeks; short-term | Short-term adult trial; generalizability to minors and longer-term treatment should be cautious. |
| 10 | Imayama 2012 | Adults; control 57.4 ± 4.4, intervention 58 ± 4.4 years | 31.0 ± 4.3 | CON:30.7±3.9  ER: 31.1±3.9 | Overweight/obese postmenopausal women | Overweight/obesity; postmenopausal female population | CR + AE | 12 months; longer-term | Sex- and menopause-specific population; long-term adherence and maintenance may influence effects. |
| 11 | Nunes 2015 | Minors; control 15.4 ± 1.20, intervention 16.18 ± 1.51 years | 34.99 ± 3.70 | CON:34.85±4.32 | Obese adolescents | Adolescent obesity | CR + COE | 6 months; longer-term | Minor/adolescent population; should not be directly generalized to adults. |
| 12 | Plavsic 2020 | Minors; control 15.8 ± 1.5, intervention 16.6 ± 1.3 years | 32.6 ± 2.7 | ER: 33.2 ± 3.5 | Adolescent girls with obesity | Adolescent obesity; female-only population | CR + HIIT | 12 weeks; short-term | Adolescent girls only; limited generalizability to adult or male populations. |
| 13 | Bouchonville 2014 | Older adults; control 69 ± 4, intervention 70 ± 4 years | 37.2 ± 5.4 | CON: 37.3 ± 4.7 ER: 37.2 ± 4.5 | Obese older adults | Obesity in older adults | CR + AE | 12 months; longer-term | Older-adult long-term trial; should be interpreted separately from child/adolescent trials. |
| 14 | Lefevre 2009 | Adults; control 38 ± 8, intervention 36 ± 6 years | 27.5 ± 1.6 | CON: 27.6 ± 2.0 ER: 27.8 ± 1.4 | Overweight adults | Overweight/obesity without explicit metabolic syndrome classification | CR + AE | 24 weeks; longer-term | Adult overweight population; intermediate-to-long duration. |
| 15 | Lakhdar 2013 | Adults; control 38.9 ± 3.94, intervention 38.9 ± 4.37 years | 32.98 ± 2.17 | ER: 33.02 ± 1.89 | Obese women | Obesity; female-only population | CR + AE | 24 weeks; longer-term | Female-only adult sample; generalizability to men and minors is limited. |
| 16 | Keawtep 2024 | Adults; control 53.61 ± 2.81, intervention 52.17 ± 3.35 years | 29.73 ± 4.57 | CON:29.18± 2.85 ER: 28.28 ± 2.78 | Postmenopausal women with obesity | Obesity; postmenopausal female population | CR + COE | 3 months; short-term | Postmenopausal women only; may not generalize to men, younger women, or minors. |
| 17 | Bo 2007 | Adults; control 55.7 ± 5.7, intervention 55.7 ± 5.6 years | 29.7 ± 4.1 | CON: 29.8 ± 4.6 | Adults with metabolic syndrome | Metabolic syndrome / elevated cardiometabolic risk | CR + AE | 12 months; longer-term | Metabolic syndrome and long-term trial; distinct from uncomplicated obesity. |
| 18 | Christiansen 2010 | Adults; control 35.6 ± 7, intervention 37.5 ± 8 years | 34.2 ± 3 | ER: 35.3 ± 4 | Obese adults | Obesity without explicit metabolic syndrome classification | CR + AE | 12 weeks; short-term | Adult obesity population; extracted age suggests not an older-adult sample despite any broad wording. |
| 19 | Tang 2021 | Adults; control 19.4 ± 0.6, intervention 19.4 ± 0.7 years | 24.5 ± 2.4 | ER: 24.8 (2.5) | Young adults with overweight/obesity | Overweight/obesity; young-adult population | CR + AE | 8 weeks; short-term | Young adult trial; should not be generalized to minors or older adults without caution. |
| 20 | Makni 2010 | Minors; control 13.3 ± 0.6, intervention 13.1 ± 0.8 years | 31.1 ± 1.1 | CON: 30.7 ± 1.6 | Obese children | Childhood obesity | CR + AE | 8 weeks; short-term | Child population; adult clinical recommendations should not rely on this study alone. |
| 21 | Nemet 2013 | Minors; control 10.49 ± 2.67, intervention 10.41 ± 1.96 years | 27.1± 5.31 | CON:26.6± 4.68 | Children with obesity | Childhood obesity | CR + COE | 3 months; short-term | Child population; may reflect pediatric responses and growth-related factors. |
| 22 | Peña 2023 | Minors; control 13.2 ± 1.4, intervention 13.4 ± 1.4 years | 32.1 ± 3.7 | CON: 33.7 ± 6.3 | Latino adolescents with obesity | Adolescent obesity; ethnicity-specific sample | CR + AE | 6 months; longer-term | Adolescent Latino sample; generalizability to adults or other populations should be cautious. |
| 23 | Shalitin 2009 | Minors; control 8.51 ± 1.52, intervention 8.2 ± 1.56 years | 25.9 ± 0.51 | ER: 26.5 ± 0.51 | Children with obesity | Childhood obesity | CR + COE | 12 weeks; short-term | Child population; active-intervention endpoint should be distinguished from later follow-up. |
| 24 | Vos 2011 | Minors; control 13.1 ± 1.9, intervention 13.3 ± 2.0 years | 32.4 ± 4.7 | CON: 32.5 ± 3.9 | Children with obesity | Childhood/adolescent obesity | CR + AE | 3 months; short-term | Pediatric population; adult generalizability is limited. |

**Supplementary Table 4. Part 5.** Include the testing information, institution, fasting status, and blood collection time for outcome indicators.

| No. | Study | Included outcome(s) | Assay / calculation information | Reported unit(s) | Fasting status | Blood-sampling timing |
| --- | --- | --- | --- | --- | --- | --- |
| 1 | Gyorkos 2019 | hs-CRP; IL-6; TNF-α; HOMA-IR | hs-CRP: turbidimetric immunoassay reagent kit with SMART automated analyzer. IL-6/TNF-α: commercial ELISA kits (Thermo Fisher Scientific). HOMA-IR: calculated from fasting glucose and insulin; glucose by Cholestech LDX reflectance photometry; insulin by commercial ELISA. | hs-CRP: pg/mL (as reported); IL-6/TNF-α: pg/mL; HOMA-IR: index/unitless. | Fasting whole blood. | Weeks 0, 4, 8, and 12; blood collected at least 72 h after exercise. |
| 2 | Nicklas 2004 | CRP; IL-6; TNF-α | Serum ELISA. IL-6 and TNF-α: Quantikine high-sensitivity immunoassay kits (R&D Systems). CRP: ALPCO assay. Samples measured in duplicate. | CRP: mg/L; IL-6/TNF-α: pg/mL. | Overnight fast. | Morning 07:00-09:00; baseline, 6 months, and 18 months. |
| 3 | Reljic 2022 | hs-CRP; CRP; IL-6 | CRP/hs-CRP: measured by University Hospital Erlangen laboratory; method not further specified. IL-6: commercial sandwich ELISA (R&D Systems), duplicate serum samples, Bio-Rad iMark at 450 nm. | CRP/hs-CRP: mg/L; IL-6: pg/mL. | Overnight fast; participants avoided alcohol and vigorous physical activity for 24 h. | Baseline within 1 week before intervention and post-test within 1 week after completion; at least 3 days after last training; same time of day. |
| 4 | Villareal 2006 | CRP; IL-6; HOMA-IR | CRP: ELISA (American Laboratory Products Company). IL-6: Quantikine high-sensitivity ELISA (R&D Systems). HOMA-IR: calculated from fasting glucose and insulin; glucose by glucose oxidase method. | CRP: mg/L; IL-6: pg/mL; HOMA-IR: index/unitless. | Fasting state; OGTT after overnight fast. | Baseline and 26 weeks; OGTT venous samples at fasting, 30, 60, 90, and 120 min. |
| 5 | Weiss 2016 | CRP | Serum high-sensitivity CRP ELISA, Quantikine (R&D Systems). | CRP/hs-CRP: mg/L (reported CRP outcome measured by hs-CRP ELISA). | Overnight fast. | Baseline and after the matched weight-loss or weight-stability period; blood drawn from an arm vein after overnight fast. |
| 6 | Heiston 2021 | CRP; TNF-α | CRP and TNF-α: ELISA (Millipore). | CRP: mg/L; TNF-α: pg/mL. | 10-h overnight fast. | Pre- and post-intervention during 75-g OGTT visit; final exercise bout 24 h before post-intervention OGTT; participants avoided strenuous exercise, medications, caffeine, and alcohol for 24 h. |
| 7 | Oh 2013 | hs-CRP; TNF-α; Adiponectin | TNF-α and adiponectin: ELISA kits (R&D Systems). hs-CRP: latex-enhanced immunoturbidimetric assay on ADVIA 1650 Chemistry System (Siemens). | hs-CRP: mg/mL (as reported); TNF-α: pg/mL; adiponectin: µg/mL. | Overnight fast. | Blood from antecubital vein at baseline, Month 3, and Month 6. |
| 8 | Sartor 2010 | IL-6; TNF-α | Cytokines IL-6 and TNF-α analyzed from fasting plasma samples by ELISA (Bender MedSystems GmbH, Vienna, Austria). | IL-6/TNF-α: pg/mL. | Overnight 8-12 h fast. | Pre-test and post-test; post-test 48 h after the last exercise session; measured during/around 2-h 75-g OGTT visit. |
| 9 | Schweitzer 2023 | CRP | C-reactive protein measured as part of study protocol; assay platform/manufacturer not reported. LDL-C calculated by Friedewald equation when TG <=400 mg/dL. | CRP: mg/L. | ~12-h overnight fast at home. | Baseline and within 7 days after completing 6-week lifestyle intervention or standard care. |
| 10 | Imayama 2012 | hs-CRP; IL-6 | hs-CRP: Siemens Healthcare Diagnostics assay kit. IL-6: ultrasensitive solid-phase sandwich ELISA, high-sensitivity human IL-6 immunoassay kit (R&D Systems). | hs-CRP: mg/L; IL-6: pg/mL. | 12-h fasting and no exercise for 24 h. | Baseline and 12 months; samples processed within 1 h and serum stored at -70°C. |
| 11 | Nunes 2015 | CRP; Adiponectin | Adiponectin: ELISA kits (R&D Systems). CRP: polystyrene particle-enhanced high-sensitivity turbidimetric immunoassay (Aptec Diagnostics). | CRP: mg/dL; adiponectin: ng/mL. | Overnight fast. | Pre- and post-6-month intervention; blood by venepuncture in EDTA tubes, centrifuged 3000 rpm for 10 min at 4°C. |
| 12 | Plavsic 2020 | hs-CRP; HOMA-IR | hs-CRP: particle-enhanced turbidimetric immunoassay, sensitivity 0.1 mg/L. HOMA-IR: calculated from fasting glucose and insulin; glucose by standard enzymatic method; insulin by electrochemiluminescence immunoassay (Cobas e411, Roche). | hs-CRP: mg/L; HOMA-IR: index/unitless. | 12-h fasting; participants were required to be fasted for body-composition testing and blood sampling. | Baseline and 7-15 days after intervention; early follicular phase day 3-5; blood sampling 08:00-09:00; OGTT samples at 0, 30, 60, 90, and 120 min. |
| 13 | Bouchonville 2014 | hs-CRP; Adiponectin; HOMA-IR | hs-CRP: immunoturbidimetric assay (Hitachi 917). Adiponectin: ELISA (Linco, St Louis). HOMA-IR: calculated from fasting glucose and insulin; glucose by glucose oxidase method (YSI), insulin by radioimmunoassay. | hs-CRP: mg/L; adiponectin: ng/mL; HOMA-IR: index/unitless. | Overnight fast. | Baseline, 6 months, and 12 months; OGTT samples at fasting, 30, 60, 90, and 120 min; OGTT performed ~72 h after last exercise. |
| 14 | Lefevre 2009 | Hs-CRP | hs-CRP: automated immunoassay with chemiluminescent detection on DPC-2000 instrument with manufacturer reagents. | hs-CRP: reported/analyzed after logarithmic transformation; raw unit commonly given as mg/L in article tables. | NR. | Assessed during 5-day inpatient stays at baseline, week 12/month 3, and week 24/month 6. |
| 15 | Lakhdar 2013 | IL-6; TNF-α; Adiponectin | IL-6 and TNF-α: ELISA kit (Immunotech / Beckman Coulter). Adiponectin: included in inflammatory/adipokine profile; specific assay details not clearly reported in accessible text. | IL-6/TNF-α: pg/mL; adiponectin: NR/not clearly reported. | Overnight fast. | Blood at baseline/control value, 12 weeks, and 24 weeks; morning 07:00-08:00; all testing during follicular phase. |
| 16 | Keawtep 2024 | IL-6; Adiponectin; HOMA-IR | IL-6: human ELISA kit BMS213HS (Thermo Fisher Scientific). Adiponectin: human ELISA kit KHP0041 (Thermo Fisher Scientific). HOMA-IR: calculated from fasting glucose and insulin; glucose by colorimetric assay kit; insulin by sandwich ELISA. | IL-6: pg/mL; adiponectin: ng/mL; HOMA-IR: index/unitless. | Fasting status not explicitly stated for blood sampling; HOMA-IR formula used fasting glucose and fasting insulin. | Baseline and post-3 months. |
| 17 | Bo 2007 | CRP; HOMA-IR | Current article states laboratory methods were previously described; assay platform not detailed in the article. HOMA-IR calculated from fasting glucose and insulin. | hs-CRP: mg/L; HOMA-IR: article table labels mmol/L × µU/mL/index. | Fasting blood measurements. | Before and after 1-year intervention. |
| 18 | Christiansen 2010 | IL-6; Adiponectin; HOMA-IR | IL-6: highly sensitive ELISA (R&D Systems). Adiponectin: human-specific highly sensitive ELISA (b-Bridge International). HOMA-IR: calculated from fasting glucose and insulin; insulin by ELISA (DAKO); glucose analyzed by local clinical biochemistry laboratory. | Inflammatory marker table does not label units; HOMA-IR: index/unitless. | Overnight fast. | Baseline and 12 weeks; blood collected after overnight fast and at least 24 h after last exercise; AT/SM biopsies 24-48 h after last exercise. |
| 19 | Tang 2021 | CRP; IL-6; TNF-α; HOMA-IR | CRP outcome was measured as hs-CRP by immunoturbidimetric assay. IL-6 and TNF-α: Human Luminex Discovery Assay Kit (R&D Systems). HOMA-IR: calculated from fasting plasma glucose and insulin; FPG by hexokinase method; insulin by electrochemiluminescence immunoassay. | hs-CRP/CRP: mg/L; IL-6/TNF-α: pg/mL; HOMA-IR: index/unitless. | At least 8 h fast. | Allocation/baseline and end of 8-week intervention; 5 mL venous blood, centrifuged 3000 rpm (~1000 g) for 10 min and stored at -80°C. |
| 20 | Makni 2010 (Ben Ounis et al.) | CRP; IL-6; TNF-α | IL-6 and TNF-α: Quantikine ELISA kits (R&D Systems; catalogue HS600 and HSTA00C). CRP: high-sensitivity particle-enhanced turbidimetric assay using validated commercial hsELISA kit (Kalon Biological). | CRP: mg/L; IL-6/TNF-α: pg/mL. | At least 12-h fast; no illness or injury in previous 2 weeks. | Morning blood sampling before and after the 2-month intervention; plasma stored at -80°C. |
| 21 | Nemet 2013 | IL-6; HOMA-IR | IL-6: high-sensitivity ELISA kit (R&D Systems). HOMA-IR: calculated from fasting glucose and insulin; insulin by Siemens enzyme-labeled chemiluminescent immunometric assay; glucose by Roche enzymatic reference method with hexokinase. | IL-6: pg/mL; HOMA-IR: index/unitless. | At least 12-h fast. | Early-morning blood samples at baseline and 3 months; samples spun at 3000 rpm, 4°C, 20 min; serum stored at -80°C; pre/post specimens analyzed in same batch. |
| 22 | Peña 2023 | IL-6; TNF-α | IL-6: serum multiplex immunoassay (#K15067L-2; Meso Scale Discovery). TNF-α: serum single-plex immunoassay (#K151UCK-1; Meso Scale Discovery). | IL-6/TNF-α: pg/mL. | Overnight fast. | Baseline (T1) and 6 months (T2); participants arrived fasting to clinical research unit; serum isolated and stored at -80°C. |
| 23 | Shalitin 2009 | CRP; IL-6; HOMA-IR | CRP: particle-enhanced immunoturbidimetric assay (Hitachi 904, Roche Diagnostics). IL-6: sandwich enzyme immunoassay technique (R&D Systems). HOMA-IR: calculated from fasting glucose and insulin; glucose by enzymatic colorimetric methods, insulin by immunometric assay (Immulite 2000 Analyzer, DPC). | CRP: mg/dL; IL-6: pg/mL; HOMA-IR: index/unitless. | 10-h overnight fast. | Baseline, 12 weeks, and 52 weeks. |
| 24 | Vos 2011 | Hs-CRP; Adiponectin | hs-CRP: immunoturbidimetric assay with Cobas Integra 800 (Roche Diagnostics). Adiponectin: human adiponectin radioimmunoassay kit (Millipore). | hs-CRP: mg/L; adiponectin: ng/mL. | Overnight fast; fasting state verbally confirmed by participant and parent(s). | Intervention and control groups: baseline, 3 months, and 1-year follow-up; intervention group also assessed at 2 years for anthropometry/fitness only. Serum insulin, adiponectin, and hs-CRP stored at -80°C before analysis. |

## **Supplementary Table 5.** Distribution of key effect modifiers across intervention nodes.

## **Supplementary Table 5. Part 1.** Distribution of key effect modifiers across intervention nodes.

| **Node** | **No. studies / arms** | **Age, years, median (range)** | **Metabolic syndrome** | **Duration, weeks, median (range)** | **ER prescription type** | **Exercise frequency / intensity / supervision** |
| --- | --- | --- | --- | --- | --- | --- |
| CON | 15 studies / 15 arms | 52.9 (10.4-70.0) | 5/15 studies | 26.0 (4.0-78.2) | No ER prescription | No structured exercise; usual activity or health/lifestyle advice. |
| ER | 15 studies / 15 arms | 40.9 (8.4-69.5) | 5/15 studies | 12.0 (2.0-78.2) | strict/quantified ER: 14; borderline/non-strict ER: 1 | No structured exercise; habitual physical activity maintained where reported. |
| ER+AE | 14 studies / 14 arms | 38.0 (10.4-70.0) | 4/14 studies | 24.0 (8.0-52.0) | strict/quantified ER: 11; borderline/non-strict ER: 3 | Usually 2-5 sessions/week or 150-225 min/week; mainly moderate-to-vigorous aerobic exercise; supervision varied from supervised/partly supervised to advice-based. |
| ER+HIIT | 5 studies / 5 arms | 40.9 (16.2-49.8) | 3/5 studies | 4.0 (2.0-12.0) | strict/quantified ER: 4; borderline/non-strict ER: 1 | 2-3 sessions/week; interval protocols generally ~80-95% HRpeak, >=85-90% HRmax, or ~90% VO2peak; mostly supervised and/or HR-monitored. |
| ER+COE | 6 studies / 6 arms | 34.3 (8.4-68.5) | 1/6 studies | 13.0 (6.0-78.2) | strict/quantified ER: 4; borderline/non-strict ER: 2 | Usually 2-3 sessions/week where reported; aerobic plus resistance/strength or physical-cognitive exercise; supervision and progression varied by trial. |

**Notes.** CON, control/usual care; ER, energy restriction; AE, aerobic exercise; HIIT, high-intensity interval training; COE, combined exercise; NR, not reported/available in the supplied extraction file. Age and duration are summarized at study level because complete arm-specific age and BMI data were not available for all trials in the supplied tables. Borderline/non-strict ER indicates active dietary or lifestyle-management interventions without a clearly reported fixed kcal target or prescribed energy deficit; these cases should be excluded in the strict-ER sensitivity analysis.

## **Supplementary Table 5. Part 2.** Study-level distribution of key effect modifiers for transitivity assessment

| **No.** | **Study** | **Node(s) in available outcome data** | **Age profile** | **Intervention group baseline BMI** | **Comparator baseline BMI (CON and/or ER / diet-only)** | **Metabolic status** | **Duration** | **ER prescription type** | **Exercise frequency/intensity/supervision reported** |
| --- | --- | --- | --- | --- | --- | --- | --- | --- | --- |
| 1 | Gyorkos 2019 | CON, ER, ER+HIIT | Adults; mean age 40.9 ± 20.2 years | 34.9 ± 6.3 | ER: 35.4 ± 4.3 | Metabolic syndrome / elevated cardiometabolic risk | 4 weeks; short-term | Borderline/non-strict ER: active dietary/lifestyle intervention without clearly reported quantitative energy target | intervention consisted of 3 supervised sessions per week on a cycle ergometer. Each 26-minute session included 10 intervals of 60-second sprinting at ~90% HRmax, each followed by 60 seconds of active recovery. |
| 2 | Nicklas 2004 | CON, ER, ER+COE | Older adults; control 69 ± 6, intervention 68 ± 7 years | 33.9 ± 5.6 | CON:34.5±5.3 ER: 34.4 ± 4.9 | Overweight/obesity; older sedentary population | 18 months; longer-term | Strict/quantified ER: explicit energy deficit | .intervention involved 60-minute sessions, 3 days/week. The sessions comprised 30 minutes of walking at 50-75% heart rate reserve and 15 minutes of resistance training (2 sets of 12 reps for leg extension, curl, heel raise, and step up using cuff weights/vests). |
| 3 | Reljic 2022 | ER, ER+HIIT | Adults; control 49 ± 15.1, intervention 50.6 ± 11.3 years | 37.8 ± 6.6 | CON:36.6± 5.2 | Obesity with metabolic syndrome / elevated cardiometabolic risk | 12 weeks; short-term | Strict/quantified ER: explicit energy deficit | intervention involved two 14-minute weekly sessions on cycle ergometers. Each session comprised 5 intervals of 1 minute at 80-95% HRpeak (intensity progressively increased every 4 weeks), interspersed with 1-minute recovery periods. |
| 4 | Villareal 2006 | CON, ER+AE | Older adults; control 71 ± 4, intervention 69 ± 5 years | 39 ± 5 | CON: 39 ± 5 | Obesity in older adults | 6 months; longer-term | Strict/quantified ER: explicit energy deficit | 90-minute group sessions, 3 days per week, comprising endurance exercise (≥70% VO₂peak) and flexibility exercises |
| 5 | Weiss 2016 | ER, ER+AE | Adults; control 57 ± 5, intervention 57 ± 7 years | 28.3 ± 0.4 | ER: 27.7 ± 0.4 | Overweight/obesity; postmenopausal/middle-aged sedentary population | 12 weeks; short-term | Strict/quantified ER: explicit energy deficit | intervention aimed to increase total energy expenditure by 20%. Participants used Polar HR monitors for tracking and were encouraged toward daily moderate-to-high intensity cardiovascular exercise (e.g., walking, cycling), explicitly excluding strength training. Initial 3-6 sessions were supervised. |
| 6 | Heiston 2021 | ER, ER+HIIT | Adults; control 45.6 ± 3.5, intervention 48.5 ± 3.8 years | 37.3 ± 2.0 | ER: 37.9 ± 1.6 | Obesity; female-only population | 2 weeks; short-term | Strict/quantified ER: kcal target and/or structured hypoenergy protocol | Intervention consisted of 12 supervised, 60-minute sessions comprising alternating 3-minute intervals at 50% and 90% HRpeak, with a 350-kcal shake consumed post-exercise. |
| 7 | Oh 2013 | CON, ER+AE | Adults; control 66.9 ± 9, intervention 59.8 ± 7.9 years | 26.3 ± 3.7 | CON:26.2± 3.7 | Metabolic syndrome / elevated cardiometabolic risk | 6 months; longer-term | Strict/quantified ER: fixed kcal/intake target | intervention included supervised group sessions (3/week for months 1-3, then 2/week), each 40 minutes long and expending approximately 200 kcal. Participants were also encouraged to perform daily brisk walking with pedometers and maintain an exercise diary.. |
| 8 | Sartor 2010 | ER, ER+HIIT | Adults; control 37 ± 10, intervention 41 ± 14 years | 32 ± 4 | ER: 32 ± 3 | Obesity without explicit metabolic syndrome classification | 2 weeks; short-term | Strict/quantified ER: explicit energy deficit | intervention consisted of supervised HIIT sessions performed 3 times/week for 2 weeks, comprising up to ten 4-minute cycling bouts at 90% VO₂peak interspersed with 2–3 minutes of rest, following a 1-week familiarization period. |
| 9 | Schweitzer 2023 | CON, ER+COE | Adults; control 55 ± 7, intervention 54 ± 9 years | 41.6 ± 8.3 | CON:37.7± 5.4 | Overweight/obesity without explicit metabolic syndrome classification | 6 weeks; short-term | Borderline/non-strict ER: active dietary/lifestyle intervention without clearly reported quantitative energy target | exercise intervention included 18 supervised 1-hour sessions (3 sessions/week) involving endurance exercise (targeting 70-85% of maximal heart rate), strength training, and flexibility exercises. |
| 10 | Imayama 2012 | CON, ER, ER+AE | Adults; control 57.4 ± 4.4, intervention 58 ± 4.4 years | 31.0 ± 4.3 | CON:30.7±3.9  ER: 31.1±3.9 | Overweight/obesity; postmenopausal female population | 12 months; longer-term | Strict/quantified ER: fixed kcal/intake target | intervention prescribed 225 minutes of moderate-to-vigorous activity weekly, achieved through 3 supervised facility and 2 home sessions. Participants progressively increased to 45-minute sessions at 70-85% of maximal heart rate, using equipment like treadmills and stationary bikes. |
| 11 | Nunes 2015 | CON, ER+COE | Minors; control 15.4 ± 1.20, intervention 16.18 ± 1.51 years | 34.99 ± 3.70 | CON:34.85±4.32 | Adolescent obesity | 6 months; longer-term | Borderline/non-strict ER: active dietary/lifestyle intervention without clearly reported quantitative energy target | intervention combined twice-weekly aerobic and resistance training. Aerobic sessions on a treadmill cycled through low-intensity (60% VO₂max), moderate, and high-intensity interval protocols. Resistance training used non-linear periodization across five exercises, with intensity structured in three repetition zones (2-6 RM, 8-12 RM, 14-18 RM) and loads progressing by 10%. |
| 12 | Plavsic 2020 | ER, ER+HIIT | Minors; control 15.8 ± 1.5, intervention 16.6 ± 1.3 years | 32.6 ± 2.7 | ER: 33.2 ± 3.5 | Adolescent obesity; female-only population | 12 weeks; short-term | Strict/quantified ER: explicit deficit and/or kcal target | intervention was performed 2 days per week for 43 minutes per session. The protocol consisted of a 10-minute warm-up, followed by 4 intervals of 4 minutes at 85-90% HRmax with 3-minute active recovery periods at 70% HRmax, and concluded with a 5-minute cool-down. Adherence was monitored with Polar HR monitors, and a 90% session completion rate was required. |
| 13 | Bouchonville 2014 | CON, ER, ER+AE | Older adults; control 69 ± 4, intervention 70 ± 4 years | 37.2 ± 5.4 | CON: 37.3 ± 4.7 ER: 37.2 ± 4.5 | Obesity in older adults | 12 months; longer-term | Strict/quantified ER: explicit energy deficit | intervention consisted of 30-minute sessions, conducted three times per week in a supervised facility setting. Exercise modes included treadmill, stationary cycling, and stair climbing, with intensity progressively increased from an initial 65% to 70-85% of peak heart rate |
| 14 | Lefevre 2009 | CON, ER, ER+AE | Adults; control 38 ± 8, intervention 36 ± 6 years | 27.5 ± 1.6 | CON: 27.6 ± 2.0 ER: 27.8 ± 1.4 | Overweight/obesity without explicit metabolic syndrome classification | 24 weeks; longer-term | Strict/quantified ER: explicit energy deficit | intervention was performed 5 days per week. Participants self-selected exercise intensity (47-76% VO₂max), with individual energy expenditure targets set at 403±63 kcal/session (women, ~53±11 min) and 569±118 kcal/session (men, ~45±14 min)., aiming for a 12.5% increase in total energy expenditure. |
| 15 | Lakhdar 2013 | ER, ER+AE | Adults; control 38.9 ± 3.94, intervention 38.9 ± 4.37 years | 32.98 ± 2.17 | ER: 33.02 ± 1.89 | Obesity; female-only population | 24 weeks; longer-term | Strict/quantified ER: explicit energy deficit | intervention consisted of three weekly treadmill walking/running sessions. The protocol was progressive, starting at 55% of maximal heart rate for 30 minutes during weeks 1-4, and gradually increasing to 80% of maximal heart rate for 45 minutes by weeks 20-24. |
| 16 | Keawtep 2024 | CON, ER, ER+COE | Adults; control 53.61 ± 2.81, intervention 52.17 ± 3.35 years | 29.73 ± 4.57 | CON:29.18± 2.85 ER: 28.28 ± 2.78 | Obesity; postmenopausal female population | 3 months; short-term | Strict/quantified ER: explicit deficit and/or quantified hypoenergy protocol | intervention involved 60-minute sessions (10-min warm-up, 40-min core exercise, 10-min cool-down) conducted 3 days per week. The core exercise combined aerobic dance (e.g., high knee marching) with resistance training for major muscle groups . |
| 17 | Bo 2007 | CON, ER+AE | Adults; control 55.7 ± 5.7, intervention 55.7 ± 5.6 years | 29.7 ± 4.1 | CON: 29.8 ± 4.6 | Metabolic syndrome / elevated cardiometabolic risk | 12 months; longer-term | Borderline/non-strict ER: active dietary/lifestyle intervention without clearly reported quantitative energy target | intervention provided personalized advice, recommending at least 150 minutes per week of moderate-intensity activity, such as brisk walking, in line with established guidelines.. |
| 18 | Christiansen 2010 | ER, ER+AE | Adults; control 35.6 ± 7, intervention 37.5 ± 8 years | 34.2 ± 3 | ER: 35.3 ± 4 | Obesity without explicit metabolic syndrome classification | 12 weeks; short-term | Strict/quantified ER: kcal target and/or structured hypoenergy protocol | supervised aerobic exercise 3 times per week, with each session lasting 60-75 minutes and designed to expend 500-600 kcal. |
| 19 | Tang 2021 | ER, ER+AE | Adults; control 19.4 ± 0.6, intervention 19.4 ± 0.7 years | 24.5 ± 2.4 | ER: 24.8 (2.5) | Overweight/obesity; young-adult population | 8 weeks; short-term | Strict/quantified ER: fixed kcal/intake target | intervention prescribed individualized rope skipping exercise 3 times per week, with each 90-minute session structured as 20 minutes of skipping followed by 10 minutes of rest, repeated 3 times. The target intensity was 1,500 skips per session, maintained after a 2-week adaptation period. |
| 20 | Makni 2010 | CON, ER+AE | Minors; control 13.3 ± 0.6, intervention 13.1 ± 0.8 years | 31.1 ± 1.1 | CON: 30.7 ± 1.6 | Childhood obesity | 8 weeks; short-term | Strict/quantified ER: explicit energy deficit | intervention involved children in 90-minute sessions, 4 times per week. Activities included running, jumping, and ball play, with exercise intensity individually fixed at the heart rate corresponding to each participant's Lipox max. |
| 21 | Nemet 2013 | CON, ER+AE, ER+COE | Minors; control 10.49 ± 2.67, intervention 10.41 ± 1.96 years | 27.1± 5.31 | CON:26.6± 4.68 | Childhood obesity | 3 months; short-term | Strict/quantified ER: explicit deficit and/or kcal target | intervention for children involved twice-weekly, 1-hour group sessions primarily consisting of endurance games (50% team sports, 50% running games). Additionally, participants were instructed to perform an extra 30–45 minutes of weight-bearing sport activities at least once a week. |
| 22 | Peña 2023 | CON, ER+AE | Minors; control 13.2 ± 1.4, intervention 13.4 ± 1.4 years | 32.1 ± 3.7 | CON: 33.7 ± 6.3 | Adolescent obesity; ethnicity-specific sample | 6 months; longer-term | Borderline/non-strict ER: active dietary/lifestyle intervention without clearly reported quantitative energy target | intervention included 2 weekly 60-min YMCA sessions (circuit/cardiovascular training, sports) with ≥150 bpm target heart rate, plus 1 monitored weekly session to reach 180 mins/week of moderate-to-vigorous aerobic activity.t. |
| 23 | Shalitin 2009 | ER, ER+COE | Minors; control 8.51 ± 1.52, intervention 8.2 ± 1.56 years | 25.9 ± 0.51 | ER: 26.5 ± 0.51 | Childhood obesity | 12 weeks; short-term | Strict/quantified ER: fixed kcal/intake target | intervention involved 90-minute supervised sessions, conducted 3 days per week. Each session was equally divided, with 45 minutes dedicated to aerobic exercise (team sports and running games) and 45 minutes dedicated to resistance training (including exercises such as sit-ups, hand-lifting of small weights, and ball exercises). |
| 24 | Vos 2011 | CON, ER+AE | Minors; control 13.1 ± 1.9, intervention 13.3 ± 2.0 years | 32.4 ± 4.7 | CON: 32.5 ± 3.9 | Childhood/adolescent obesity | 3 months; short-term | Borderline/non-strict ER: active dietary/lifestyle intervention without clearly reported quantitative energy target | increasing aerobic activities like walking/cycling and reducing sedentary behavior to optimize daily aerobic exercise engagement. |

**Supplementary Figure 1.** Risk of Bias Assessment

**Supplementary Figure 1. Part 1.** Risk of bias in included studies assessed with the RoB 2 tool

**Supplementary Figure 1. Part 2.** Network of eligible comparisons for different inflammation-related outcomes showing the Confidence in Network Meta-Analysis (CINeMA) to evaluate the certainty of the evidence. Green is low risk bias, yellow is some concerns and red is high risk of bias based on ROB2 tool

(A)hs-CRP	(B)CRP	(C)TNF-α

(D)IL-6	(E)Adiponectin	(F)HOMA-IR

**Supplementary Figure 1. Part 3.** Confidence In Network Meta Analysis - CINeMA 2.0.0

**Supplementary Figure 2.** Contributions of direct and indirect comparisons to NMA and the number of studies of each direct comparison of hs-CRP, CRP,TNF-α,IL-6,Adiponectin and HOMA-IR.(Note: A-CON, B-ER, C-ER+AE, D-ER+HIIT, ER+COE;Figures were produced with Stata version 15.1.)

**Supplementary Figure 2. Part 1.** hs-CRP

**Supplementary Figure 1. Part 1.** CRP

**Supplementary Figure 2. Part 3. TNF-α**

**Supplementary Figure 2. Part 4. IL-6**

**Supplementary Figure 2. Part 5. a**diponectin

**Supplementary Figure 2. Part 6.** HOMA-IR

**Supplementary Figure 3.** Forest plots of eligible comparisons of hs-CRP, CRP,TNF-α,IL-6,Adiponectin and HOMA-IR.(Figures were produced with Stata version 15.1.)

**Supplementary Figure 3. Part 1.** hs-CRP

**Supplementary Figure 3. Part 2.** CRP

**Supplementary Figure 3. Part 3. TNF-α**

**Supplementary Figure 3. Part 4.** IL-6

**Supplementary Figure 3. Part 5.** Adiponectin

**Supplementary Figure 3. Part 6.** HOMA-IR

**Supplementary Table 6.** Heterogeneity diagnostics by outcome (random-effects network meta-analysis)

**Supplementary Table 6.part 1.** The table summarizes heterogeneity diagnostics for each outcome-specific network. Between-study heterogeneity was quantified using the common τ² from the random-effects NMA model (REML). I² and Cochran’s Q are reported as complementary measures; interpretation should consider prediction intervals for key contrasts.

| **Outcome** | **No. of trials (k)** | **Total participants (N)** | **τ² (common)** | **τ = √τ²** | **I² (95% CI)** | **Cochran’s Q (df), p-value** | **Interpretation / Notes** |
| --- | --- | --- | --- | --- | --- | --- | --- |
| hs-CRP | 10 | 1,027 | 0.0000 | 0.000 | 12.5% (0.0%–53.0%) | 11.43 (df=10), p=0.325 | Low heterogeneity; no statistical evidence of heterogeneity by Q test. |
| CRP | 9 | 468 | 0.0000 | 0.000 | 0.0% (0.0%–70.8%) | 1.62 (df=6), p=0.951 | Low heterogeneity; no statistical evidence of heterogeneity by Q test. |
| TNF-α | 9 | 412 | 0.0000 | 0.000 | 0.0% (0.0%–70.8%) | 5.84 (df=6), p=0.441 | Low heterogeneity; no statistical evidence of heterogeneity by Q test. |
| IL-6 | 14 | 938 | 0.0429 | 0.207 | 27.6% (0.0%–61.7%) | 17.95 (df=13), p=0.160 | Moderate heterogeneity; no statistical evidence of heterogeneity by Q test. |
| Adiponectin | 7 | 386 | 0.0000 | 0.000 | 0.0% (0.0%–74.6%) | 3.14 (df=5), p=0.679 | Low heterogeneity; no statistical evidence of heterogeneity by Q test. |
| HOMA-IR | 11 | 748 | 0.0455 | 0.213 | 13.4% (0.0%–54.6%) | 10.39 (df=9), p=0.320 | Low heterogeneity; no statistical evidence of heterogeneity by Q test. |

**Notes:**

1) τ²/τ are reported on the SMD scale (Hedges’ g) under a common heterogeneity assumption across comparisons within each outcome-specific network (random-effects NMA; REML estimation).

2) I² is interpreted using conventional thresholds (not important: 0–30%; moderate: 30–50%; substantial: 50–75%; considerable: 75–100%) and should be interpreted alongside clinical and methodological considerations.

3) Cochran’s Q test has limited power when few studies contribute; p-values should be interpreted cautiously.

4) For key contrasts, report 95% prediction intervals to reflect the range of effects expected in a new trial (recommended to present in Appendix S5 or S6).

**Supplementary Table 6.part 2.** Inconsistency of primary and secondary outcomes tested by loop-specific heterogeneity estimates, inconsistency model and node splitting analysis.(Note: A-CON, B-ER, C-ER+AE, D-ER+HIIT, ER+COE)

| loop-specific heterogeneity estimates | | | | | | |
| --- | --- | --- | --- | --- | --- | --- |
| hs-CRP | | | | | | |
| LOOP | IF | seIF | Z_value | P_value | CI_95 | Loop_Hete  Rog_tau2 |
| CON-ER-ER+AE | 0.09822951 | 0.2620771 | 0.3748115 | 0.708 | (0.00,0.61) | 0.030 |
| CRP | | | | | | |
| CON-ER+AE-ER-ER+COE | 0.42710691 | 0.4796242 | 0.89050334 | 0.373 | (0.00,1.37) | 0.000 |
| CON-ER-ER+AE | 0.41608392 | 0.4690070 | 0.88715922 | 0.375 | (0.00,1.34) | 0.000 |
| CON-ER-ER+COE | 0.01102299 | 0.2837580 | 0.03884644 | 0.969 | (0.00,0.57) | 0.000 |
| TNF-α | | | | | | |
| CON-ER+AE-ER-ER+COE | 0.1421454 | 0.4315239 | 0.3294034 | 0.742 | (0.00,0.99) | 0.000 |
| CON-ER+AE-ER-ER+HIIT | 1.0659745 | 0.7217537 | 1.4769228 | 0.140 | (0.00,2.48) | 0.000 |
| CON-ER+HIIT-ER-ER+COE | 1.2081199 | 0.6928197 | 1.7437725 | 0.081 | (0.00,2.57) | 0.000 |
| CON-ER-ER+AE | 0.1421454 | 0.3857207 | 0.3685190 | 0.712 | (0.00,0.90) | 0.000 |
| CON-ER-ER+COE | 0.0000000 | 0.3284070 | 0.0000000 | 1.000 | (0.00,0.64) | 0.000 |
| CON-ER-ER+HIIT | 1.2081199 | 0.6652569 | 1.8160201 | 0.069 | (0.00,2.51) | 0.000 |
| IL-6 | | | | | | |
| CON-ER+AE-ER-ER+COE | 0.06406709 | 0.3072789 | 0.2084982 | 0.835 | (0.00,0.67) | 0.025 |
| CON-ER-ER+AE | 0.15608621 | 0.2775679 | 0.5623352 | 0.574 | (0.00,0.70) | 0.047 |
| CON-ER-ER+COE | 0.22015330 | 0.2672737 | 0.8236997 | 0.410 | (0.00,0.74) | 0.013 |
| Adiponectin | | | | | | |
| CON-ER+AE-ER-ER+COE | 0.5405432 | 0.4668236 | 1.1579174 | 0.247 | (0.00,1.46) | 0.000 |
| CON-ER-ER+AE | 0.2641469 | 0.3906358 | 0.6761974 | 0.499 | (0.00,1.03) | 0.031 |
| CON-ER-ER+COE | 0.2763964 | 0.4923036 | 0.5614348 | 0.575 | (0.00,1.24) | 0.047 |
| HOMA-IR | | | | | | |
| CON-ER+AE-ER-ER+COE | 0.2199894 | 0.4442402 | 0.4952037 | 0.620 | (0.00,1.09) | 0.042 |
| CON-ER-ER+AE | 0.1591548 | 0.3849049 | 0.4134912 | 0.679 | (0.00,0.91) | 0.057 |
| CON-ER-ER+COE | 0.3791442 | 0.3636533 | 1.0425979 | 0.297 | (0.00,1.09) | 0.000 |

| Inconsistency model | | | | | | |
| --- | --- | --- | --- | --- | --- | --- |
|  | hs-CRP | CRP | TNF-α | IL-6 | Adiponectin | HOMA-IR |
| chi2 | 2.01 | 0.90 | 3.31 | 8.78 | 2.49 | 6.34 |
| Prob>chi2 | 0.37 | 0.83 | 0.19 | 0.07 | 0.65 | 0.27 |

| Node splitting analysis | | | | | | | | |
| --- | --- | --- | --- | --- | --- | --- | --- | --- |
| hs-CRP | | | | | | | | |
| Side |  | Direct  Coef. | Std.  Err. | Indirect  Coef. | Std.  Err. | Difference  Coef. | Std.  Err. | P>Z |
| A | B | -0.4085162 | 0.1547673 | -0.0293719 | 0.3058931 | -0.3791443 | 0.3520142 | 0.281 |
| A | C* | -0.6125323 | 0.0986934 | -1.687211 | 0.8969285 | 1.074679 | 0.9064935 | 0.236 |
| B | C* | -0.3224146 | 0.1451883 | -0.0350798 | 0.3747165 | -0.2873348 | 0.392003 | 0.464 |
| B | D* | -0.1067778 | 0.1983051 | 0.6537698 | 359.15 | -0.7605476 | 359.15 | 0.998 |
| CRP | | | | | | | | |
| Side |  | Direct  Coef. | Std.  Err. | Indirect  Coef. | Std.  Err. | Difference  Coef. | Std.  Err. | P>Z |
| A | B | -0.058203 | 0.1884794 | -0.201081 | 0.2753535 | 0.142878 | 0.333346 | 0.668 |
| A | C | -0.7810766 | 0.2857401 | -0.3568087 | 0.37502 | -0.4242679 | 0.4714737 | 0.368 |
| A | E | 0.0005085 | 0.1458881 | -0.3633082 | 0.4776077 | 0.3638166 | 0.5002434 | 0.467 |
| B | C | -0.3059188 | 0.3360836 | -0.7301864 | 0.3306589 | 0.4242676 | 0.4714737 | 0.368 |
| B | D* | -0.3141615 | 0.2295785 | 0.2152598 | 438.4813 | -0.5294213 | 438.4813 | 0.999 |
| B | E | 0.0459032 | 0.1576517 | 0.278321 | 0.4368398 | -0.2324178 | 0.464411 | 0.617 |
| TNF-α | | | | | | | | |
| Side |  | Direct  Coef. | Std.  Err. | Indirect  Coef. | Std.  Err. | Difference  Coef. | Std.  Err. | P>Z |
| A | B* | 0.018644 | 0.1885091 | -0.342517 | 0.3105134 | 0.3611609 | 0.3632551 | 0.32 |
| A | C | -0.5758423 | 0.1745873 | -0.5173601 | 0.3518352 | -0.0584822 | 0.3927706 | 0.882 |
| A | D | -0.7534186 | 0.6141363 | 0.4223947 | 0.3485689 | -1.175813 | 0.7061613 | 0.096 |
| A | E* | -0.0593217 | 0.1885352 | -0.7816436 | 0.7047762 | 0.7223219 | 0.7265101 | 0.32 |
| B | C | -0.4512692 | 0.3012384 | -0.509751 | 0.2520402 | 0.0584818 | 0.3927707 | 0.882 |
| B | D | 0.4362164 | 0.3066271 | -0.7395959 | 0.6361155 | 1.175812 | 0.7061609 | 0.096 |
| B | E* | -0.0779657 | 0.1943057 | 0.6443562 | 0.7000586 | -0.7223219 | 0.7265102 | 0.32 |
| IL-6 | | | | | | | | |
| Side |  | Direct  Coef. | Std.  Err. | Indirect  Coef. | Std.  Err. | Difference  Coef. | Std.  Err. | P>Z |
| A | B | -0.5561562 | 0.1359327 | -0.0646552 | 0.2676467 | -0.491501 | 0.3053338 | 0.107 |
| A | C | -0.7790599 | 0.1571147 | -0.7773912 | 0.3255085 | -0.0016687 | 0.3651905 | 0.996 |
| A | E | -0.2694909 | 0.2076769 | -0.5386692 | 0.3530886 | 0.2691783 | 0.4136551 | 0.515 |
| B | C | -0.3946577 | 0.1728607 | -0.2166184 | 0.2864316 | -0.1780393 | 0.3350227 | 0.595 |
| B | D* | -0.267035 | 0.2536637 | 0.8796098 | 363.5322 | -1.146645 | 363.5323 | 0.997 |
| B | E* | 0.0583031 | 0.1769559 | 0.4127387 | 0.5354373 | -0.3544356 | 0.5606156 | 0.527 |
| Adiponectin | | | | | | | | |
| Side |  | Direct  Coef. | Std.  Err. | Indirect  Coef. | Std.  Err. | Difference  Coef. | Std.  Err. | P>Z |
| A | B | 0.2847651 | 0.2046811 | 0.0879511 | 0.3306788 | 0.196814 | 0.3888419 | 0.613 |
| A | C | 0.148328 | 0.1519846 | 0.7005339 | 0.4314165 | -0.5522059 | 0.4574338 | 0.227 |
| A | E* | 0.9345444 | 0.2535811 | -0.1950397 | 0.6817464 | 1.129584 | 0.7292694 | 0.121 |
| B | C | 0.1092824 | 0.2065954 | -0.3639056 | 0.3347226 | 0.4731879 | 0.3927646 | 0.228 |
| B | D* | -0.3148158 | 0.2792342 | -0.4517573 | 615.8691 | 0.1369415 | 615.8691 | 1 |
| B | E | 0.3549513 | 0.3034993 | 1.027764 | 0.4506692 | -0.6728132 | 0.5431309 | 0.215 |
| HOMA-IR | | | | | | | | |
| Side |  | Direct  Coef. | Std.  Err. | Indirect  Coef. | Std.  Err. | Difference  Coef. | Std.  Err. | P>Z |
| A | B | -0.4423027 | 0.2100916 | -1.021977 | 0.2681318 | 0.5796745 | 0.3424574 | 0.091 |
| A | C | -0.8769303 | 0.2051329 | -0.9101215 | 0.3918411 | 0.0331913 | 0.4424858 | 0.94 |
| A | E | -1.026014 | 0.2735522 | -0.7193388 | 0.4194493 | -0.3066748 | 0.5012267 | 0.541 |
| B | C | -0.175638 | 0.1947764 | -0.4185574 | 0.3796045 | 0.2429193 | 0.4293336 | 0.572 |
| B | D* | -0.3502322 | 0.3104217 | 1.360283 | 450.5847 | -1.710515 | 450.5848 | 0.997 |
| B | E | -0.2112821 | 0.2526016 | -0.4806992 | 0.457209 | 0.2694171 | 0.5215834 | 0.605 |

* All the evidence about these contrasts comes from the trials which directly compare them.

Note: A = CON, B = ER, C = ER+AE, D = ER+HIIT, E = ER+COE.

**Supplementary Table 6. Part 3.** Contextual comparisons: ER+exercise versus CON

| **Outcome** | **Comparison** | **No. studies** | **SMD (95% CI)** | **Certainty** | **Main reasons for downgrading** | **Interpretation** |
| --- | --- | --- | --- | --- | --- | --- |
| **hs-CRP** | ER+AE vs CON | 6 | -0.63 (-0.80, -0.46) | High | No major downgrading concerns | Favours ER+AE |
| hs-CRP | ER+HIIT vs CON | -- | -0.44 (-0.90, 0.02) | Moderate | Imprecision | No clear difference |
| **CRP** | ER+AE vs CON | 2 | -0.64 (-1.09, -0.20) | Moderate | Within-study bias | Favours ER+AE |
| CRP | ER+HIIT vs CON | -- | -0.43 (-0.97, 0.12) | Low | Imprecision; Heterogeneity | No clear difference |
| CRP | ER+COE vs CON | 3 | -0.03 (-0.31, 0.24) | Low | Imprecision | No clear difference |
| **TNF-α** | ER+AE vs CON | 3 | -0.58 (-0.89, -0.27) | Moderate | Within-study bias | Favours ER+AE |
| TNF-α | ER+HIIT vs CON | 1 | 0.13 (-0.46, 0.72) | Low | Imprecision | No clear difference |
| TNF-α | ER+COE vs CON | 1 | -0.11 (-0.47, 0.25) | Low | Imprecision | No clear difference |
| **IL-6** | ER+AE vs CON | 5 | -0.80 (-1.05, -0.54) | High | No major downgrading concerns | Favours ER+AE |
| IL-6 | ER+HIIT vs CON | -- | -0.72 (-1.28, -0.17) | High | No major downgrading concerns | Favours ER+HIIT |
| IL-6 | ER+COE vs CON | 2 | -0.34 (-0.69, 0.00) | Moderate | Imprecision | No clear difference |
| **Adiponectin** | ER+AE vs CON | 3 | 0.21 (-0.07, 0.49) | Moderate | Imprecision | No clear difference |
| Adiponectin | ER+HIIT vs CON | -- | -0.09 (-0.73, 0.56) | Low | Imprecision | No clear difference |
| Adiponectin | ER+COE vs CON | 2 | 0.81 (0.35, 1.27) | High | No major downgrading concerns | Favours ER+COE |
| **HOMA-IR** | ER+AE vs CON | 3 | -0.97 (-1.22, -0.73) | High | No major downgrading concerns | Favours ER+AE |
| HOMA-IR | ER+HIIT vs CON | -- | -1.07 (-1.71, -0.44) | High | No major downgrading concerns | Favours ER+HIIT |
| HOMA-IR | ER+COE vs CON | 2 | -0.96 (-1.35, -0.58) | High | No major downgrading concerns | Favours ER+COE |

**Notes.** Values are presented as SMD (95% CI) for the first treatment versus the comparator. For hs-CRP, CRP, TNF-α, IL-6, and HOMA-IR, negative SMDs favour the first treatment. For adiponectin, positive SMDs favour the first treatment. Certainty of evidence was assessed using CINeMA 2.0.0. Main downgrade reasons reflect CINeMA domains. ER, energy restriction; CON, control/usual care; AE, aerobic exercise; HIIT, high-intensity interval training; COE, combined exercise; SMD, standardized mean difference; CI, confidence interval.

**Supplementary Figure 4.** The funnel plot graphics of hs-CRP, CRP,TNF-α,IL-6,Adiponectin and HOMA-IR in NMA((Figures were produced with Stata version 15.1.)

**Supplementary Figure 4. Part 1.** hs-CRP

**Supplementary Figure 4. Part 2.** CRP

**Supplementary Figure 4. Part 3.** TNF-α

**Supplementary Figure 4. Part 4.** IL-6

**Supplementary Figure 4. Part 5.** Adiponectin

**Supplementary Figure 4. Part 6.** HOMA-IR

**Supplementary Table 7.**Sensitivity analysis results of the network meta-analysis

**Supplementary Table 7.part 1.** Definitions and exclusion rules for sensitivity analyses.

| **Sensitivity analysis** | **Dataset rule** | **Excluded studies** | **Purpose / interpretation** |
| --- | --- | --- | --- |
| SA1-strict-ER | Strict ER sensitivity analysis; borderline or non-strict ER studies were excluded. | Bo 2007; Gyorkos 2019; Nunes 2015; Peña 2023; Schweitzer 2023; Vos 2011. | Tests whether the findings remain stable when ER is restricted to studies with clearly quantified energy restriction. |
| SA2-excluding-minors | Adult/older-adult sensitivity analysis; studies involving minors were excluded. | Makni 2010; Nemet 2013; Nunes 2015; Peña 2023; Plavsic 2020; Shalitin 2009; Vos 2011. | Tests whether the findings are robust after removing pediatric/adolescent populations. |
| SA3-excluding-high-RoB | Risk-of-bias sensitivity analysis; trials judged as high overall risk of bias were excluded. | Lakhdar 2013; Plavsic 2020; Schweitzer 2023; Tang 2021. | Tests whether conclusions are influenced by studies with high overall methodological risk. |
| SA4-longer-term-only | Intervention-duration sensitivity analysis; only longer-term interventions were retained and short-term trials were excluded. | Gyorkos 2019; Reljic 2022; Weiss 2016; Heiston 2021; Sartor 2010; Schweitzer 2023; Plavsic 2020; Keawtep 2024; Christiansen 2010; Tang 2021; Makni 2010; Nemet 2013; Shalitin 2009; Vos 2011. | Tests whether effects are stable among studies with a longer intervention duration rather than early short-term responses. |
| SA5-BMI-ge30-only | Baseline-BMI sensitivity analysis; only studies with reported mean baseline BMI >=30 kg/m^2 were retained. | Bo 2007; Keawtep 2024; Lefevre 2009; Nemet 2013; Oh 2013; Shalitin 2009; Tang 2021; Weiss 2016. | Tests whether the findings remain stable after excluding studies with comparatively lower baseline BMI, thereby assessing the influence of baseline adiposity on the network estimates. |
| SA6-excluding-MetS-preDM | Metabolic-status sensitivity analysis; studies in metabolic syndrome, or elevated cardiometabolic-risk populations were excluded. | Bo 2007; Gyorkos 2019; Oh 2013; Peña 2023; Reljic 2022. | Tests whether the estimates are driven by populations with metabolic syndrome, or elevated cardiometabolic risk. |

**Supplementary Table 7.part 2. Data availability and model-fitting status for each sensitivity analysis.**

| **Sensitivity** | **Outcome** | **Raw-rows** | **Valid-arm-rows** | **Analyzable-studies** | **Participants-arm-sum** | **Nodes** | **Pairwise-contrasts** |
| --- | --- | --- | --- | --- | --- | --- | --- |
| SA1-strict-ER | hs-CRP | 17 | 17 | 7 | 611 | ER+HIIT, ER+AE, ER, CON | 13 |
| SA1-strict-ER | CRP | 15 | 15 | 7 | 389 | ER+COE, ER+HIIT, ER+AE, ER, CON | 9 |
| SA1-strict-ER | TNF-alpha | 15 | 15 | 7 | 336 | ER+COE, ER+HIIT, ER+AE, ER, CON | 9 |
| SA1-strict-ER | IL-6 | 27 | 27 | 12 | 862 | ER+COE, ER+HIIT, ER+AE, ER, CON | 18 |
| SA1-strict-ER | Adiponectin | 12 | 12 | 5 | 292 | ER+COE, ER+HIIT, ER+AE, ER, CON | 9 |
| SA1-strict-ER | HOMA-IR | 20 | 20 | 9 | 401 | ER+COE, ER+HIIT, ER+AE, ER, CON | 13 |
| SA2-excluding-minors | hs-CRP | 19 | 19 | 8 | 914 | ER+HIIT, ER+AE, ER, CON | 14 |
| SA2-excluding-minors | CRP | 13 | 13 | 6 | 360 | ER+COE, ER+HIIT, ER+AE, ER, CON | 8 |
| SA2-excluding-minors | TNF-alpha | 15 | 15 | 7 | 320 | ER+COE, ER+HIIT, ER+AE, ER, CON | 9 |
| SA2-excluding-minors | IL-6 | 23 | 23 | 10 | 750 | ER+COE, ER+HIIT, ER+AE, ER, CON | 16 |
| SA2-excluding-minors | Adiponectin | 12 | 12 | 5 | 292 | ER+COE, ER+HIIT, ER+AE, ER, CON | 9 |
| SA2-excluding-minors | HOMA-IR | 18 | 18 | 8 | 608 | ER+COE, ER+HIIT, ER+AE, ER, CON | 12 |
| SA3-excluding-high-RoB | hs-CRP | 19 | 19 | 8 | 957 | ER+HIIT, ER+AE, ER, CON | 14 |
| SA3-excluding-high-RoB | CRP | 17 | 17 | 8 | 414 | ER+COE, ER+HIIT, ER+AE, ER, CON | 10 |
| SA3-excluding-high-RoB | TNF-alpha | 15 | 15 | 7 | 366 | ER+COE, ER+HIIT, ER+AE, ER, CON | 9 |
| SA3-excluding-high-RoB | IL-6 | 27 | 27 | 12 | 892 | ER+COE, ER+HIIT, ER+AE, ER, CON | 18 |
| SA3-excluding-high-RoB | Adiponectin | 16 | 16 | 7 | 386 | ER+COE, ER+HIIT, ER+AE, ER, CON | 11 |
| SA3-excluding-high-RoB | HOMA-IR | 18 | 18 | 8 | 658 | ER+COE, ER+HIIT, ER+AE, ER, CON | 12 |
| SA4-longer-term-only | hs-CRP | 13 | 13 | 5 | 824 | ER+AE, ER, CON | 11 |
| SA4-longer-term-only | CRP | 7 | 7 | 3 | 218 | ER+COE, ER+AE, ER, CON | 5 |
| SA4-longer-term-only | TNF-alpha | 9 | 9 | 4 | 302 | ER+COE, ER+AE, ER, CON | 6 |
| SA4-longer-term-only | IL-6 | 12 | 12 | 5 | 598 | ER+COE, ER+AE, ER, CON | 9 |
| SA4-longer-term-only | Adiponectin | 7 | 7 | 3 | 158 | ER+COE, ER+AE, ER, CON | 5 |
| SA4-longer-term-only | HOMA-IR | 9 | 9 | 4 | 463 | ER+AE, ER, CON | 6 |
| SA5-BMI-ge30-only | hs-CRP | 14 | 14 | 6 | 579 | ER+HIIT, ER+AE, ER, CON | 10 |
| SA5-BMI-ge30-only | CRP | 15 | 15 | 7 | 377 | ER+COE, ER+HIIT, ER+AE, ER, CON | 9 |
| SA5-BMI-ge30-only | TNF-alpha | 15 | 15 | 7 | 334 | ER+COE, ER+HIIT, ER+AE, ER, CON | 9 |
| SA5-BMI-ge30-only | IL-6 | 22 | 22 | 10 | 749 | ER+COE, ER+HIIT, ER+AE, ER, CON | 14 |
| SA5-BMI-ge30-only | Adiponectin | 11 | 11 | 5 | 267 | ER+COE, ER+HIIT, ER+AE, ER, CON | 7 |
| SA5-BMI-ge30-only | HOMA-IR | 13 | 13 | 6 | 224 | ER+HIIT, ER+AE, ER, CON | 8 |

**Supplementary Table 7.part 3. Main sensitivity-analysis comparisons.**

| **Sensitivity** | **Outcome** | **Comparison** | **SMD-95CI** | **P-value** | **Significant** |
| --- | --- | --- | --- | --- | --- |
| SA1-strict-ER | hs-CRP | ER vs CON | -0.40 (-0.72, -0.07) | 0.017 | Yes |
| SA1-strict-ER | hs-CRP | ER+AE vs CON | -0.71 (-1.01, -0.41) | <0.001 | Yes |
| SA1-strict-ER | hs-CRP | ER+HIIT vs CON | -0.49 (-1.06, 0.08) | 0.094 | No |
| SA1-strict-ER | CRP | ER vs CON | -0.11 (-0.43, 0.21) | 0.505 | No |
| SA1-strict-ER | CRP | ER+AE vs CON | -0.63 (-1.07, -0.19) | 0.005 | Yes |
| SA1-strict-ER | CRP | ER+HIIT vs CON | -0.42 (-0.97, 0.12) | 0.128 | No |
| SA1-strict-ER | CRP | ER+COE vs CON | -0.04 (-0.38, 0.30) | 0.817 | No |
| SA1-strict-ER | TNF-alpha | ER vs CON | -0.02 (-0.35, 0.31) | 0.909 | No |
| SA1-strict-ER | TNF-alpha | ER+AE vs CON | -0.56 (-0.94, -0.19) | 0.003 | Yes |
| SA1-strict-ER | TNF-alpha | ER+HIIT vs CON | 0.42 (-0.25, 1.08) | 0.219 | No |
| SA1-strict-ER | TNF-alpha | ER+COE vs CON | -0.08 (-0.44, 0.28) | 0.674 | No |
| SA1-strict-ER | IL-6 | ER vs CON | -0.47 (-0.72, -0.21) | <0.001 | Yes |
| SA1-strict-ER | IL-6 | ER+AE vs CON | -0.85 (-1.12, -0.58) | <0.001 | Yes |
| SA1-strict-ER | IL-6 | ER+HIIT vs CON | -0.64 (-1.21, -0.06) | 0.030 | Yes |
| SA1-strict-ER | IL-6 | ER+COE vs CON | -0.36 (-0.69, -0.02) | 0.035 | Yes |
| SA1-strict-ER | Adiponectin | ER vs CON | 0.28 (-0.08, 0.63) | 0.129 | No |
| SA1-strict-ER | Adiponectin | ER+AE vs CON | 0.31 (-0.04, 0.66) | 0.079 | No |
| SA1-strict-ER | Adiponectin | ER+HIIT vs CON | -0.04 (-0.69, 0.61) | 0.907 | No |
| SA1-strict-ER | Adiponectin | ER+COE vs CON | 0.80 (0.25, 1.34) | 0.004 | Yes |
| SA1-strict-ER | HOMA-IR | ER vs CON | -0.50 (-0.83, -0.18) | 0.003 | Yes |
| SA1-strict-ER | HOMA-IR | ER+AE vs CON | -0.61 (-0.96, -0.25) | <0.001 | Yes |
| SA1-strict-ER | HOMA-IR | ER+HIIT vs CON | -0.86 (-1.53, -0.19) | 0.012 | Yes |
| SA1-strict-ER | HOMA-IR | ER+COE vs CON | -0.84 (-1.20, -0.47) | <0.001 | Yes |
| SA2-excluding-minors | hs-CRP | ER vs CON | -0.37 (-0.64, -0.10) | 0.007 | Yes |
| SA2-excluding-minors | hs-CRP | ER+AE vs CON | -0.68 (-0.89, -0.47) | <0.001 | Yes |
| SA2-excluding-minors | hs-CRP | ER+HIIT vs CON | -0.65 (-1.24, -0.07) | 0.029 | Yes |
| SA2-excluding-minors | CRP | ER vs CON | -0.09 (-0.42, 0.25) | 0.600 | No |
| SA2-excluding-minors | CRP | ER+AE vs CON | -0.54 (-1.08, -0.01) | 0.048 | Yes |
| SA2-excluding-minors | CRP | ER+HIIT vs CON | -0.40 (-0.96, 0.15) | 0.152 | No |
| SA2-excluding-minors | CRP | ER+COE vs CON | 0.03 (-0.27, 0.32) | 0.867 | No |
| SA2-excluding-minors | TNF-alpha | ER vs CON | -0.06 (-0.38, 0.27) | 0.732 | No |
| SA2-excluding-minors | TNF-alpha | ER+AE vs CON | -0.46 (-0.88, -0.03) | 0.036 | Yes |
| SA2-excluding-minors | TNF-alpha | ER+HIIT vs CON | 0.14 (-0.43, 0.71) | 0.636 | No |
| SA2-excluding-minors | TNF-alpha | ER+COE vs CON | -0.09 (-0.45, 0.26) | 0.605 | No |
| SA2-excluding-minors | IL-6 | ER vs CON | -0.55 (-0.80, -0.31) | <0.001 | Yes |
| SA2-excluding-minors | IL-6 | ER+AE vs CON | -0.99 (-1.28, -0.70) | <0.001 | Yes |
| SA2-excluding-minors | IL-6 | ER+HIIT vs CON | -0.83 (-1.34, -0.31) | 0.002 | Yes |
| SA2-excluding-minors | IL-6 | ER+COE vs CON | -0.36 (-0.69, -0.02) | 0.036 | Yes |
| SA2-excluding-minors | Adiponectin | ER vs CON | 0.28 (-0.08, 0.63) | 0.129 | No |
| SA2-excluding-minors | Adiponectin | ER+AE vs CON | 0.31 (-0.04, 0.66) | 0.079 | No |
| SA2-excluding-minors | Adiponectin | ER+HIIT vs CON | -0.04 (-0.69, 0.61) | 0.907 | No |
| SA2-excluding-minors | Adiponectin | ER+COE vs CON | 0.80 (0.25, 1.34) | 0.004 | Yes |
| SA2-excluding-minors | HOMA-IR | ER vs CON | -0.60 (-0.98, -0.21) | 0.003 | Yes |
| SA2-excluding-minors | HOMA-IR | ER+AE vs CON | -0.85 (-1.18, -0.53) | <0.001 | Yes |
| SA2-excluding-minors | HOMA-IR | ER+HIIT vs CON | -0.93 (-2.14, 0.27) | 0.130 | No |
| SA2-excluding-minors | HOMA-IR | ER+COE vs CON | -0.95 (-1.60, -0.30) | 0.004 | Yes |
| SA3-excluding-high-RoB | hs-CRP | ER vs CON | -0.39 (-0.69, -0.09) | 0.012 | Yes |
| SA3-excluding-high-RoB | hs-CRP | ER+AE vs CON | -0.60 (-0.82, -0.38) | <0.001 | Yes |
| SA3-excluding-high-RoB | hs-CRP | ER+HIIT vs CON | -0.67 (-1.30, -0.04) | 0.036 | Yes |
| SA3-excluding-high-RoB | CRP | ER vs CON | -0.13 (-0.45, 0.18) | 0.409 | No |
| SA3-excluding-high-RoB | CRP | ER+AE vs CON | -0.64 (-1.07, -0.20) | 0.004 | Yes |
| SA3-excluding-high-RoB | CRP | ER+HIIT vs CON | -0.45 (-0.99, 0.09) | 0.105 | No |
| SA3-excluding-high-RoB | CRP | ER+COE vs CON | -0.08 (-0.40, 0.23) | 0.613 | No |
| SA3-excluding-high-RoB | TNF-alpha | ER vs CON | -0.10 (-0.51, 0.30) | 0.619 | No |
| SA3-excluding-high-RoB | TNF-alpha | ER+AE vs CON | -0.59 (-0.95, -0.22) | 0.002 | Yes |
| SA3-excluding-high-RoB | TNF-alpha | ER+HIIT vs CON | 0.09 (-0.52, 0.71) | 0.765 | No |
| SA3-excluding-high-RoB | TNF-alpha | ER+COE vs CON | -0.12 (-0.54, 0.31) | 0.587 | No |
| SA3-excluding-high-RoB | IL-6 | ER vs CON | -0.45 (-0.71, -0.20) | <0.001 | Yes |
| SA3-excluding-high-RoB | IL-6 | ER+AE vs CON | -0.78 (-1.03, -0.52) | <0.001 | Yes |
| SA3-excluding-high-RoB | IL-6 | ER+HIIT vs CON | -0.73 (-1.26, -0.19) | 0.008 | Yes |
| SA3-excluding-high-RoB | IL-6 | ER+COE vs CON | -0.35 (-0.68, -0.02) | 0.040 | Yes |
| SA3-excluding-high-RoB | Adiponectin | ER vs CON | 0.23 (-0.11, 0.57) | 0.180 | No |
| SA3-excluding-high-RoB | Adiponectin | ER+AE vs CON | 0.21 (-0.07, 0.49) | 0.138 | No |
| SA3-excluding-high-RoB | Adiponectin | ER+HIIT vs CON | -0.08 (-0.72, 0.55) | 0.795 | No |
| SA3-excluding-high-RoB | Adiponectin | ER+COE vs CON | 0.80 (0.34, 1.25) | <0.001 | Yes |
| SA3-excluding-high-RoB | HOMA-IR | ER vs CON | -0.67 (-1.06, -0.28) | <0.001 | Yes |
| SA3-excluding-high-RoB | HOMA-IR | ER+AE vs CON | -0.87 (-1.20, -0.54) | <0.001 | Yes |
| SA3-excluding-high-RoB | HOMA-IR | ER+HIIT vs CON | -1.01 (-2.22, 0.20) | 0.102 | No |
| SA3-excluding-high-RoB | HOMA-IR | ER+COE vs CON | -0.94 (-1.38, -0.50) | <0.001 | Yes |
| SA4-longer-term-only | hs-CRP | ER vs CON | -0.42 (-0.71, -0.12) | 0.005 | Yes |
| SA4-longer-term-only | hs-CRP | ER+AE vs CON | -0.67 (-0.89, -0.44) | <0.001 | Yes |
| SA4-longer-term-only | CRP | ER vs CON | -0.08 (-0.45, 0.28) | 0.645 | No |
| SA4-longer-term-only | CRP | ER+AE vs CON | -0.76 (-1.55, 0.02) | 0.058 | No |
| SA4-longer-term-only | CRP | ER+COE vs CON | -0.04 (-0.38, 0.29) | 0.795 | No |
| SA4-longer-term-only | TNF-alpha | ER vs CON | 0.05 (-0.30, 0.39) | 0.791 | No |
| SA4-longer-term-only | TNF-alpha | ER+AE vs CON | -0.50 (-0.85, -0.15) | 0.005 | Yes |
| SA4-longer-term-only | TNF-alpha | ER+COE vs CON | -0.05 (-0.41, 0.32) | 0.802 | No |
| SA4-longer-term-only | IL-6 | ER vs CON | -0.55 (-0.87, -0.23) | <0.001 | Yes |
| SA4-longer-term-only | IL-6 | ER+AE vs CON | -0.83 (-1.15, -0.51) | <0.001 | Yes |
| SA4-longer-term-only | IL-6 | ER+COE vs CON | -0.25 (-0.72, 0.22) | 0.302 | No |
| SA4-longer-term-only | Adiponectin | ER vs CON | 0.06 (-0.44, 0.56) | 0.817 | No |
| SA4-longer-term-only | Adiponectin | ER+AE vs CON | 0.23 (-0.15, 0.61) | 0.232 | No |
| SA4-longer-term-only | Adiponectin | ER+COE vs CON | 0.86 (0.01, 1.71) | 0.048 | Yes |
| SA4-longer-term-only | HOMA-IR | ER vs CON | -0.51 (-1.18, 0.15) | 0.132 | No |
| SA4-longer-term-only | HOMA-IR | ER+AE vs CON | -0.80 (-1.26, -0.35) | <0.001 | Yes |
| SA5-BMI-ge30-only | hs-CRP | ER vs CON | -0.41 (-0.86, 0.04) | 0.072 | No |
| SA5-BMI-ge30-only | hs-CRP | ER+AE vs CON | -0.57 (-0.95, -0.18) | 0.004 | Yes |
| SA5-BMI-ge30-only | hs-CRP | ER+HIIT vs CON | -0.52 (-1.19, 0.15) | 0.127 | No |
| SA5-BMI-ge30-only | CRP | ER vs CON | -0.07 (-0.42, 0.29) | 0.717 | No |
| SA5-BMI-ge30-only | CRP | ER+AE vs CON | -0.78 (-1.32, -0.24) | 0.005 | Yes |
| SA5-BMI-ge30-only | CRP | ER+HIIT vs CON | -0.38 (-0.94, 0.18) | 0.187 | No |
| SA5-BMI-ge30-only | CRP | ER+COE vs CON | -0.00 (-0.29, 0.28) | 0.988 | No |
| SA5-BMI-ge30-only | TNF-alpha | ER vs CON | -0.08 (-0.44, 0.28) | 0.664 | No |
| SA5-BMI-ge30-only | TNF-alpha | ER+AE vs CON | -0.71 (-1.11, -0.30) | <0.001 | Yes |
| SA5-BMI-ge30-only | TNF-alpha | ER+HIIT vs CON | 0.12 (-0.48, 0.71) | 0.704 | No |
| SA5-BMI-ge30-only | TNF-alpha | ER+COE vs CON | -0.11 (-0.50, 0.29) | 0.600 | No |
| SA5-BMI-ge30-only | IL-6 | ER vs CON | -0.55 (-0.82, -0.27) | <0.001 | Yes |
| SA5-BMI-ge30-only | IL-6 | ER+AE vs CON | -0.88 (-1.15, -0.62) | <0.001 | Yes |
| SA5-BMI-ge30-only | IL-6 | ER+HIIT vs CON | -0.82 (-1.36, -0.28) | 0.003 | Yes |
| SA5-BMI-ge30-only | IL-6 | ER+COE vs CON | -0.24 (-0.67, 0.18) | 0.257 | No |
| SA5-BMI-ge30-only | Adiponectin | ER vs CON | -0.01 (-0.45, 0.42) | 0.952 | No |
| SA5-BMI-ge30-only | Adiponectin | ER+AE vs CON | 0.08 (-0.26, 0.42) | 0.652 | No |
| SA5-BMI-ge30-only | Adiponectin | ER+HIIT vs CON | -0.33 (-1.02, 0.37) | 0.353 | No |
| SA5-BMI-ge30-only | Adiponectin | ER+COE vs CON | 0.86 (0.01, 1.71) | 0.048 | Yes |
| SA5-BMI-ge30-only | HOMA-IR | ER vs CON | -0.32 (-0.78, 0.14) | 0.168 | No |
| SA5-BMI-ge30-only | HOMA-IR | ER+AE vs CON | -0.47 (-0.88, -0.05) | 0.028 | Yes |
| SA5-BMI-ge30-only | HOMA-IR | ER+HIIT vs CON | -0.67 (-1.36, 0.01) | 0.055 | No |

Note. SMD-95CI is presented as SMD (95% CI). For lower-is-better outcomes, negative SMDs favor the intervention; for adiponectin, positive SMDs favor the intervention.

**Supplementary Table 7.part 4. Between-study heterogeneity estimates.**

| **Sensitivity** | **Outcome** | **tau2** | **tau** | **I2** | **Q** | **df-Q** | **p-Q** |
| --- | --- | --- | --- | --- | --- | --- | --- |
| SA1-strict-ER | hs-CRP | 0.033 | 0.182 | 0.1 | 7.90 | 7 | 0.341 |
| SA1-strict-ER | CRP | 0.000 | 0.000 | 0.0 | 0.90 | 4 | 0.924 |
| SA1-strict-ER | TNF-alpha | 0.000 | 0.000 | 0.0 | 2.65 | 4 | 0.618 |
| SA1-strict-ER | IL-6 | 0.025 | 0.157 | 0.3 | 15.34 | 11 | 0.167 |
| SA1-strict-ER | Adiponectin | 0.000 | 0.000 | 0.0 | 2.19 | 3 | 0.535 |
| SA1-strict-ER | HOMA-IR | 0.000 | 0.000 | 0.0 | 2.87 | 7 | 0.897 |
| SA2-excluding-minors | hs-CRP | 0.017 | 0.130 | 0.0 | 6.93 | 8 | 0.544 |
| SA2-excluding-minors | CRP | 0.000 | 0.000 | 0.0 | 0.64 | 3 | 0.886 |
| SA2-excluding-minors | TNF-alpha | 0.000 | 0.000 | 0.0 | 4.08 | 4 | 0.395 |
| SA2-excluding-minors | IL-6 | 0.014 | 0.118 | 0.0 | 9.40 | 9 | 0.401 |
| SA2-excluding-minors | Adiponectin | 0.000 | 0.000 | 0.0 | 2.19 | 3 | 0.535 |
| SA2-excluding-minors | HOMA-IR | 0.043 | 0.209 | 0.4 | 9.71 | 6 | 0.138 |
| SA3-excluding-high-RoB | hs-CRP | 0.029 | 0.169 | 0.1 | 8.72 | 8 | 0.366 |
| SA3-excluding-high-RoB | CRP | 0.000 | 0.000 | 0.0 | 1.28 | 5 | 0.937 |
| SA3-excluding-high-RoB | TNF-alpha | 0.013 | 0.113 | 0.2 | 5.30 | 4 | 0.258 |
| SA3-excluding-high-RoB | IL-6 | 0.024 | 0.155 | 0.3 | 16.46 | 11 | 0.125 |
| SA3-excluding-high-RoB | Adiponectin | 0.000 | 0.000 | 0.0 | 3.16 | 5 | 0.676 |
| SA3-excluding-high-RoB | HOMA-IR | 0.044 | 0.209 | 0.4 | 10.43 | 6 | 0.107 |
| SA4-longer-term-only | hs-CRP | 0.023 | 0.151 | 0.0 | 5.52 | 6 | 0.479 |
| SA4-longer-term-only | CRP | 0.000 | 0.000 | 0.0 | 0.52 | 1 | 0.473 |
| SA4-longer-term-only | TNF-alpha | 0.000 | 0.000 | 0.0 | 0.27 | 2 | 0.876 |
| SA4-longer-term-only | IL-6 | 0.033 | 0.180 | 0.4 | 6.74 | 4 | 0.150 |
| SA4-longer-term-only | Adiponectin | 0.000 | 0.000 | 0.0 | 0.18 | 1 | 0.670 |
| SA4-longer-term-only | HOMA-IR | 0.105 | 0.324 | 0.7 | 8.87 | 3 | 0.031 |
| SA5-BMI-ge30-only | hs-CRP | 0.072 | 0.269 | 0.5 | 9.24 | 5 | 0.100 |
| SA5-BMI-ge30-only | CRP | 0.000 | 0.000 | 0.0 | 0.74 | 4 | 0.946 |
| SA5-BMI-ge30-only | TNF-alpha | 0.008 | 0.088 | 0.2 | 4.72 | 4 | 0.317 |
| SA5-BMI-ge30-only | IL-6 | 0.020 | 0.140 | 0.2 | 9.73 | 8 | 0.285 |
| SA5-BMI-ge30-only | Adiponectin | 0.000 | 0.000 | 0.0 | 0.13 | 2 | 0.935 |
| SA5-BMI-ge30-only | HOMA-IR | 0.000 | 0.000 | 0.0 | 0.17 | 4 | 0.997 |

Note. tau2 and tau are between-study heterogeneity estimates from the random-effects network meta-analysis. I2 is reported as returned by netmeta.

**Supplementary Table 7.part 5.League table of sensitivity analysis**

**Table. SA1-strict-ER — hs-CRP league table.**

| **ER+HIIT** |  |  |  |
| --- | --- | --- | --- |
| 0.22 (-0.34, 0.78) | **ER+AE** |  |  |
| -0.09 (-0.56, 0.38) | **-0.31 (-0.62, -0.01)** | **ER** |  |
| -0.49 (-1.06, 0.08) | **-0.71 (-1.01, -0.41)** | **-0.40 (-0.72, -0.07)** | **CON** |

Note: For this outcome, lower values are favorable; negative SMDs favor the column-defining treatment. Bold estimates indicate 95% CI not crossing 0.

**Table. SA1-strict-ER — CRP league table.**

| **ER+COE** |  |  |  |  |
| --- | --- | --- | --- | --- |
| 0.38 (-0.15, 0.92) | **ER+HIIT** |  |  |  |
| **0.59 (0.09, 1.09)** | 0.20 (-0.43, 0.84) | **ER+AE** |  |  |
| 0.07 (-0.23, 0.37) | -0.31 (-0.75, 0.13) | **-0.52 (-0.98, -0.06)** | **ER** |  |
| -0.04 (-0.38, 0.30) | -0.42 (-0.97, 0.12) | **-0.63 (-1.07, -0.19)** | -0.11 (-0.43, 0.21) | **CON** |

Note: For this outcome, lower values are favorable; negative SMDs favor the column-defining treatment. Bold estimates indicate 95% CI not crossing 0.

**Table. SA1-strict-ER — TNF-alpha league table.**

| **ER+COE** |  |  |  |  |
| --- | --- | --- | --- | --- |
| -0.49 (-1.18, 0.19) | **ER+HIIT** |  |  |  |
| **0.49 (0.01, 0.97)** | **0.98 (0.28, 1.69)** | **ER+AE** |  |  |
| -0.06 (-0.43, 0.31) | 0.44 (-0.14, 1.01) | **-0.55 (-0.95, -0.14)** | **ER** |  |
| -0.08 (-0.44, 0.28) | 0.42 (-0.25, 1.08) | **-0.56 (-0.94, -0.19)** | -0.02 (-0.35, 0.31) | **CON** |

Note: For this outcome, lower values are favorable; negative SMDs favor the column-defining treatment. Bold estimates indicate 95% CI not crossing 0.

**Table. SA1-strict-ER — IL-6 league table.**

| **ER+COE** |  |  |  |  |
| --- | --- | --- | --- | --- |
| 0.28 (-0.32, 0.88) | **ER+HIIT** |  |  |  |
| **0.49 (0.11, 0.87)** | 0.21 (-0.37, 0.79) | **ER+AE** |  |  |
| 0.11 (-0.20, 0.42) | -0.17 (-0.68, 0.35) | **-0.38 (-0.65, -0.11)** | **ER** |  |
| **-0.36 (-0.69, -0.02)** | **-0.64 (-1.21, -0.06)** | **-0.85 (-1.12, -0.58)** | **-0.47 (-0.72, -0.21)** | **CON** |

Note: For this outcome, lower values are favorable; negative SMDs favor the column-defining treatment. Bold estimates indicate 95% CI not crossing 0.

**Table. SA1-strict-ER — Adiponectin league table.**

| **ER+COE** |  |  |  |  |
| --- | --- | --- | --- | --- |
| **0.84 (0.07, 1.60)** | **ER+HIIT** |  |  |  |
| 0.49 (-0.11, 1.08) | -0.35 (-1.00, 0.30) | **ER+AE** |  |  |
| 0.52 (-0.02, 1.06) | -0.31 (-0.85, 0.22) | 0.03 (-0.32, 0.39) | **ER** |  |
| **0.80 (0.25, 1.34)** | -0.04 (-0.69, 0.61) | 0.31 (-0.04, 0.66) | 0.28 (-0.08, 0.63) | **CON** |

Note: For adiponectin, higher values are favorable; positive SMDs favor the column-defining treatment. Bold estimates indicate 95% CI not crossing 0.

**Table. SA1-strict-ER — HOMA-IR league table.**

| **ER+COE** |  |  |  |  |
| --- | --- | --- | --- | --- |
| 0.02 (-0.66, 0.70) | **ER+HIIT** |  |  |  |
| -0.23 (-0.66, 0.20) | -0.25 (-0.91, 0.41) | **ER+AE** |  |  |
| -0.33 (-0.68, 0.02) | -0.36 (-0.94, 0.23) | -0.10 (-0.41, 0.20) | **ER** |  |
| **-0.84 (-1.20, -0.47)** | **-0.86 (-1.53, -0.19)** | **-0.61 (-0.96, -0.25)** | **-0.50 (-0.83, -0.18)** | **CON** |

Note: For this outcome, lower values are favorable; negative SMDs favor the column-defining treatment. Bold estimates indicate 95% CI not crossing 0.

**Table. SA2-excluding-minors — hs-CRP league table.**

| **ER+HIIT** |  |  |  |
| --- | --- | --- | --- |
| 0.03 (-0.56, 0.61) | **ER+AE** |  |  |
| -0.28 (-0.81, 0.24) | **-0.31 (-0.57, -0.05)** | **ER** |  |
| **-0.65 (-1.24, -0.07)** | **-0.68 (-0.89, -0.47)** | **-0.37 (-0.64, -0.10)** | **CON** |

Note: For this outcome, lower values are favorable; negative SMDs favor the column-defining treatment. Bold estimates indicate 95% CI not crossing 0.

**Table. SA2-excluding-minors — CRP league table.**

| **ER+COE** |  |  |  |  |
| --- | --- | --- | --- | --- |
| 0.43 (-0.14, 0.99) | **ER+HIIT** |  |  |  |
| 0.57 (-0.01, 1.14) | 0.14 (-0.54, 0.82) | **ER+AE** |  |  |
| 0.11 (-0.24, 0.47) | -0.31 (-0.75, 0.13) | -0.45 (-0.97, 0.06) | **ER** |  |
| 0.03 (-0.27, 0.32) | -0.40 (-0.96, 0.15) | **-0.54 (-1.08, -0.01)** | -0.09 (-0.42, 0.25) | **CON** |

Note: For this outcome, lower values are favorable; negative SMDs favor the column-defining treatment. Bold estimates indicate 95% CI not crossing 0.

**Table. SA2-excluding-minors — TNF-alpha league table.**

| **ER+COE** |  |  |  |  |
| --- | --- | --- | --- | --- |
| -0.23 (-0.85, 0.38) | **ER+HIIT** |  |  |  |
| 0.36 (-0.15, 0.87) | 0.59 (-0.06, 1.25) | **ER+AE** |  |  |
| -0.04 (-0.41, 0.33) | 0.19 (-0.32, 0.71) | -0.40 (-0.83, 0.03) | **ER** |  |
| -0.09 (-0.45, 0.26) | 0.14 (-0.43, 0.71) | **-0.46 (-0.88, -0.03)** | -0.06 (-0.38, 0.27) | **CON** |

Note: For this outcome, lower values are favorable; negative SMDs favor the column-defining treatment. Bold estimates indicate 95% CI not crossing 0.

**Table. SA2-excluding-minors — IL-6 league table.**

| **ER+COE** |  |  |  |  |
| --- | --- | --- | --- | --- |
| 0.47 (-0.09, 1.03) | **ER+HIIT** |  |  |  |
| **0.63 (0.23, 1.03)** | 0.16 (-0.36, 0.68) | **ER+AE** |  |  |
| 0.19 (-0.14, 0.53) | -0.27 (-0.73, 0.18) | **-0.44 (-0.69, -0.18)** | **ER** |  |
| **-0.36 (-0.69, -0.02)** | **-0.83 (-1.34, -0.31)** | **-0.99 (-1.28, -0.70)** | **-0.55 (-0.80, -0.31)** | **CON** |

Note: For this outcome, lower values are favorable; negative SMDs favor the column-defining treatment. Bold estimates indicate 95% CI not crossing 0.

**Table. SA2-excluding-minors — Adiponectin league table.**

| **ER+COE** |  |  |  |  |
| --- | --- | --- | --- | --- |
| **0.84 (0.07, 1.60)** | **ER+HIIT** |  |  |  |
| 0.49 (-0.11, 1.08) | -0.35 (-1.00, 0.30) | **ER+AE** |  |  |
| 0.52 (-0.02, 1.06) | -0.31 (-0.85, 0.22) | 0.03 (-0.32, 0.39) | **ER** |  |
| **0.80 (0.25, 1.34)** | -0.04 (-0.69, 0.61) | 0.31 (-0.04, 0.66) | 0.28 (-0.08, 0.63) | **CON** |

Note: For adiponectin, higher values are favorable; positive SMDs favor the column-defining treatment. Bold estimates indicate 95% CI not crossing 0.

**Table. SA2-excluding-minors — HOMA-IR league table.**

| **ER+COE** |  |  |  |  |
| --- | --- | --- | --- | --- |
| -0.02 (-1.33, 1.30) | **ER+HIIT** |  |  |  |
| -0.10 (-0.78, 0.58) | -0.08 (-1.28, 1.12) | **ER+AE** |  |  |
| -0.36 (-1.01, 0.30) | -0.34 (-1.48, 0.81) | -0.26 (-0.61, 0.09) | **ER** |  |
| **-0.95 (-1.60, -0.30)** | -0.93 (-2.14, 0.27) | **-0.85 (-1.18, -0.53)** | **-0.60 (-0.98, -0.21)** | **CON** |

Note: For this outcome, lower values are favorable; negative SMDs favor the column-defining treatment. Bold estimates indicate 95% CI not crossing 0.

**Table. SA3-excluding-high-RoB — hs-CRP league table.**

| **ER+HIIT** |  |  |  |
| --- | --- | --- | --- |
| -0.07 (-0.70, 0.55) | **ER+AE** |  |  |
| -0.28 (-0.83, 0.27) | -0.21 (-0.51, 0.09) | **ER** |  |
| **-0.67 (-1.30, -0.04)** | **-0.60 (-0.82, -0.38)** | **-0.39 (-0.69, -0.09)** | **CON** |

Note: For this outcome, lower values are favorable; negative SMDs favor the column-defining treatment. Bold estimates indicate 95% CI not crossing 0.

**Table. SA3-excluding-high-RoB — CRP league table.**

| **ER+COE** |  |  |  |  |
| --- | --- | --- | --- | --- |
| 0.37 (-0.16, 0.90) | **ER+HIIT** |  |  |  |
| **0.56 (0.07, 1.05)** | 0.19 (-0.44, 0.82) | **ER+AE** |  |  |
| 0.05 (-0.24, 0.35) | -0.31 (-0.75, 0.13) | **-0.51 (-0.96, -0.05)** | **ER** |  |
| -0.08 (-0.40, 0.23) | -0.45 (-0.99, 0.09) | **-0.64 (-1.07, -0.20)** | -0.13 (-0.45, 0.18) | **CON** |

Note: For this outcome, lower values are favorable; negative SMDs favor the column-defining treatment. Bold estimates indicate 95% CI not crossing 0.

**Table. SA3-excluding-high-RoB — TNF-alpha league table.**

| **ER+COE** |  |  |  |  |
| --- | --- | --- | --- | --- |
| -0.21 (-0.87, 0.45) | **ER+HIIT** |  |  |  |
| 0.47 (-0.09, 1.03) | 0.68 (-0.04, 1.40) | **ER+AE** |  |  |
| -0.01 (-0.45, 0.42) | 0.20 (-0.34, 0.73) | -0.48 (-1.03, 0.06) | **ER** |  |
| -0.12 (-0.54, 0.31) | 0.09 (-0.52, 0.71) | **-0.59 (-0.95, -0.22)** | -0.10 (-0.51, 0.30) | **CON** |

Note: For this outcome, lower values are favorable; negative SMDs favor the column-defining treatment. Bold estimates indicate 95% CI not crossing 0.

**Table. SA3-excluding-high-RoB — IL-6 league table.**

| **ER+COE** |  |  |  |  |
| --- | --- | --- | --- | --- |
| 0.38 (-0.18, 0.94) | **ER+HIIT** |  |  |  |
| **0.43 (0.05, 0.81)** | 0.05 (-0.50, 0.60) | **ER+AE** |  |  |
| 0.11 (-0.21, 0.42) | -0.27 (-0.74, 0.20) | **-0.32 (-0.61, -0.04)** | **ER** |  |
| **-0.35 (-0.68, -0.02)** | **-0.73 (-1.26, -0.19)** | **-0.78 (-1.03, -0.52)** | **-0.45 (-0.71, -0.20)** | **CON** |

Note: For this outcome, lower values are favorable; negative SMDs favor the column-defining treatment. Bold estimates indicate 95% CI not crossing 0.

**Table. SA3-excluding-high-RoB — Adiponectin league table.**

| **ER+COE** |  |  |  |  |
| --- | --- | --- | --- | --- |
| **0.88 (0.15, 1.61)** | **ER+HIIT** |  |  |  |
| **0.59 (0.08, 1.09)** | -0.29 (-0.93, 0.34) | **ER+AE** |  |  |
| **0.57 (0.08, 1.05)** | -0.31 (-0.85, 0.22) | -0.02 (-0.36, 0.32) | **ER** |  |
| **0.80 (0.34, 1.25)** | -0.08 (-0.72, 0.55) | 0.21 (-0.07, 0.49) | 0.23 (-0.11, 0.57) | **CON** |

Note: For adiponectin, higher values are favorable; positive SMDs favor the column-defining treatment. Bold estimates indicate 95% CI not crossing 0.

**Table. SA3-excluding-high-RoB — HOMA-IR league table.**

| **ER+COE** |  |  |  |  |
| --- | --- | --- | --- | --- |
| 0.07 (-1.15, 1.29) | **ER+HIIT** |  |  |  |
| -0.07 (-0.57, 0.44) | -0.14 (-1.35, 1.08) | **ER+AE** |  |  |
| -0.27 (-0.70, 0.16) | -0.34 (-1.48, 0.81) | -0.20 (-0.61, 0.20) | **ER** |  |
| **-0.94 (-1.38, -0.50)** | -1.01 (-2.22, 0.20) | **-0.87 (-1.20, -0.54)** | **-0.67 (-1.06, -0.28)** | **CON** |

Note: For this outcome, lower values are favorable; negative SMDs favor the column-defining treatment. Bold estimates indicate 95% CI not crossing 0.

**Table. SA4-longer-term-only — hs-CRP league table.**

| **ER+AE** |  |  |
| --- | --- | --- |
| -0.25 (-0.54, 0.04) | **ER** |  |
| **-0.67 (-0.89, -0.44)** | **-0.42 (-0.71, -0.12)** | **CON** |

Note: For this outcome, lower values are favorable; negative SMDs favor the column-defining treatment. Bold estimates indicate 95% CI not crossing 0.

**Table. SA4-longer-term-only — CRP league table.**

| **ER+COE** |  |  |  |
| --- | --- | --- | --- |
| 0.72 (-0.14, 1.57) | **ER+AE** |  |  |
| 0.04 (-0.33, 0.41) | -0.68 (-1.54, 0.19) | **ER** |  |
| -0.04 (-0.38, 0.29) | -0.76 (-1.55, 0.02) | -0.08 (-0.45, 0.28) | **CON** |

Note: For this outcome, lower values are favorable; negative SMDs favor the column-defining treatment. Bold estimates indicate 95% CI not crossing 0.

**Table. SA4-longer-term-only — TNF-alpha league table.**

| **ER+COE** |  |  |  |
| --- | --- | --- | --- |
| 0.45 (-0.03, 0.94) | **ER+AE** |  |  |
| -0.09 (-0.47, 0.28) | **-0.54 (-0.99, -0.10)** | **ER** |  |
| -0.05 (-0.41, 0.32) | **-0.50 (-0.85, -0.15)** | 0.05 (-0.30, 0.39) | **CON** |

Note: For this outcome, lower values are favorable; negative SMDs favor the column-defining treatment. Bold estimates indicate 95% CI not crossing 0.

**Table. SA4-longer-term-only — IL-6 league table.**

| **ER+COE** |  |  |  |
| --- | --- | --- | --- |
| **0.58 (0.05, 1.12)** | **ER+AE** |  |  |
| 0.30 (-0.17, 0.78) | -0.28 (-0.63, 0.07) | **ER** |  |
| -0.25 (-0.72, 0.22) | **-0.83 (-1.15, -0.51)** | **-0.55 (-0.87, -0.23)** | **CON** |

Note: For this outcome, lower values are favorable; negative SMDs favor the column-defining treatment. Bold estimates indicate 95% CI not crossing 0.

**Table. SA4-longer-term-only — Adiponectin league table.**

| **ER+COE** |  |  |  |
| --- | --- | --- | --- |
| 0.63 (-0.30, 1.56) | **ER+AE** |  |  |
| 0.80 (-0.19, 1.79) | 0.17 (-0.33, 0.67) | **ER** |  |
| **0.86 (0.01, 1.71)** | 0.23 (-0.15, 0.61) | 0.06 (-0.44, 0.56) | **CON** |

Note: For adiponectin, higher values are favorable; positive SMDs favor the column-defining treatment. Bold estimates indicate 95% CI not crossing 0.

**Table. SA4-longer-term-only — HOMA-IR league table.**

| **ER+AE** |  |  |
| --- | --- | --- |
| -0.29 (-0.91, 0.32) | **ER** |  |
| **-0.80 (-1.26, -0.35)** | -0.51 (-1.18, 0.15) | **CON** |

Note: For this outcome, lower values are favorable; negative SMDs favor the column-defining treatment. Bold estimates indicate 95% CI not crossing 0.

**Table. SA5-BMI-ge30-only — hs-CRP league table.**

| **ER+HIIT** |  |  |  |
| --- | --- | --- | --- |
| 0.05 (-0.62, 0.71) | **ER+AE** |  |  |
| -0.11 (-0.60, 0.39) | -0.15 (-0.60, 0.29) | **ER** |  |
| -0.52 (-1.19, 0.15) | **-0.57 (-0.95, -0.18)** | -0.41 (-0.86, 0.04) | **CON** |

Note: For this outcome, lower values are favorable; negative SMDs favor the column-defining treatment. Bold estimates indicate 95% CI not crossing 0.

**Table. SA5-BMI-ge30-only — CRP league table.**

| **ER+COE** |  |  |  |  |
| --- | --- | --- | --- | --- |
| 0.38 (-0.19, 0.94) | **ER+HIIT** |  |  |  |
| **0.78 (0.17, 1.39)** | 0.40 (-0.38, 1.18) | **ER+AE** |  |  |
| 0.06 (-0.30, 0.42) | -0.31 (-0.75, 0.13) | **-0.72 (-1.36, -0.07)** | **ER** |  |
| -0.00 (-0.29, 0.28) | -0.38 (-0.94, 0.18) | **-0.78 (-1.32, -0.24)** | -0.07 (-0.42, 0.29) | **CON** |

Note: For this outcome, lower values are favorable; negative SMDs favor the column-defining treatment. Bold estimates indicate 95% CI not crossing 0.

**Table. SA5-BMI-ge30-only — TNF-alpha league table.**

| **ER+COE** |  |  |  |  |
| --- | --- | --- | --- | --- |
| -0.22 (-0.86, 0.42) | **ER+HIIT** |  |  |  |
| **0.60 (0.06, 1.14)** | **0.82 (0.13, 1.51)** | **ER+AE** |  |  |
| -0.03 (-0.43, 0.38) | 0.20 (-0.33, 0.72) | **-0.63 (-1.12, -0.14)** | **ER** |  |
| -0.11 (-0.50, 0.29) | 0.12 (-0.48, 0.71) | **-0.71 (-1.11, -0.30)** | -0.08 (-0.44, 0.28) | **CON** |

Note: For this outcome, lower values are favorable; negative SMDs favor the column-defining treatment. Bold estimates indicate 95% CI not crossing 0.

**Table. SA5-BMI-ge30-only — IL-6 league table.**

| **ER+COE** |  |  |  |  |
| --- | --- | --- | --- | --- |
| 0.58 (-0.05, 1.20) | **ER+HIIT** |  |  |  |
| **0.64 (0.17, 1.10)** | 0.06 (-0.48, 0.60) | **ER+AE** |  |  |
| 0.30 (-0.13, 0.73) | -0.27 (-0.74, 0.19) | **-0.34 (-0.61, -0.06)** | **ER** |  |
| -0.24 (-0.67, 0.18) | **-0.82 (-1.36, -0.28)** | **-0.88 (-1.15, -0.62)** | **-0.55 (-0.82, -0.27)** | **CON** |

Note: For this outcome, lower values are favorable; negative SMDs favor the column-defining treatment. Bold estimates indicate 95% CI not crossing 0.

**Table. SA5-BMI-ge30-only — Adiponectin league table.**

| **ER+COE** |  |  |  |  |
| --- | --- | --- | --- | --- |
| **1.19 (0.09, 2.28)** | **ER+HIIT** |  |  |  |
| 0.78 (-0.14, 1.70) | -0.41 (-1.07, 0.25) | **ER+AE** |  |  |
| 0.87 (-0.08, 1.83) | -0.31 (-0.85, 0.22) | 0.09 (-0.29, 0.47) | **ER** |  |
| **0.86 (0.01, 1.71)** | -0.33 (-1.02, 0.37) | 0.08 (-0.26, 0.42) | -0.01 (-0.45, 0.42) | **CON** |

Note: For adiponectin, higher values are favorable; positive SMDs favor the column-defining treatment. Bold estimates indicate 95% CI not crossing 0.

**Table. SA5-BMI-ge30-only — HOMA-IR league table.**

| **ER+HIIT** |  |  |  |
| --- | --- | --- | --- |
| -0.21 (-0.83, 0.42) | **ER+AE** |  |  |
| -0.35 (-0.87, 0.16) | -0.14 (-0.50, 0.21) | **ER** |  |
| -0.67 (-1.36, 0.01) | **-0.47 (-0.88, -0.05)** | -0.32 (-0.78, 0.14) | **CON** |

Note: For this outcome, lower values are favorable; negative SMDs favor the column-defining treatment. Bold estimates indicate 95% CI not crossing 0.

**Supplementary Figure 5.** Exploratory study-level meta-regression analyses of mean age and intervention duration modified treatment effects across the network
